# Alignment-free similarity analysis for protein sequences based on fuzzy integral

**DOI:** 10.1038/s41598-019-39477-8

**Published:** 2019-02-26

**Authors:** Ajay Kumar Saw, Binod Chandra Tripathy, Soumyadeep Nandi

**Affiliations:** 1 Institute of Advanced Study in Science and Technology, Mathematical Sciences Division, Guwahati, 781035 India; 2 Tripura University, Department of Mathematics, Agartala, 799022 India; 3 Institute of Advanced Study in Science and Technology, Life Science Division, Guwahati, 781035 India

## Abstract

Sequence comparison is an essential part of modern molecular biology research. In this study, we estimated the parameters of Markov chain by considering the frequencies of occurrence of the all possible amino acid pairs from each alignment-free protein sequence. These estimated Markov chain parameters were used to calculate similarity between two protein sequences based on a fuzzy integral algorithm. For validation, our result was compared with both alignment-based (ClustalW) and alignment-free methods on six benchmark datasets. The results indicate that our developed algorithm has a better clustering performance for protein sequence comparison.

## Introduction

With the advent of the advanced sequencing techniques, researchers are generating a large number of protein sequences. This brings in a new challenge^1,2^ for phylogenetic and comparative study of these protein sequences. Phylogenetic study and comparative analysis between taxa are an essential part of molecular biology and bioinformatics. These studies, traditionally depended on multiple or pairwise sequence alignments which are the well established classical approach and regarded as a standard method for sequence analysis. However, producing reliable multiple sequence alignments become extremely difficult when more dissimilar protein sequences are considered. The traditional alignment-based methods^3–5^ are much empirical to select and create a sequence alignment score matrix, and variation of which may affect the alignment results. Various alignment-free tools^6–13^ have been developed over the past two decades to overcome the alignment complexity for phylogenetic analysis. An alignment-free approach consist of two steps for comparing protein sequences. At the first step, the protein sequences are converted into a fixed-length feature vectors. Feature extraction is a series of process for extracting the required information from the query sequences, which is critical for the accuracy of an alignment-free method. At the second step, these extracted feature vectors are used as an input data in vectors similarity comparison algorithm to perform downstream analysis like phylogenetic analysis. Methods based on graphical representation, distance frequency matrix, numerical characterization, K-string dictionary etc., have been introduced to overcome the complication of the sequence alignment. Graphical representation^14,15^ of protein sequences provides a simple way of viewing, sorting and comparing various sequences. It also provides mathematical descriptor which help in identifying differences among similar protein sequences quantitatively. Distance frequency of amino acid pairs suggest a new numerical characterization of protein sequence, which converts protein sequence into a distance frequency matrix^16^. Numerical characterization directly extracted from protein sequence would capture the essence of the amino acid composition and their distribution on the protein sequence in a quantitative aspect. In this approach, each sequence is mapped into a vector or matrix based on the numerical characterization extracted from the protein sequence. Subsequently, a similarity score is calculated by following distance measure tools, such as, Euclidean distance, Cosine distance, Manhattan distance, etc., among their corresponding vectors or matrices. K-string dictionary^17^ approach permit users to use a much lower dimensional frequency or probability vector to represent a protein sequence. It also significantly reduces the space requirement for their implementation. Furthermore, after getting the lower dimensional frequency vectors, Singular Value Decomposition (SVD) is used to get a better protein vector representation which helps user to obtain a precise phylogenetic tree. However, these above mentioned methods are lagging behind in terms of accuracy. Thus, more discriminatory features are still needed to be developed. In addition to the accuracy, these method have another drawback and that is, computational complexity. Motivated by the aforementioned work, in this study, we proposed to use fuzzy integral algorithm^18,19^ for analysis of protein sequence based on Markov chain^20^. Fuzzy integral similarity^21,22^ method assigns similarity score within the closed interval [0, 1] between two protein sequences. A protein sequence consists of twenty amino acids. By taking these 20 amino acids as a state space *M* = {*A*, *I*, *L*, *M*, *F*, *P*, *W*, *V*, *D*, *E*, *N*, *C*, *Q*, *G*, *S*, *T*, *Y*, *R*, *H*, *K*}, we have used *k*^*th*^−step transition probability matrix, fuzzy measure^23^, fuzzy integral to describe protein sequence. We have used fuzzy integral similarity for getting distance matrix, which is used in neighbor program in PHYLIP package^24^ for constructing a phylogenetic tree. The advantage of our method is, it do not require any prior knowledge of homologous relationship (common ancestry) among the sequences, which makes it fully automated and robust. For validation of our developed algorithm, we implemented our approach on NADH Dehydrogenase-5 protein sequences, NADH Dehydrogenase-6 protein sequences, xylanases protein sequences in the F10 and G11 datasets, transferrin protein sequences, coronavirus spike protein sequences and beta-globin protein sequences. We compared the tree generated by our method with the trees generated by both alignment-free method, and alignment-based ClustalW method using MEGA package^25^. In addition, we used few standard statistical tools such as correlation coefficient (CC), Robinson-Foulds distance (RF-distance)^26^ and receiver operating characteristic (ROC)^27–29^ curve to compare distance matrices generated by our method with the other alignment-free methods. The main purpose of this study is to compare the performance among alignment-based and alignment-free protein clustering methods and to identify their strengths and weakness from the practical perspectives of the users.

## Methods

### Markov chain for protein sequence

Let *P* = [*p*_*i*,*j*_] represent the transition probability matrix of a discrete-time Markov chain^20^. Transition probability *p*_*i*,*j*_ can be defined as follows:

1 $${p}_{i,j}=p({Z}_{n+1}={a}_{j}|{Z}_{n}={a}_{i}),\,1\le i,\,j\le M,$$

where *Z*_*n*_ represent the actual state at time *n*(*n* = 1, 2, 3 ...), *a*_*i*_ is the *i*_*th*_ state within 20 distinct states. In the context of protein sequence, the number of states is *M* = 20, which corresponds to the twenty amino acids symbol set *M* = {*A* = *a*_1_, *I* = *a*_2_, *L* = *a*_3_, *M* = *a*_4_, *F* = *a*_5_, *P* = *a*_6_, *W* = *a*_7_, *V* = *a*_8_, *D* = *a*_9_, *E* = *a*_10_, *N* = *a*_11_, *C* = *a*_12_, *Q* = *a*_13_, *G* = *a*_14_, *S* = *a*_15_, *T* = *a*_16_, *Y* = *a*_17_, *R* = *a*_18_, *H* = *a*_19_, *K* = *a*_20_}. The state transition probabilities satisfy the following constraints

$${p}_{i,j}\ge 0\forall \,i,j\,{\rm{and}}\,\,\sum _{j\mathrm{=1}}^{M}\,{p}_{i,j}=1\forall \,i.$$

We calculated the transition probability matrices based on the observed sequences. From each alignment-free protein sequence, we assumed that the frequency of occurrences of all possible amino acid pairs as the parameters of Markov chain. If ${N}_{{a}_{i}{a}_{j}}$ denotes the total number of adjacent amino acid pair (*a*_*i*_, *a*_*j*_), then 1^*st*^−step transition probability matrix from the state *a*_*i*_ to the state *a*_*j*_ is given by

2 $${p}_{i,j}=\frac{{N}_{{a}_{i}{a}_{j}}}{\sum _{j=1}^{M}\,{N}_{{a}_{i}{a}_{j}}}$$

Above explanation is the 1^*st*^ step Markov chain and the *k*^*th*^ step Markov chain can be obtained through the 1^*st*^ step Markov chain. Let ${P}^{k}=[{p}_{i,j}^{k}]$ denote the transition probability matrix of a discrete-time Markov chain starting from state *i* after *k* steps to end with state *j*. Each state transition probability ${p}_{i,j}^{k}$ is given as follows:

3 $${p}_{i,j}^{k}={p}^{k}({Z}_{n+k}={a}_{j}|{Z}_{n}={a}_{i}),\,1\le i,\,j\le M,$$

satisfy following constraints

$${p}_{i,j}^{k}\ge 0\forall \,i,j\,{\rm{and}}\,\,\sum _{j\mathrm{=1}}^{M}\,{p}_{i,j}^{k}=1\forall \,i\mathrm{.}$$

For three sets *U*, *V* and *W*, the following condition holds: *p*[*U* ∩ *V*|*W*] = *p*[*U*|*V* ∩ *W*]*p*[*V*|*W*]. Interpreting *U* as *Z*_*n*+*k*_ = *a*_*j*_, *V* as *Z*_*n*+*t*_ = *a*_*r*_ and *W* as *Z*_*n*_ = *a*_*i*_, we have

4 $$\begin{array}{rcl}{p}_{i,j}^{k} & = & p[{Z}_{n+k}={a}_{j}|{Z}_{n}={a}_{i}]\\  & = & \sum _{{a}_{r}\in {\bf{M}}}\,p[{Z}_{n+k}={a}_{j},{Z}_{n+t}={a}_{r}|{Z}_{n}={a}_{i}]\\  & = & \sum _{{a}_{r}\in {\bf{M}}}\,p[{Z}_{n+k}={a}_{j}|{Z}_{n+t}={a}_{r},{Z}_{n}={a}_{i}]\\  &  & \times \,p[{Z}_{n+t}={a}_{r}|{Z}_{n}={a}_{i}]\\  & = & \sum _{{a}_{r}\in {\bf{M}}}\,p[{Z}_{n+k}={a}_{j}|{Z}_{n+t}={a}_{r}]\\  &  & \times p[{Z}_{n+t}={a}_{r}|{Z}_{n}={a}_{i}]\\  & = & \sum _{{a}_{r}\in {\bf{M}}}\,{p}_{r,j}^{k-t}{p}_{i,r}^{t},\end{array}$$

which is known as the Chapman-Kolmogorov equation.

Hence, the matrix with element ${p}_{i,j}^{k}$ are $[{p}_{i,j}^{k}]={P}^{k}\mathrm{.}$

In the context of protein sequence, *k*^*th*^-step transition probability matrix can be expressed as:

$${P}^{k}={[\begin{array}{ccccc}{p}_{\mathrm{1,1}}^{k} & {p}_{\mathrm{1,2}}^{k} & \mathrm{...} & \mathrm{...} & {p}_{\mathrm{1,20}}^{k}\\ {p}_{\mathrm{2,1}}^{k} & {p}_{\mathrm{2,2}}^{k} & \mathrm{...} & \mathrm{...} & {p}_{\mathrm{2,20}}^{k}\\ \mathrm{.} & \mathrm{.} & \mathrm{...} & \mathrm{...} & \mathrm{.}\\ \mathrm{.} & \mathrm{.} & \mathrm{...} & \mathrm{...} & \mathrm{.}\\ \mathrm{.} & \mathrm{.} & \mathrm{...} & \mathrm{...} & \mathrm{.}\\ {p}_{\mathrm{20,1}}^{k} & {p}_{\mathrm{20,2}}^{k} & \mathrm{...} & \mathrm{...} & {p}_{\mathrm{20,20}}^{k}\end{array}]}_{20\times 20}$$

which is subjected to ${p}_{i,j}^{k}\ge 0\forall \,i,j\in \mathrm{\{1,}\,\mathrm{2,}\,\mathrm{...20\}}$ and ${\sum }_{j=1}^{20}\,{p}_{i,j}^{k}=1\forall \,i\mathrm{.}$ The ${p}_{i,j}^{k}$ can be determined by the equations (2) and (4). After the derivation of the *k*^*th*^-step transition probability matrix, we optimized the step *k* = *h*, which is least positive integer under satisfying following condition for each protein sequence:

5 $$rmsd({P}^{k=h}-{P}^{k=h+1})\approx 0\,({\rm{upto}}\,{\rm{six}}\,{\rm{decimal}}\,{\rm{place}}),$$

where *rmsd* represent root mean square distance between two consecutive transition probability matrices. After optimizing the step of transition probability matrix, i.e, *P*^*h*^. We noted that, all 20 rows in optimized transition probability matrix are approximately identical with each other. Therefore, we took a single row from the transition probability matrix *P*^*h*^ as a input for further investigation, which reduced our time complexity.

### Fuzzy integral and fuzzy measure for the *h*^*th*^– step amino acids sequence

Let *G* = {(*ba*_*i*_)^*h*^ = *x*_*i*_|*i* ∈ {1, 2, 3, ..., 20}, *b* ∈ **M**} be the finite set of *h*^*th*^–step amino acids starting from amino acid *b* and ending with amino acid *a*_*i*_, estimated from protein sequence. The finite set *G* is termed as feature vector.

Let *ν*, *τ* ⊆ *G* and *R*(*G*) be the power set of *G*. A fuzzy measure *μ* is a real valued function:

*μ*: *R*(*G*) → [0, 1], satisfy the following condition,

(i) $$\mu (\varphi )=0\,{\rm{and}}\,\mu (G)=1$$
(ii) $$\mu (\nu )\le \mu (\tau )\,if\,\nu \subseteq \tau .$$

For a fuzzy measure *μ*, let *μ*(*x*_*i*_) = *μ*^*i*^ ∀ *x*_*i*_ ∈ *G*. The mapping *x*_*i*_ → *μ*^*i*^ is known as fuzzy density function. The fuzzy density of single element *x*_*i*_ ∈ *G*, *μ*^*i*^ can be interpreted as the importance of *x*_*i*_ in determining the set *G*. Based on the fuzzy measure definition *μ*, the measure of a subset is not just only the summation of the measure of its elements but also included the measure of each combination. This information could be delivered by an expert or observed through the problem. However, when handing with larger set, this job may become computationally complex, difficult or even not feasible. *λ*-measures is the possible solution for solving this problem. *λ*-fuzzy measure^30^ fulfills the criteria of fuzzy measure plus some additional property: for all *ν*, *τ* ⊂ *G*, *ν* ∩ *τ* = *ϕ* and

6 $$\mu (\nu \cup \tau )=\mu (\nu )+\mu (\tau )+\lambda \mu (\nu )\mu (\tau ),\,\,{\rm{for}}\,{\rm{some}}\,\lambda  > -\,1.$$

Furthermore, *λ* can be obtained by solving following equation:

7 $$\lambda +1=\prod _{i=1}^{20}\,\mathrm{(1}+\lambda {\mu }^{i}\mathrm{).}$$

Therefore, we can construct fuzzy measure by applying equation(6) and equation(7), for this we only need to know the individual fuzzy densities of the elements *μ*^*i*^ (∀ *i* ∈ {1, 2, 3, ..., 20}).

Let *ρ*:*G* → [0, 1] represent a function that maps every element of *G* to its evidence. The function *ρ* must satisfy descending order, which is as follows: *ρ*(*x*_1_) ≥ *ρ*(*x*_2_) ≥ *ρ*(*x*_3_) ≥ ... ≥ *ρ*(*x*_20_). If suppose *ρ* function does not satisfy the above condition, then reorder *G* so that *ρ* function must satisfy descending order condition and we will proceed further calculation based on the modified descending order condition. Let *μ*:*R*(*G*) → [0, 1] be a fuzzy measure. Then the fuzzy integral of *ρ* with respect to the fuzzy measure *μ* is given by

8 $$I=max[min{[\rho ({x}_{i}),\,\mu ({A}_{i})]}_{i=1}^{20}],$$

9 $${\rm{where}}\,{A}_{i}=\{{x}_{1},\,{x}_{2},\,\mathrm{...,}\,{x}_{i}\mathrm{\}.}$$

The fuzzy integral examine the fact supplied by each element of a given set, and the assessment of each subset of elements (using a fuzzy measure) in its decision-making process. The combination of the important significance of the source and the extracted information makes the fuzzy integral appropriate for information fusion. This theory has capability to tackle uncertainties associated with issue related to the processing procedures and data extraction. Therefore, this theory has been extensively applied in pattern recognition^31^ and classification.

### Fuzzy integral similarity and distance matrix for protein sequence comparison

The fuzzy integral similarity is based on the *h*^*th*^–step amino acids frequencies between the feature vector of the two sequences. Let *ν* and *τ* are feature vectors of the two sequences. We define fuzzy integral function *ρ*, which is given as:

10 $$\rho ({x}_{i})=1-|{x}_{i}^{\nu }-{x}_{i}^{\tau }|,$$

where *x*_*i*_ ∈ *G* (i.e., the similarity of the *h*^*th*^–step amino acid frequency *x*_*i*_ in the two feature vectors *ν* and *τ*).

Using fuzzy measure, we can determine the relative importance of subsets of amino acids being considered. Taking benefit of the *λ*–fuzzy measure properties described above, we can formulate *μ* using the fuzzy density of the individual element *μ*^*i*^.

In this case,

11 $${\mu }^{i}=max({x}_{i}^{\nu },\,{x}_{i}^{\tau }),$$

where *x*_*i*_ ∈ *G* (i.e., the maximum level of *h*^*th*^–step amino acid frequency starting from amino acid *b* and ending with amino acid *a*_*i*_ between two feature vectors with respect to their assigned position). Using equation (7), we calculated the value of *λ* and put the *λ* value in equation(6) to obtain the fuzzy measure *μ*. It can be easily verified that *μ* satisfy the properties (*i*) and (*ii*) of the fuzzy measure. Once we have *ρ* and *μ*, it is a straight forward using equation(8) to obtain the fuzzy integral^21^.

Next we calculate difference between two feature vectors *ν* and *τ*, which is given as follows:

12 $$D(\nu ,\,\tau )=1-I(\nu ,\,\tau ),$$

where *I*(*ν*, *τ*) is fuzzy integral similarity between *ν* and *τ*

The above process is continued for all pairwise combinations taken from *n* number of protein sequences. Finally, a distance matrix was generated. This distance matrix contained the dissimilarity information related to *n* protein sequences. This distance matrix was used as an input data to the neighbor.exe program in PHYLIP package^24^ for phylogenetic tree construction.

**Algorithm**

This section explains an algorithmic view of the developed method. The complete algorithm consists three stages.

**Stage 1:** Calculation of optimal-step transition probability matrix using Markov chain estimated from observed protein sequences:

**Algorithm 1:**
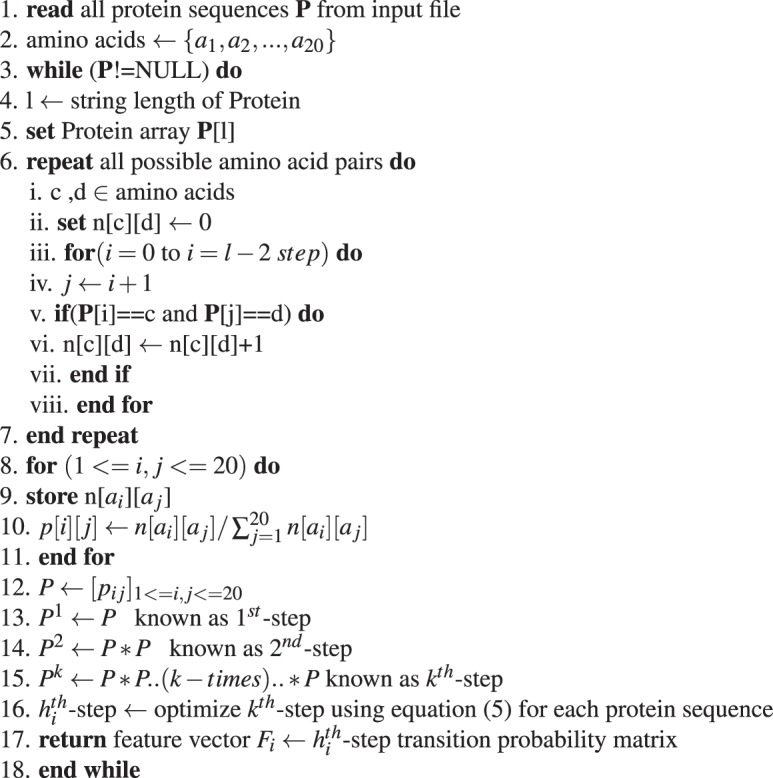
Derivation of *h*^*th*^-step transition probability matrix.

**Stage 2:** Fuzzy integral similarity between two feature vectors *F*_1_ and *F*_2_:

**Algorithm 2:**
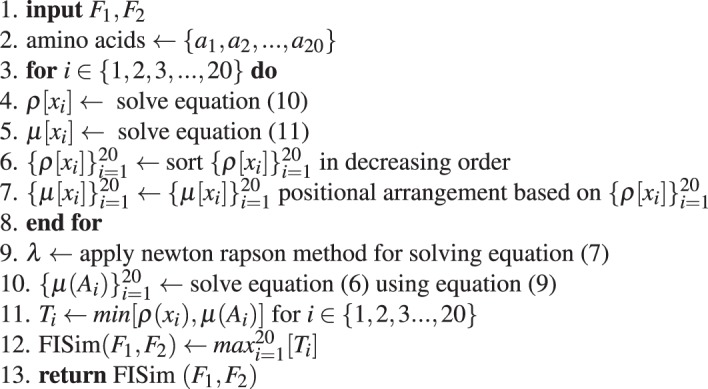
FISim (*F*_1_, *F*_2_).

**Stage 3:** Integrate stage(1) and stage(2) for phylogenetic tree construction:

**Algorithm 3:**
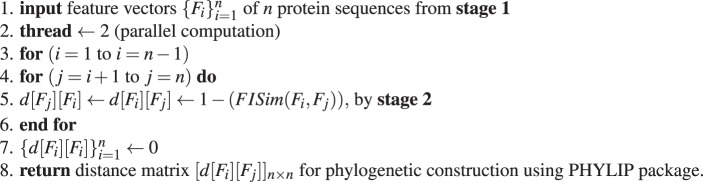
Distance matrix for phylogenetic tree construction.

### Time complexities of proposed algorithm

For calculating computational complexity^32^ of developed algorithm, we assumed that all operations took the same unit of time. Our algorithm was partitioned into three stages. For time complexity calculation: in the initial stage, transition probability matrices were calculated from the raw protein sequences. Time complexity of stage (1) is $O(nl+{m}^{3}{\sum }_{i=1}^{n}\,{h}_{i})$, where *n* is the total number of protein sequences, *m* is the number of amino acids, *l* is the average length of protein sequences and *h*_*i*_ is the optimal-step of feature vector. In the second stage, fuzzy integral similarity is calculated between two feature vectors. Therefore, time complexity of stage (2) is *O*(*m*2^*m*^). In the third stage, we integrated both the stages for generating distance matrix. Here, we used parallel computation for reducing the time complexity. Therefore, total time complexity for generating distance matrix is:

$$\begin{array}{ll}= & {\rm{time}}\,{\rm{complexity}}\,{\rm{of}}\,{\rm{stage}}\,1+((n(n-\mathrm{1))}/2t)\ast {\rm{time}}\,{\rm{complexity}}\,{\rm{of}}\,{\rm{stage}}\,2+{\varepsilon }_{t}\\ = & O(nl+{m}^{3}{\sum }_{i=1}^{n}\,{h}_{i})+((n(n-\mathrm{1))/2}t)\ast O(m{2}^{m})+{\varepsilon }_{t}\\ = & O(nl+{m}^{3}{\sum }_{i\mathrm{=1}}^{n}\,{h}_{i})+O(({n}^{2}m{2}^{m})/t)+{\varepsilon }_{t}\\ = & O({m}^{3}{\sum }_{i=1}^{n}\,{h}_{i}+nl+({n}^{2}m{2}^{m})/t)+{\varepsilon }_{t},\end{array}$$

where *t* is the number of threads and *ε*_*t*_ is the extra time taken in job assigning to all *t* threads. We also calculated the computational speed of our method and ClustalW method on tested datasets, which is given below in conclusion section.

## Results

To test our developed algorithm, we applied it to six sets of benchmark data. Different model might result different phylogenetic tree, therefore it is important to choose the most appropriate method. Here, we used Fitch-Margoliash or UPGMA (UPGMA = Unweighted Pair Group Method with Arithmetic Mean) approaches in PHYLIP package^24^ for generating the phylogenetic tree. On the benchmark data, result generated using both the approaches has minor differences between them. However, we chose optimal tree based on taxonomic classification and compare with existing tools. The six benchmark datasets used in this study are as follows:

(i) NADH Dehydrogenase 5 (ND 5) protein sequences.
(ii) NADH Dehydrogenase 6 (ND 6) protein sequences.
(iii) xylanases protein sequences in the F10 and G11 datasets.
(iv) transferrin protein sequences.
(v) coronavirus spike protein sequences.
(vi) beta-globin protein sequences.

### NADH Dehydrogenase 5 (ND 5) protein sequences

**Figure 1:**
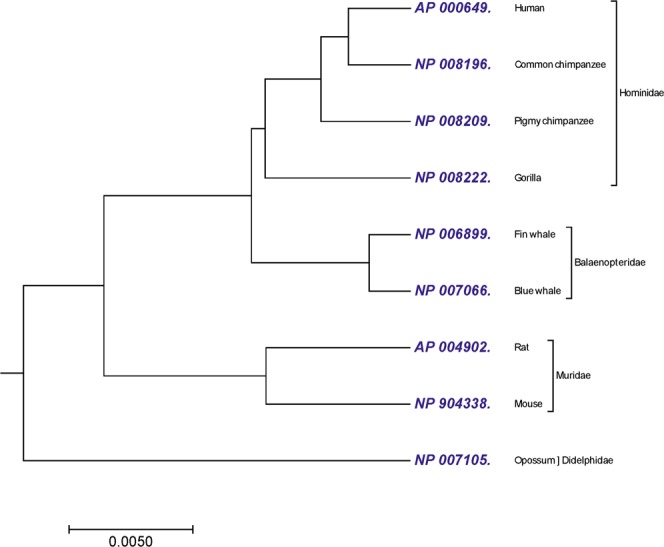
The phylogenetic tree of 9 sequences of NADH Dehydrogenase 5 protein constructed by our method using Fitch-Margoliash approach.

The proposed algorithm was tested on the benchmark dataset of 9 protein sequences of NADH Dehydrogenase 5 with nearly 600 amino acids (Table S1). All the sequences was obtained from the NCBI genome database. The MT-ND5 gene provides instructions for making a protein called NADH dehydrogenase 5. This protein is a part of a large enzyme complex known as complex I, which is active in mitochondria. Mitochondrially encoded NADH dehydrogenase 5 (complex I) in eukaryotes recognize as highly conserved subunit composition^33^. Therefore ND5 has been widely used for the analysis of the phylogenetic studies and their evolution. The phylogenetic tree generated by our method shown in Fig. 1, successfully grouped similar category based on taxonomic family classification. 9 sequences of ND5 protein belonged to mammals can be divided into following four categories based on their family; (i) *Hominidae* includes human, pigmy chimpanzee, common chimpanzee and gorilla; (ii) *Balaenopteridae* includes fin whale and blue whale; (iii) *Muridae* includes mouse and rat; and (iv) *Didelphidae* include opossum. From Fig. 1, it is clear that our method successfully clustered protein sequences separately based on their families. To illustrate the effectiveness of our method, we compared the phylogenetic tree generated by our approach with the phylogenetic tree generated by ClustalW using MEGA package^25^ (Fig. S1) and phylogenetic trees generated by the previous studies^13,34–38^ on the same dataset. Figure 1 generated by our method did not clustered common chimpanzee and pigmy chimpanzee together as compared to Fig. S1. However, tree generated by our approach (Fig. 1) has advantage over^37,38^. In^37^, phylogenetic trees construction based on the 20-D amino acid position ratio vector method and based on the 20-D amino acid content ratio vector method, four categories based on their family; *Hominidae*, *Balaenopteridae*, *Muridae* and *Didelphidae* are not separately clustred. Similarly in^38^ and^37^, phylogenetic trees construction based on the 20-D moment of inertia method and based on the 40-D amino acid position ratio and content ratio vector method, opossum is not separated as an outgroup.

We used correlation coefficient (CC) and Robinson-Foulds distance (RF-distance)^26^ as a statistical tools for comparative analysis between two phylogenetic trees. As a general perception more CC means higher similarity between an inferred tree and a reference tree. Similarly, we often use the RF-distance^39,40^ for analyzing topological similarity between two trees. RF-distance = 0 indicates that the test-tree topology is completely similar to that of the reference tree, while similarity level decreases as the RF-distance value increases. We obtained or calculated the CC and RF- distance of different alignment-free methods (Table 1) against the reference tree (ClustalW method). We used R-package for both CC and RF-distance calculation. In the Table 1, Jayanta *et al*.^34^ (with grouping) method shows that, even the CC is very high (0.9403) as compared to our method CC (0.7378) but their corresponding the RF-distance is 4, which is higher than our method RF-distance which is 2 (i.e., tree from^34^ (with grouping) is topologically less similar as compared to our tree to the reference tree). Similarly in Table 1, Wen *et al*.^35^ and Yao *et al*.^36^ having CC 0.7324 and 0.6908, respectively, which is nearer to CC of our method (CC = 0.7378). However, in terms of topological similarity, the RF- distance of Wen *et al*.^35^ and Yao *et al*.^36^ are 4 which is higher than RF-distance of our method. The above analysis shows that higher or closer CC does not always implies that the two phylogenetic trees are more similar or closer to each other.

**Table 1:** Comparison of alignment-free methods with the ClustalW based on correlation coefficient (CC) and Robinson-Foulds distance (RF-distance) on the ND 5 dataset.

Methods	Correlation coefficients	Robinson-Foulds distance (RF-distance)
Our method	0.7378	2
Jayanta *et al*. (without grouping)^34^ (Table 7*)	0.9734	0
Li *et al*.^37^ (Table 4*)	0.962	0
Jayanta *et al*. (with grouping)^34^ (Table 8*)	0.9403	4
Ma *et al*.^13^ (Table 3*)	0.9304	0
Wen *et al*.^35^ (Table 3*)	0.7324	4
Yao *et al*.^36^ (Table 3*)	0.6908	4
Czerniecka *et al*.^38^ (Table 8*)	0.61840	10

### NADH Dehydrogenase 6 (ND 6) protein sequences

**Figure 2:**
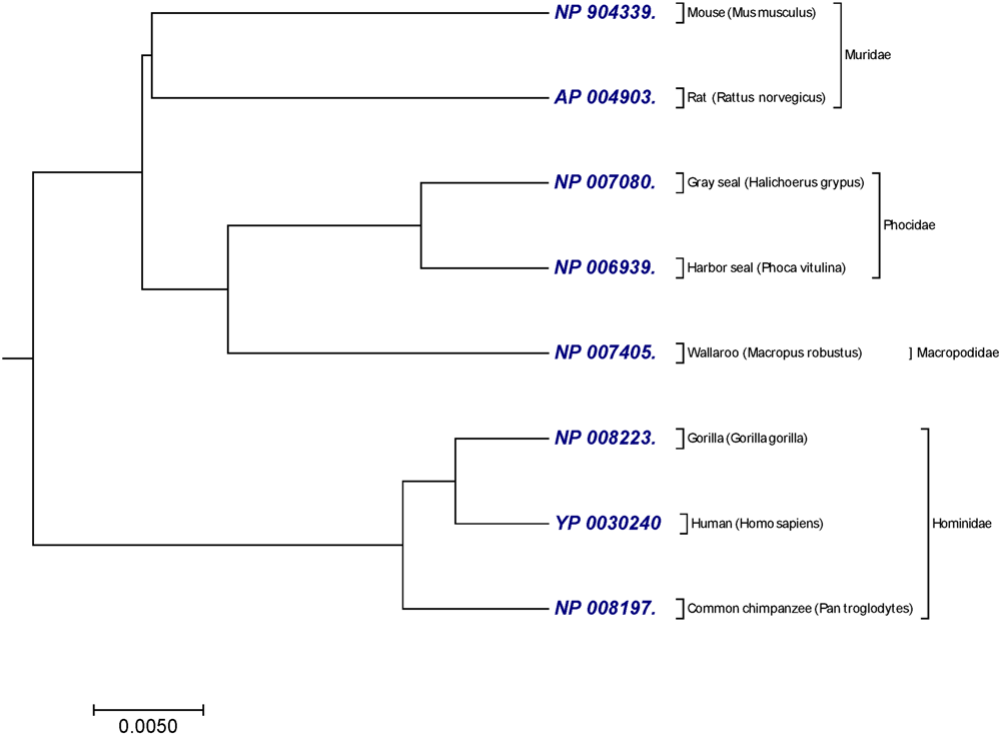
The phylogenetic tree of 8 sequences of NADH Dehydrogenase 6 protein constructed by our method using Fitch-Margoliash approach.

The other benchmark dataset used in this study was 8 protein sequences of NADH Dehydrogenase 6 with nearly 175 amino acids (Table S2). All the sequences were obtained from the NCBI genome database. NADH-ubiquinone oxidoreductase chain 6 is a protein that in human is encoded by the mitochondrial NADH Dehydrogenase 6 gene. The ND6 protein is a subunit of NADH dehydrogenase (ubiquinone), which is found in the mitochondrial inner membrane and is the biggest of the five complexes of the electron transport chain^41^. 8 sequences of ND6 protein belong to mammals can be divided into following four categories based on their taxonomic family; (i) *Hominidae* includes human, common chimpanzee and gorilla; (ii) *Phocidae* includes harbor seal and gray seal; (iii) *Muridae* includes mouse and rat; and (iv) *Macropodidae* include wallaroo. As shown in the tree generated by our method (Fig. 2), the protein sequences belong to the families *Hominidae*, *Muridae* and *Phocidae* were correctly separated. Based on the taxonomic family classification, we compared our tree with the trees genetated in the previous studies^38,42^ and tree generated by the ClustalW using MEGA package^25^ (Fig. S2). The tree generated by our method has an advantage over^38^, because it did not cluster (harbor seal, gray seal) and (mouse, rat) in separate clades. However, Fig. 2 shows consistency with^42^ and Fig. S2 based on taxonomic family division.

We calculated CC and RF-distance from previous studies^38,42^ with ClustalW. CC and RF-distance were also calculated between the our method and with ClustalW. In Table 2, Czerniecka *et al*.^38^ method has lower CC (0.4609) than CC (0.5982) generated by our method compared with ClustalW method, and their corresponding RF-distance (RF = 6) is much higher than our method (RF = 2). Therefore, phylogenetic tree generated by our method (Fig. 2) is more topologically similar than phylogenetic tree generated by Czerniecka *et al*.^38^ compared to reference tree (Fig. S2). However in Table 1, Gupta *et al*.^42^ method has higher CC (0.7763) as compared to our method CC (0.5982) but both the methods has the same RF-distance = 2.

**Table 2:** Comparison of alignment-free methods with the ClustalW based on correlation coefficient (CC) and Robinson-Foulds distance (RF-distance) on the ND 6 dataset.

Methods	Correlation coefficients	Robinson-Foulds distance (RF-distance)
Our method	0.5982	2
Gupta *et al*.^42^ (Table 2*)	0.7763	2
Czerniecka *et al*.^38^ (Table 13*)	0.4609	6

### Xylanases protein sequences in the F10 and G11 datasets

**Figure 3:**
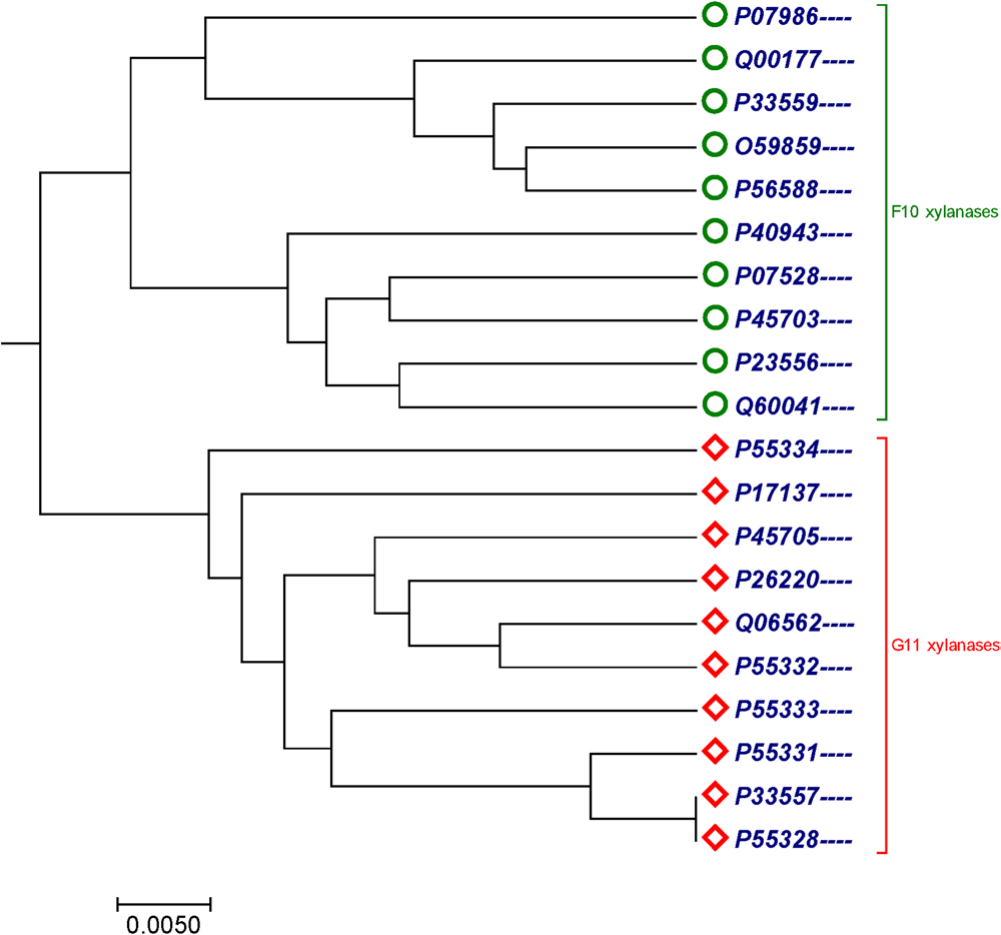
The Phylogenetic tree for 20 sequences of xylanases protein in the F10 and G11 datasets constructed by our method using Fitch-Margoliash approach.

The other benchmark dataset used for validation of the method was the 20 xylanases protein sequences in the F10 and G11 protein datasets with nearly 500 amino acids collected from^37^. Phylogenetic tree generated by our method (Fig. 3) accurately separated protein sequences belonging to G11 xylanases (red diomand) dataset from protein sequences belonging to F10 xylanases (green circle) dataset in separate branches. The phylogenetic tree generated in^37^ did not separated protein sequences belonging to family F10 and G11 in two separate branches. Figure 3 showed that there is an improvement in building phylogenetic tree with our method than the method used in study^37^. However, our tree (Fig. 3) is consistent with the tree generated by ClustalW using MEGA package^25^ (Fig. S3). We also calculated the CC and RF-distance between our method and ClustalW, which are 0.6998149 and 18.

### Transferrin protein sequences

**Figure 4:**
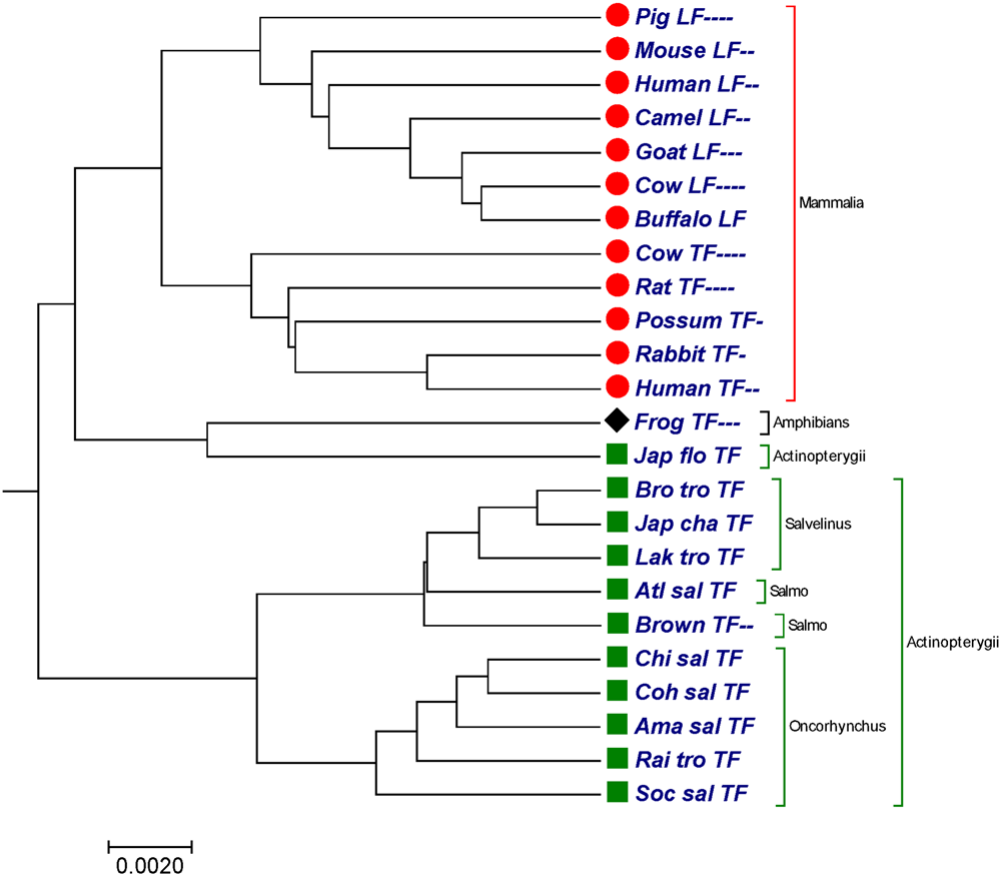
The phylogenetic tree for 24 sequences of transferrin protein constructed by our method using Fitch-Margoliash approach.

In this study the other benchmark dataset used was 24 protein sequence of transferrins (TFs) from vertebrates^43^ with nearly 700 amino acids (Table S3). All the sequences were obtained from the NCBI genome database. Transferrins are the iron-binding proteins that are involved in iron storage and resistance to bacterial disease. Transferrins have high binding affinities for iron and keep the free iron in low concentration in blood and other bodily fluids^44^. The phylogenetic trees constructed by our method (Fig. 4), successfully clustered transferrin protein sequences and lactoferrin protein sequences in separate clades. The tree generated by our approach (Fig. 4) divided the 24 sequences of transferrins (TFs) from vertebrates into three groups: *mammalia*(red circle), *actinopterygii*(green square) and *amphibians*(black diomand). Only Japanese flounder transferrin sequence belong to *actinopterygii* class was clustered with Frog transferrin sequence belong to *amphibians* class. In Fig. 4, sequences belong to genera *oncorhynchus* and *salvelinus* were clustered in separate clades, and sequences belong to genus *salmo* were placed close to each other.

Based on taxonomic division, comparison between alignment-free methods, the phylogenetic tree generated by our approach (Fig. 4) with phylogenetic tree generated in the previous studies^45,46^ indicates improvement in our approach. In Fig. 4, sequences belong to *mammalia* class were clustered in a separate clade which were not observed in^45,46^. Moreover, species belong to genera *oncorhynchus* and *salvelinus* were grouped into separate clades, which is lacking in^46^. While comparing our tree (Fig. 4) with the benchmark tree constructed by^43^ and tree constructed by ClustalW using MEGA package^25^ (Fig. S4), we noticed that they are consistent among each other. The calculated the CC and RF-distance between our method and ClustalW are 0.7453224 and 20.

### Coronavirus spike protein sequences

**Figure 5:**
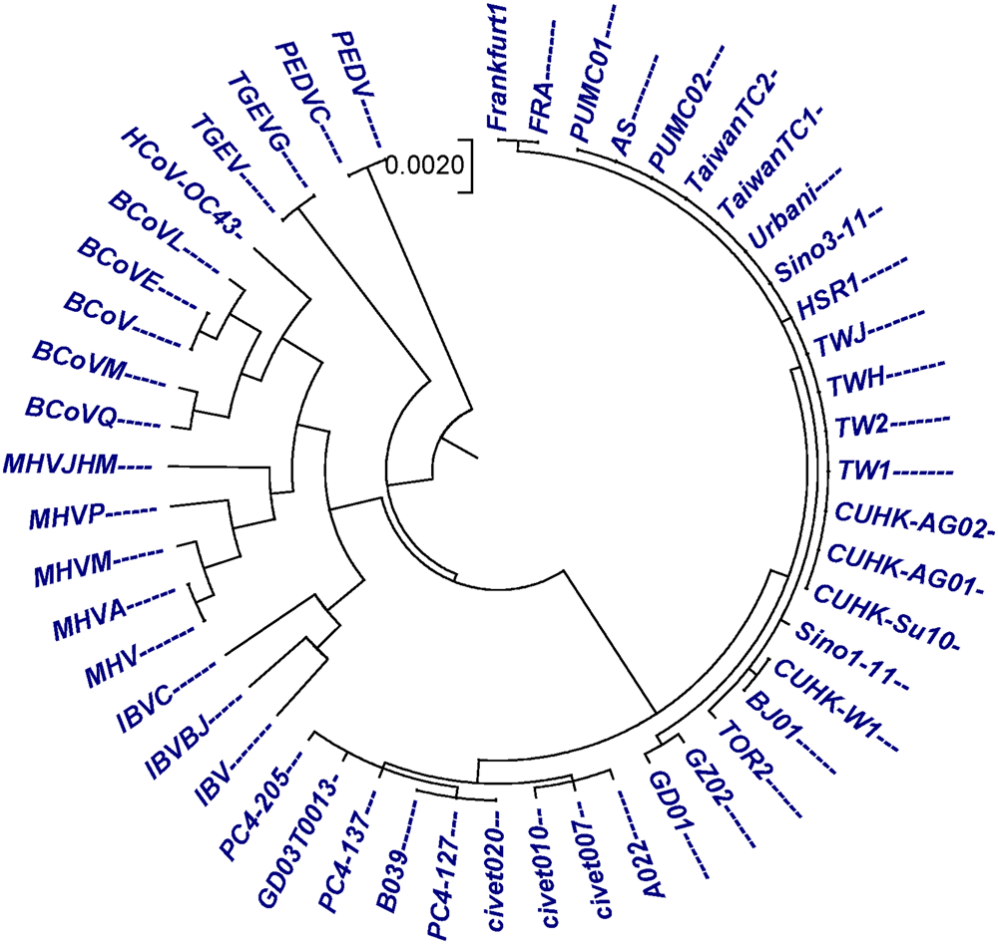
The phylogenetic tree for 50 sequences of coronavirus spike protein constructed by our method using Fitch-Margoliash approach.

The other benchmark dataset used for the validation of our method was the 50 coronavirus spike proteins (Table S4) with nearly 1500 amino acids. Coronaviruses are diverse group of large, enveloped, positive-stranded RNA viruses belonging to the family Coronaviridae. Coronaviruses are responsible for respiratory and enteric diseases in human and other animals. According to the host type, Coronaviruses can be divided into four groups (Table S4). Group I and II contains mammalian coronaviruses, group III contain avian coronaviruses and group IV contain SARS-CoVs^47–49^. The spike protein which is common to all known coronaviruses, is crucial for viral attachment and entry into the host cell. To illustrate the use of the quantitative characterization of these sequences, we employed our method to analyse the 50 coronavirus spike proteins. Observing Fig. 5, we found that SARS-CoVs (group IV) appear to cluster together and formed a separate branch, which can be easily distinguishable from other three groups(I, II and III) of coronaviruses. Similarly, sequences belonging to groups II and III are placed at an independent branch. While sequences belong to group I, such as (TGEV, TGEVG) and (PEDVC, PEDV) formed separate clades, but they were close to each other. A closer look at the subtree of SARS-CoVs (group IVa) belonged to 03–04 interspecies epidemic are cluster together, while all the human SARS-CoVs formed another branch. Phylogenetic tree generated by our method (Fig. 5) is consistent with phylogenetic trees generated in the previous studies^42,50,51^ and alignment based method ClustalW using MEGA package^25^ (Fig. S5). The CC and RF-distance between our method and the ClustalW are 0.9555357 and 46.

### Beta-globin protein sequences

**Figure 6:**
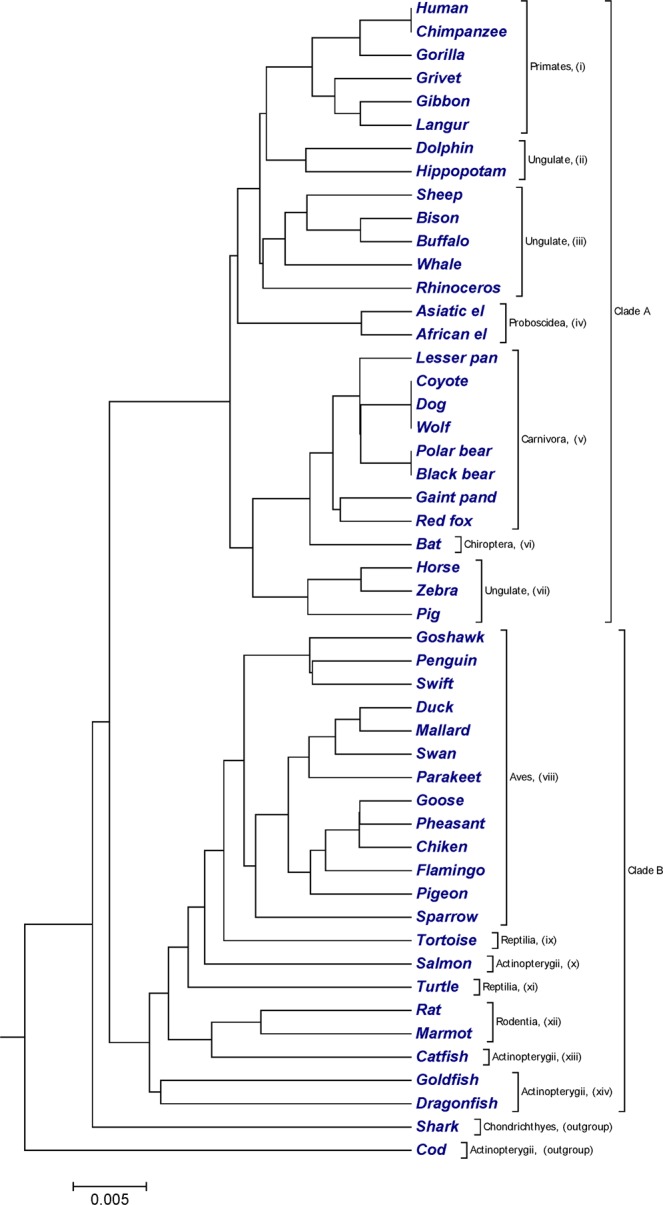
The phylogenetic tree for 50 sequences of beta-globin protein constructed by our method using UPGMA approach.

50 sequences of beta-globin protein (Table S5) of different species^52^ with nearly 150 amino acids were extracted from GenBank. Based on the type of host, 50 sequences of beta-globin protein can be classified into following groups such as primates, proboscidea, ungulate, carnivora, rodentia, chiroptera, aves, actinoptergii, reptilia and chondrichthyes. The phylogenetic trees constructed by our method (Fig. 6) separated 50 sequences of beta-globin protein into two major clades: clade A and clade B. Clade A contained mammalian beta-globins and clade B contained beta-globins from avian, fish, and reptilian species. According to the taxonomy division, we categorized two major clades into several sub-clades. All primates, proboscidea, carnivora, chiroptera, aves and rodentia were successfully cluster into clades (i), (iv), (v), (vi), (viii) and (xii) respectively. Ungulate were clustered into clades (ii), (iii) and (vii). We observed an obvious limitation in Fig. 6 is that, our approach failed to cluster fish species into single clades based on taxonomy. However, the phylogenetic tree generated by our approach is consistent and generated a better result based on taxonomic characteristic of species while compared with previous studies^45,53^. Phylogenetic tree generated by ClustalW using MEGA package^25^ (Fig. S6), successfully clustered fish species and reptilian species in separate clades, while our approach (Fig. 6) failed to cluster separately. However, from both figures, it is clear that phylogenetic tree generated by our method (Fig. 6) depicted more clear division in terms of branch length than phylogenetic tree generated by ClustalW (Fig. S6). The calculated CC and RF-distance between our method and ClustalW are 0.7294663 and 64.

## Conclusion

This study focused on fuzzy integral similarity method based on Markov chain and applied this algorithm to protein sequence analysis. Sequence comparison is the fundamental and most frequent activity in bioinformatics. In sequence alignment method, two sequences are assigned an alignment score based on insertion, deletion and substitution of nucleotides or amino acids. However, sometimes alignment becomes misleading due to unequal length of sequences, gene rearrangements, inversion, transposition and translocation at substring level. In these scenarios, alignment-free methods are therefore a better alternative as it reduces the technical constraints of alignments. We have constructed transition probability matrix using Markov chain of each protein sequence. Subsequently, a fuzzy integral similarity method was used to assign similarity score belong to closed interval [0, 1] between two protein sequences. The benefit of our approach is that, it do not require any prior biological knowledge regarding homologous relationship (common ancestry) among the sequences which makes it fully automated and robust. We implemented our method on six benchmark datasets as discussed in the result section.

In Figs 1 and 2, our method successfully grouped NADH Dehydrogenase 5 and NADH Dehydrogenase 6 protein sequences into four categories based on the taxonomic family classification. However, in Fig. 1, common chimpanzee is closer to human than pigmy chimpanzee, which is contrast to the known fact of evolution. In xylanases protein sequences, tree generated by our approach (Fig. 3) correctly distinguished 20 sequences of xylanases protein belong to families G11 and F10 in separate clades. Similarly in Fig. 4, it is clear that, our method separated the transferrin protein sequences and the lactoferrin protein sequences into separate clades, which is desirable. A satisfactory improvement can be seen in the phylogenetic tree built by our algorithm at genus level (Fig. 4). Our tree (Fig. 4) successfully separated sequences belong to genera *oncorhynchus* and *salvelinus* in separate branches, and sequences belong to genus *salmo* were closest to each other. In coronavirus spike protein, phylogenetic tree generated by our approach (Fig. 5) nicely categorized four groups based on their host types (groups I, II, III and IV). Moreover, our method successfully categorized SARS-CoVs which belong to group IV into two subgroups, which corresponds to the 03–04 interspecies epidemic and human epidemic, respectively. Finally, we implemented our method on 50 sequences of beta-globin protein. An obvious default in Fig. 6 generated by our method is that our approach failed to cluster fish species into a single clade. However, we found consistency while comparing our tree (Fig. 6) with recently developed alignment-free method collected from^45,53^.

Our programs were executed on a linux server with 24 dual core processor with 384 GB RAM. We enriched our programs by incorporating parallel computation, which can reduce the execution time of our program by increasing the number of threads, depending on the number of sequences. In our program, we implemented two threads as a default parameter. However, the user can manipulate the parameter to single thread or multiple threads. The execution time of our method with two threads is shown in Table 3. In the Table 3, the execution time of our method for 50 sequences of coronavirus spike protein is 16 seconds by using two threads, which can be reduced to 7 seconds and 5 seconds by using threads four and six, respectively. In this study, we implemented statistical tools such as CC, RF-distance and ROC^27–29^ curve to compare the result generated by our method with the other alignment-free methods. We performed comparative study between the RF-distance and the CC for each method for the ND5 and ND6 datasets. Similarly, we plotted ROC curve and calculate area under the ROC curve (AUC) for distance matrices generated by our method and other alignment-free tools from Alfree repository^54^. The results of ROC and AUC analysis for all benchmark datasets are given in supplementary material. We are yet to attain an highly efficient alignment-free method for phylogenetic analysis. However, our method shows an improvement over the other existing alignment-free methods in terms of sequence clustering. Based on the observed progress, this method would be useful for the researcher to develop hypothesis that can be examined further in details. Before continuing our research work for further improvement, we would like to emphasize that this is a probabilistic approach in nature. It can later be modified by including more biological evidence. Overall, our goal in this study was to bring a new methodology or algorithm to the proteomics study. This proposed algorithm can be used to guide the development of more powerful measures for sequence analysis.

**Table 3:** Running time of our method.

Datasets	ND 5	ND 6	xylanases	transferrin	Coronavirus	beta-globin
Amino acids (approximate lengths)	600	175	500	700	1500	150
Number of sequences	9	8	20	24	50	50
Our method (execution time)	1 s	1 s	3 s	4 s	16 s	15 s

## Supplementary information

Dataset 1

Dataset 2

ROC_supplementary material

## Data Availability

We wrote code in C-programming which is available via our institute website.
