# Supplementary material for: Alignment-free similarity analysis for protein sequences based on fuzzy integral
Source: Sci Rep. 2019 Feb 26;9:2775. doi: 10.1038/s41598-019-39477-8 (PMC6391537; doi:10.1038/s41598-019-39477-8)
Supplement: Supplementary file 1 — Dataset 1 [file 41598_2019_39477_MOESM1_ESM.pdf]

Alignment-free similarity analysis for protein sequences based on fuzzy integral.

Ajay Kumar Saw[1], Binod Chandra Tripathy[2], and Soumyadeep Nandi[3*]

[1]Institute of Advanced Study in Science and Technology, Mathematical Sciences Division, Guwahati-781035, India
[2]Tripura University, Department of Mathematics, Agartala - 799022, India
[3]Institute of Advanced Study in Science and Technology, Life Science Division, Guwahati-781035, India

[*]Corresponding author: soumyadeep.nandi@gmail.com

ROC_SUPPLEMENTARY MATERIALS

Explanation:

The goal of the study is to build an Alignment-free tool for sequence clustering. To demonstrate the efficiency of our method we compared the phylogenetic trees and the distance matrices generated by our method with the other freely available recent methods[1]. The superiority of the clustering performance of our method can be noticed in the following trees. We can see the superiority of efficient clustering of the sequences across all the benchmark datasets as compared to the other methods.

The consistency can also be observed from the statistical measurement such as AUC (area under the ROC) values calculated from ROC (Receiver operating characteristic) curves. AUC values are often used to describe the accuracy of models and not to compare them. Accuracy classification of AUC is summarised in Table 1, which is given below.

**AUC Range**	**Classification**
AUC ≥ 0.9	high accuracy
0.7 ≤ AUC< 0.9	moderate accuracy
0.5 ≤ AUC< 0.7	Low accuracy

Table 1: Accuracy classification of AUC [2,3,4,5,6].

A low AUC value does not necessarily suggest a bad or poor model; rather it simply suggests that, besides the accounted predictors, other factors also exercise influence on the response variable. It is evident from the ROC plots below that our method's performance is in good agreement with other methods.

In summary, our method performs similarly in terms of statistical measurements (ROC and AUC) with the other methods. However, the phylogenetic trees generated by our method and the other freely available recent methods [1] shows superiority of our method over the other methods in terms of sequence clustering.

ROC calculation:-

The phylogenetic tree is generated by a distance matrix which is equal to (1-similarity matrix), equation (12) in our manuscript. We took similarity matrix for ROC analysis. In similarity matrix, we assigned positive label, if the two sequences belong to same class (i.e., family level classification, phylum level classification, genus level classification, etc.,) otherwise assigned negative label. We generated the ROC curve by varying decision threshold between the minimum and maximum values of the similarity matrix and plotted the FPR (false positive rate) on X-axis and TPR (true positive rate) on Y-axis. Each point in the ROC curve corresponds to a discrete classifier that can be obtained using a given decision threshold (Figure 3 in [7]). After plotting ROC curve, we calculated the area under the ROC curve (AUC).

Phylogenetic tree on NADH Dehydrogenase 5 (ND 5) protein sequences.

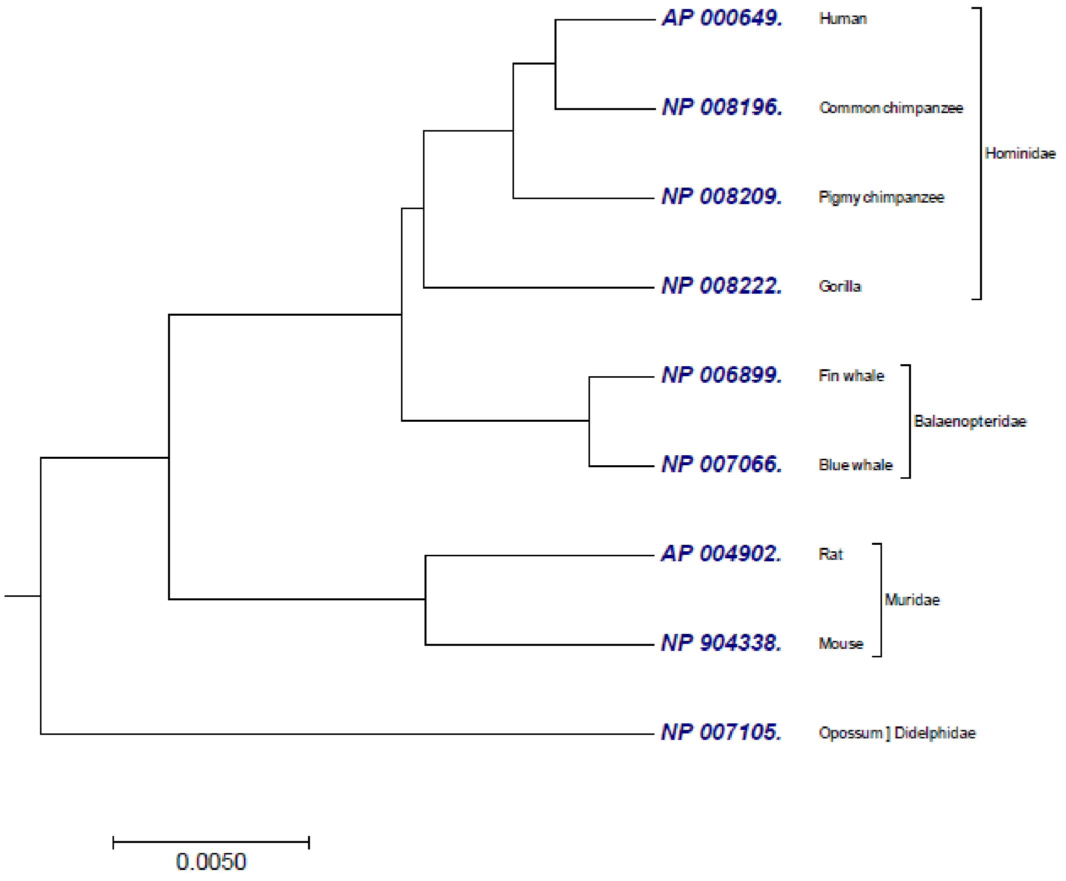

Figure 1: Our method.

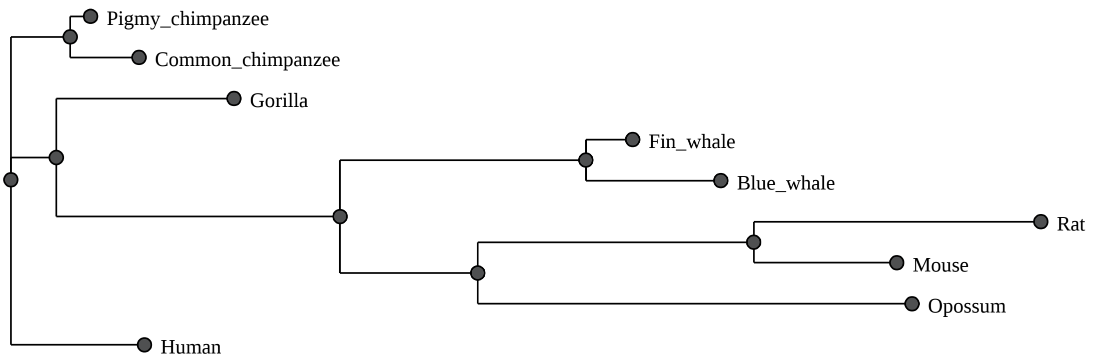

Figure 2: Feature Frequency Profiles (FFP) method.

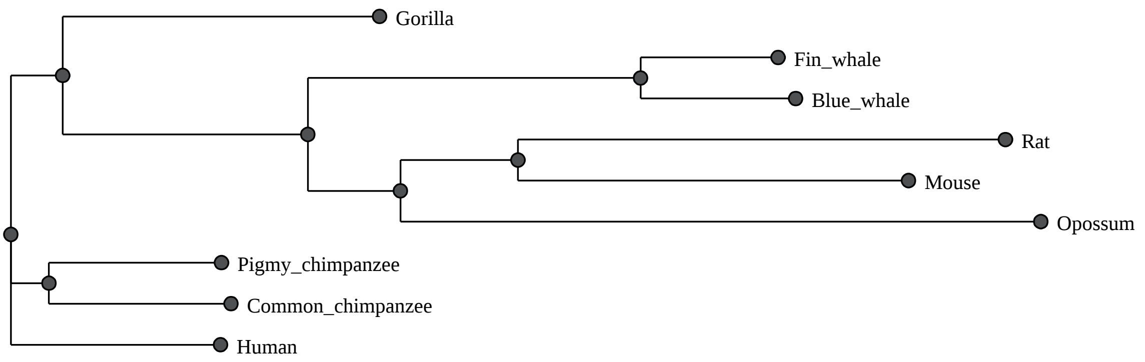

Figure 3: Return Time Distribution (RTD) method.

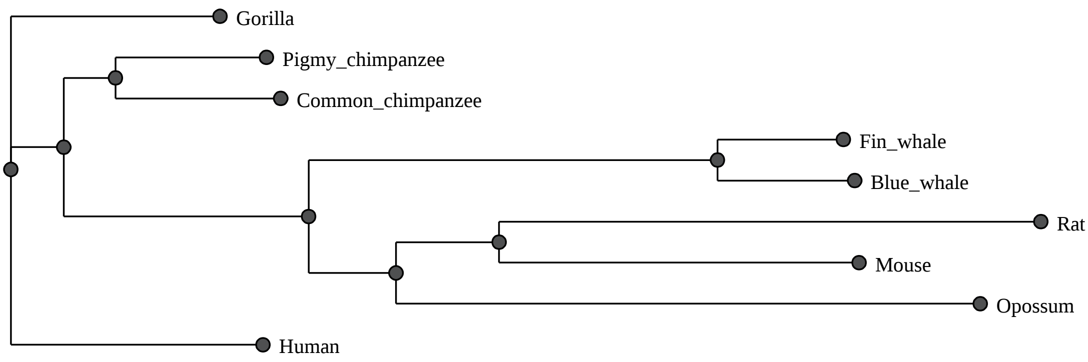

Figure 4: Composition distance (CV) method.

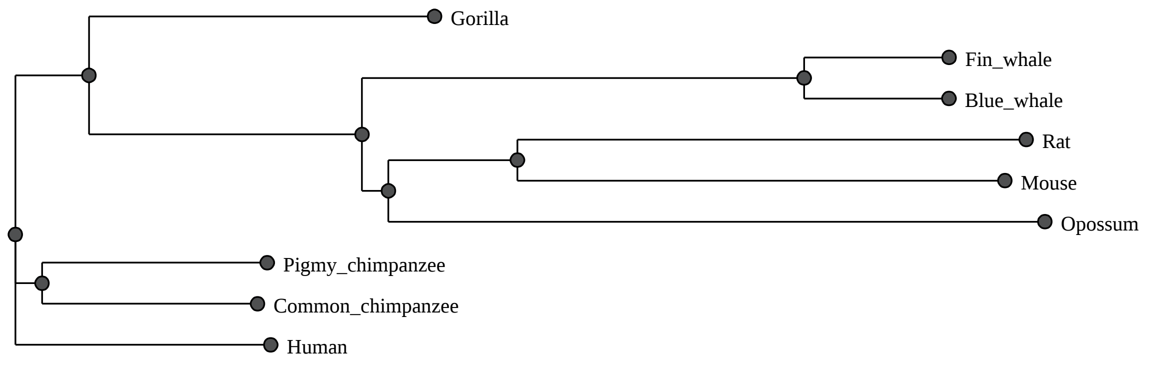

Figure 5: Normalized Compression Distance (NCD) method.

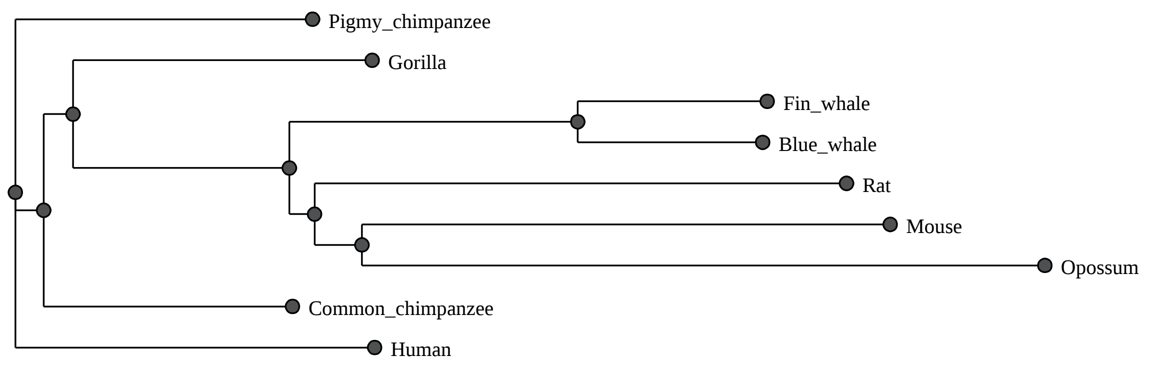

Figure 6: Base-Base Correlation (BBC) method.

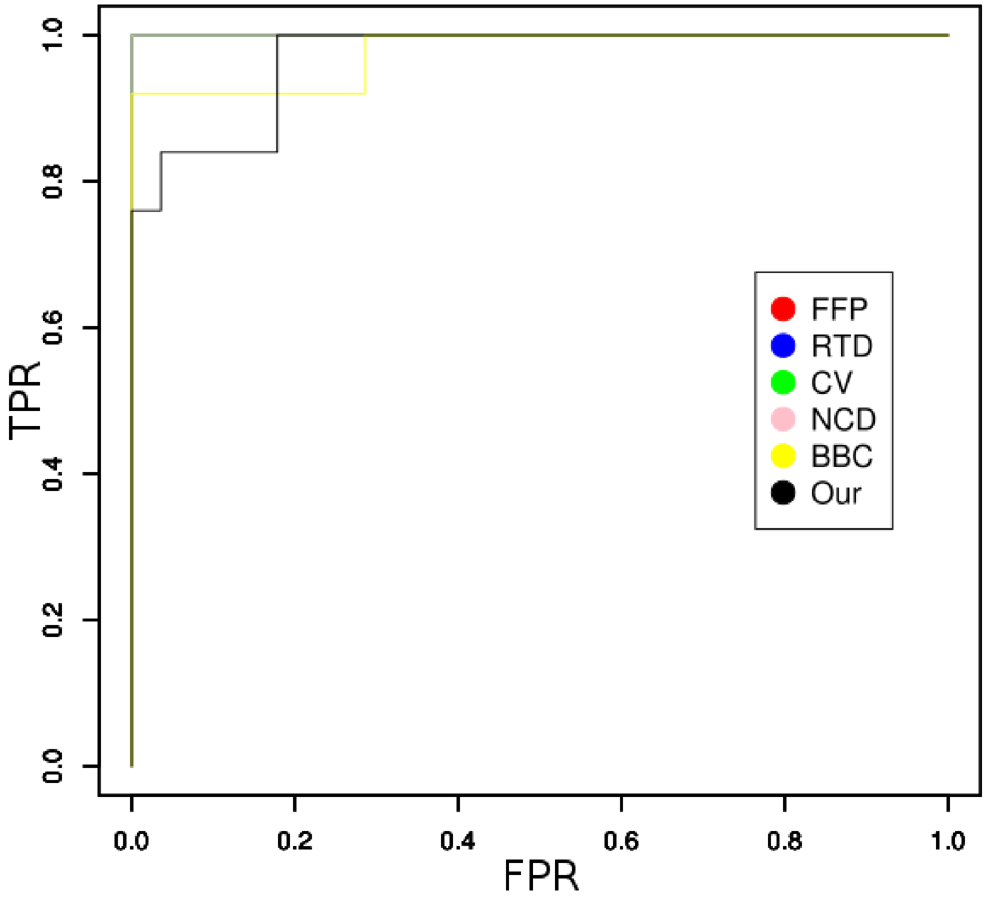

**Datasets ➡**	**ND5**
**Methods ⬇**	AUC⬇
FFP	1.00
RTD	1.00
CV	1.00
NCD	1.00
BBC	0.98
OUR	**0.97**

Figure 7: Receiver operating characteristic curve (ROC) and Area Under the ROC Curve (AUC) of ND5 dataset using different method.

The phylogenetic tree (Figure 1) generated by our method successfully clustered sequences belonged to family *Hominidae* which includes human, pigmy chimpanzee, common chimpanzee and gorilla in one clades and outgrouped opossum, which is missing in phylogenetic trees generated by other freely available tools[1] (Figures 2,3,4,5 and 6). This shows the advantage of our method over others in terms of sequence clustering. Moreover, the AUC of our method is 0.97 (Figure 7), which indicates that our method has high accuracy (Table 1) and approximately same with other AUC's.

Phylogenetic tree on NADH Dehydrogenase 6 (ND 6) protein sequences.

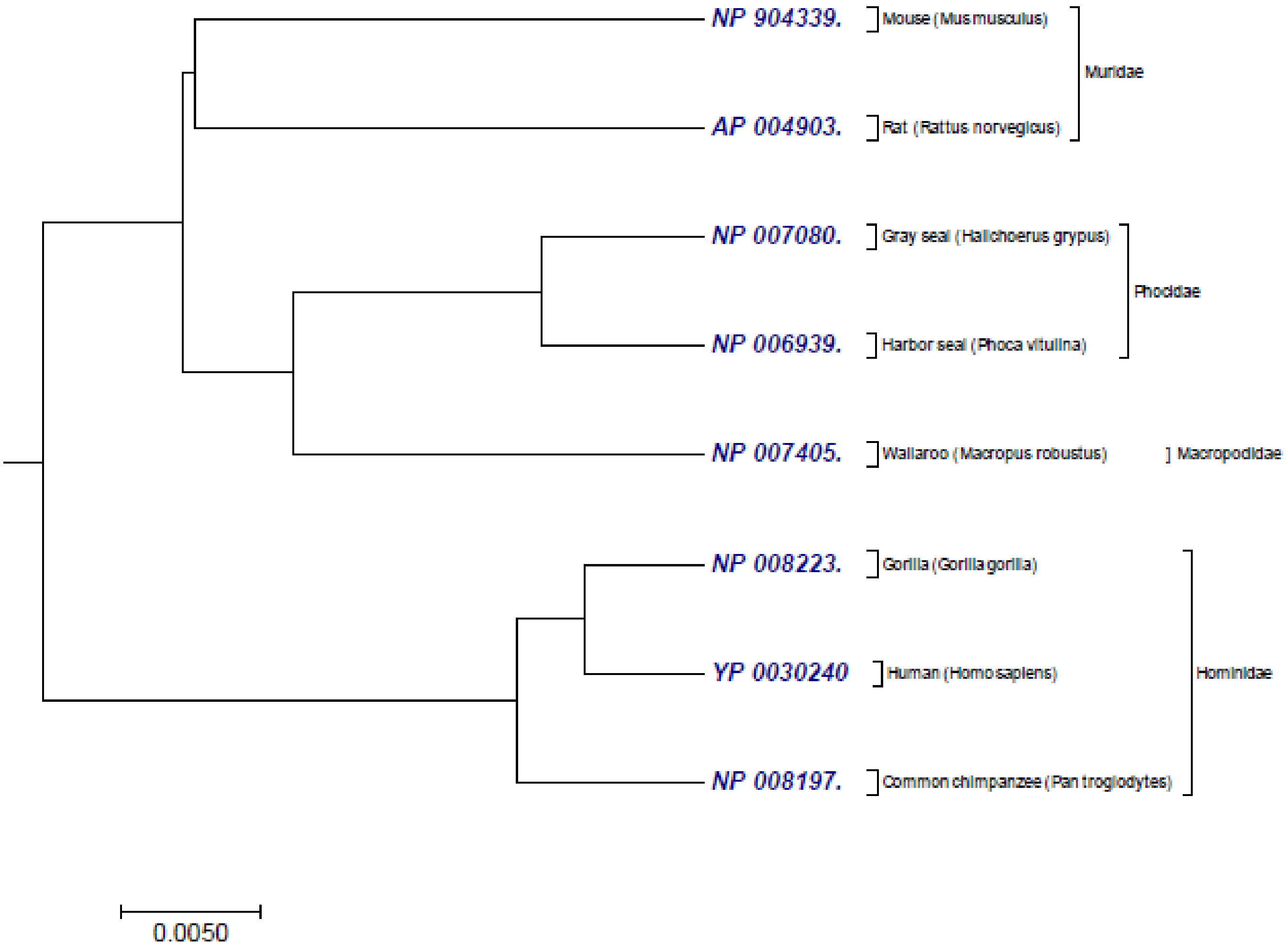

Figure 8: Our method.

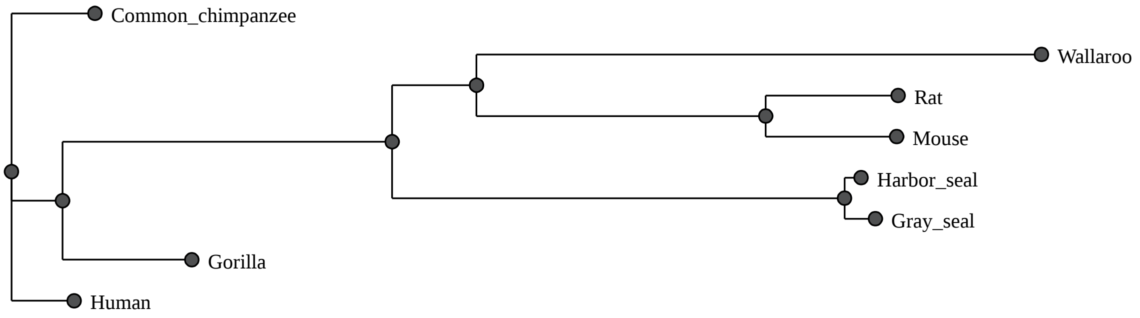

Figure 9: Feature Frequency Profiles (FFP) method.

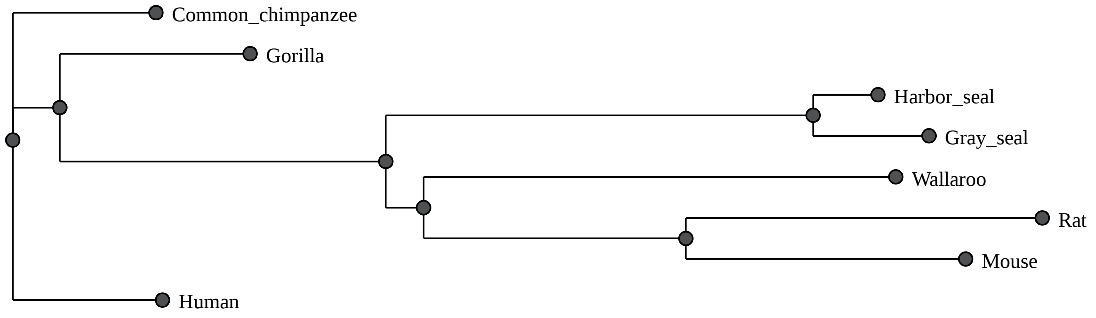

Figure 10: Return Time Distribution (RTD) method.

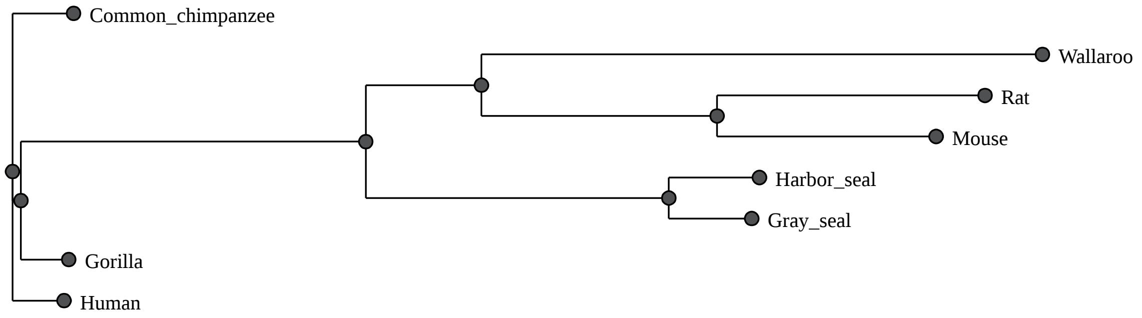

Figure 11: Composition distance (CV) method.

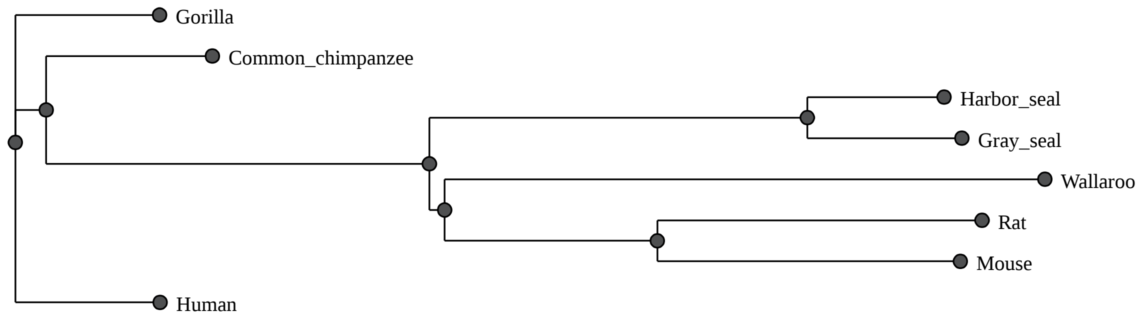

Figure 12: Normalized Compression Distance (NCD) method.

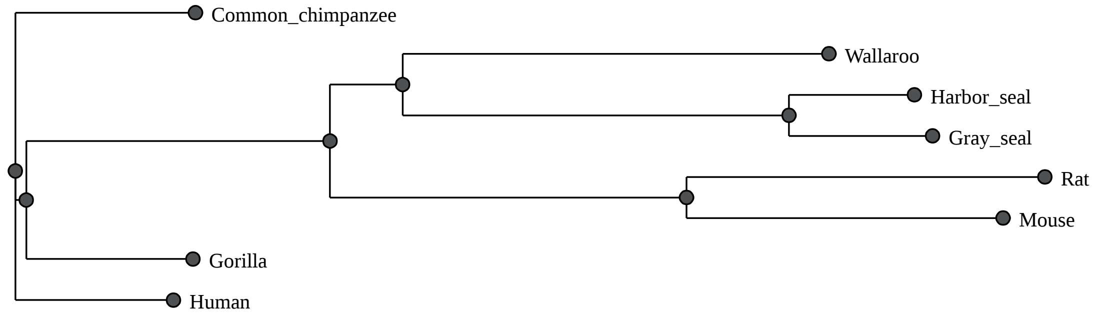

Figure 13: Base-Base Correlation (BBC) method.

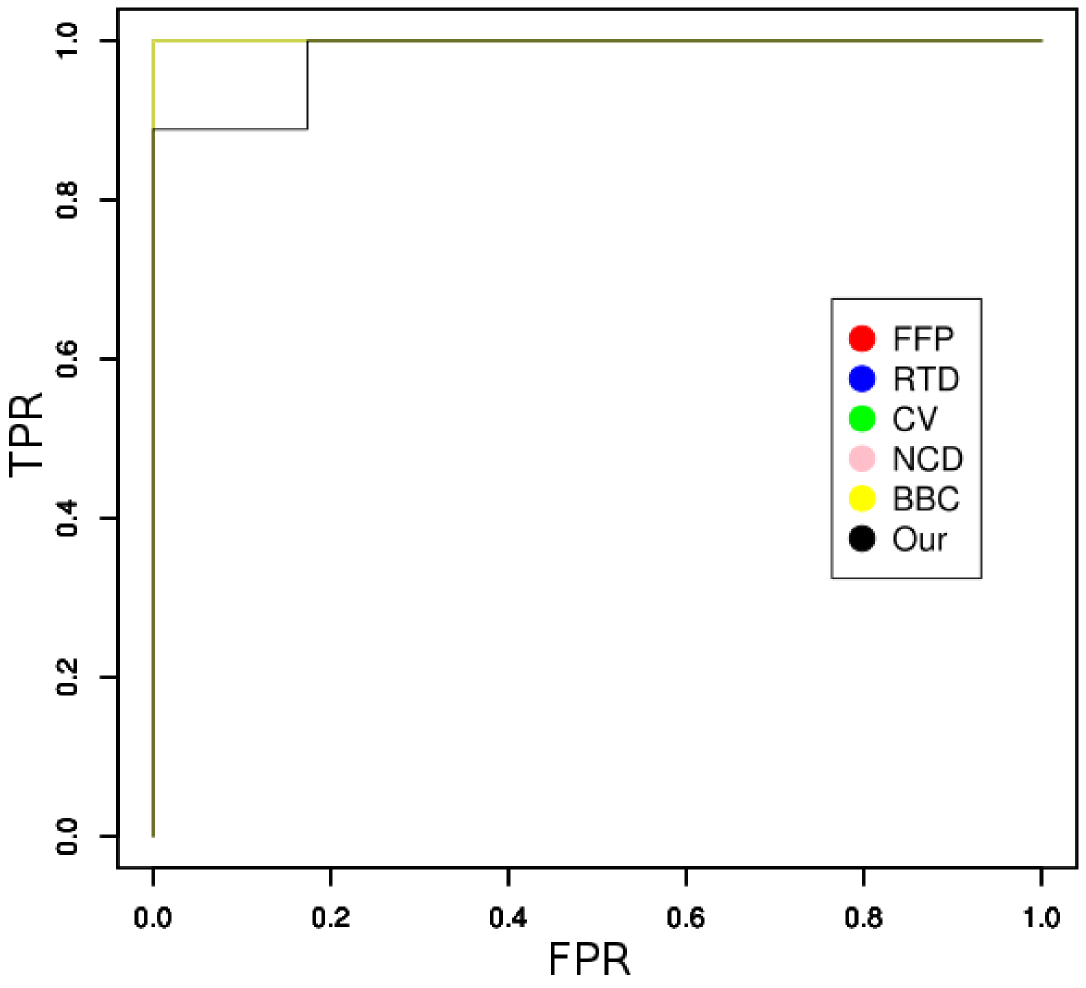

Datasets ➡	ND6
**Methods ⬇**	AUC⬇
FFP	1.00
RTD	1.00
CV	1.00
NCD	1.00
BBC	1.00
OUR	**0.98**

Figure 14: Receiver operating characteristic curve (ROC) and Area Under the ROC Curve (AUC) of ND6 dataset using different method.

The phylogenetic tree (Figure 8) generated by our method successfully clustered sequences belonging to family *Hominidae* which includes human, common chimpanzee and gorilla in one clades, which is missing in phylogenetic trees generated by other freely available tools[1] (Figures 9,10,11,12 and 13), which again shows the advantage of our method over others in terms of clustering. The AUC of our method is 0.98 (Figure 14), which indicates that our method has high accuracy (Table 1) and approximately same with other AUC's.

Phylogenetic tree on 50 Coronavirus spike protein sequences.

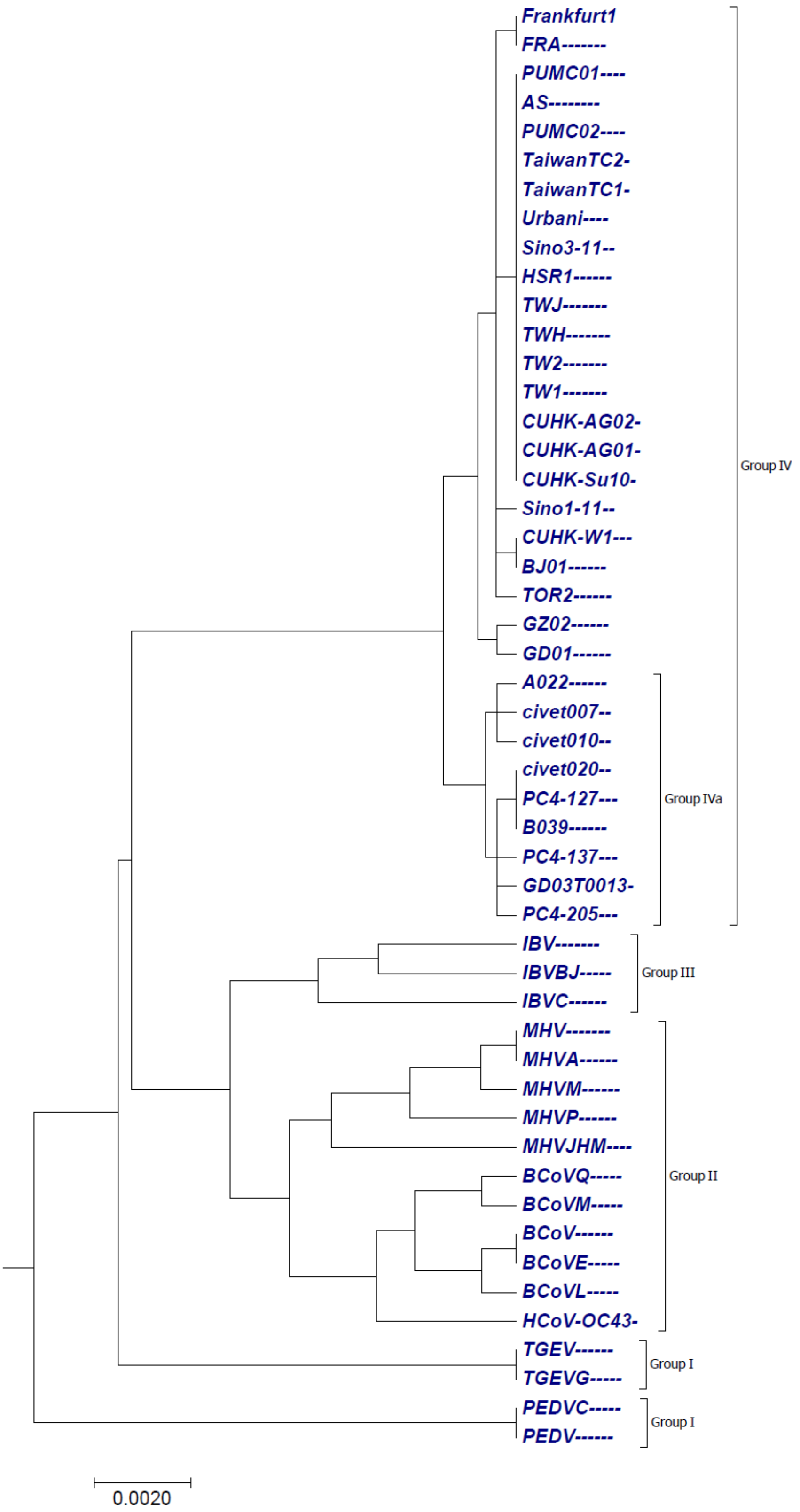

Figure 15: Our Method.

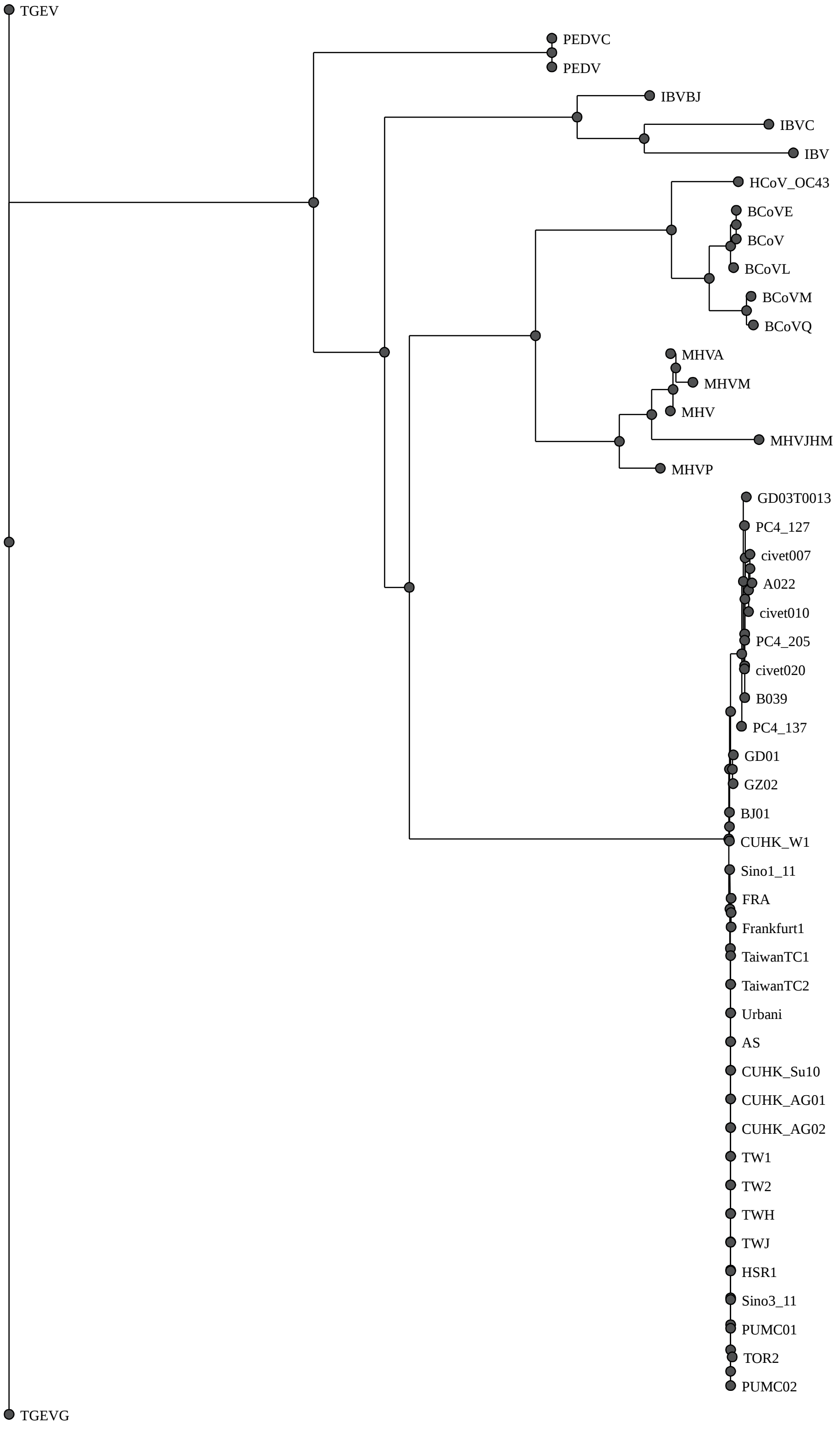

Figure 16: Feature Frequency Profiles (FFP) method.

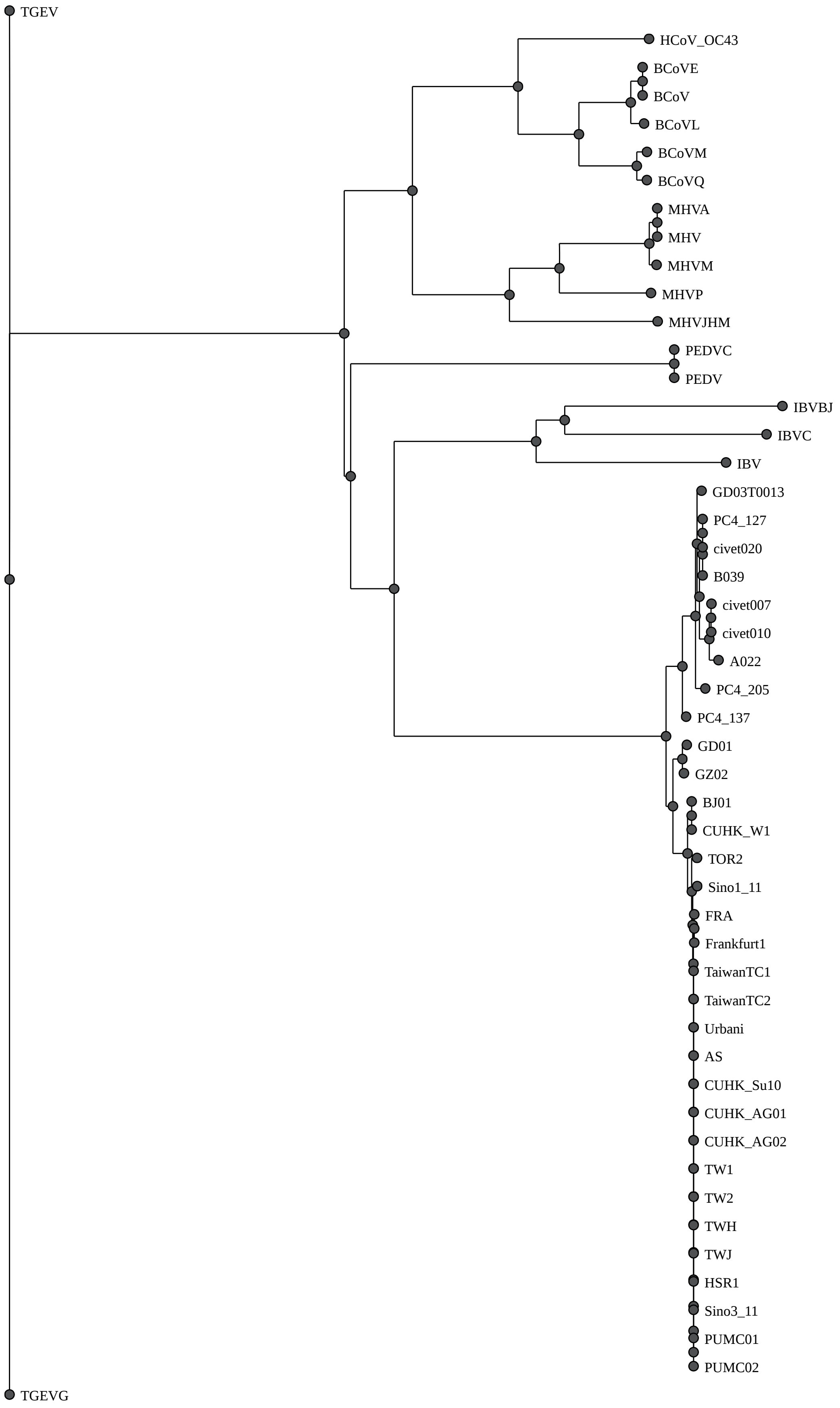

Figure 17: Return Time Distribution (RTD) method.

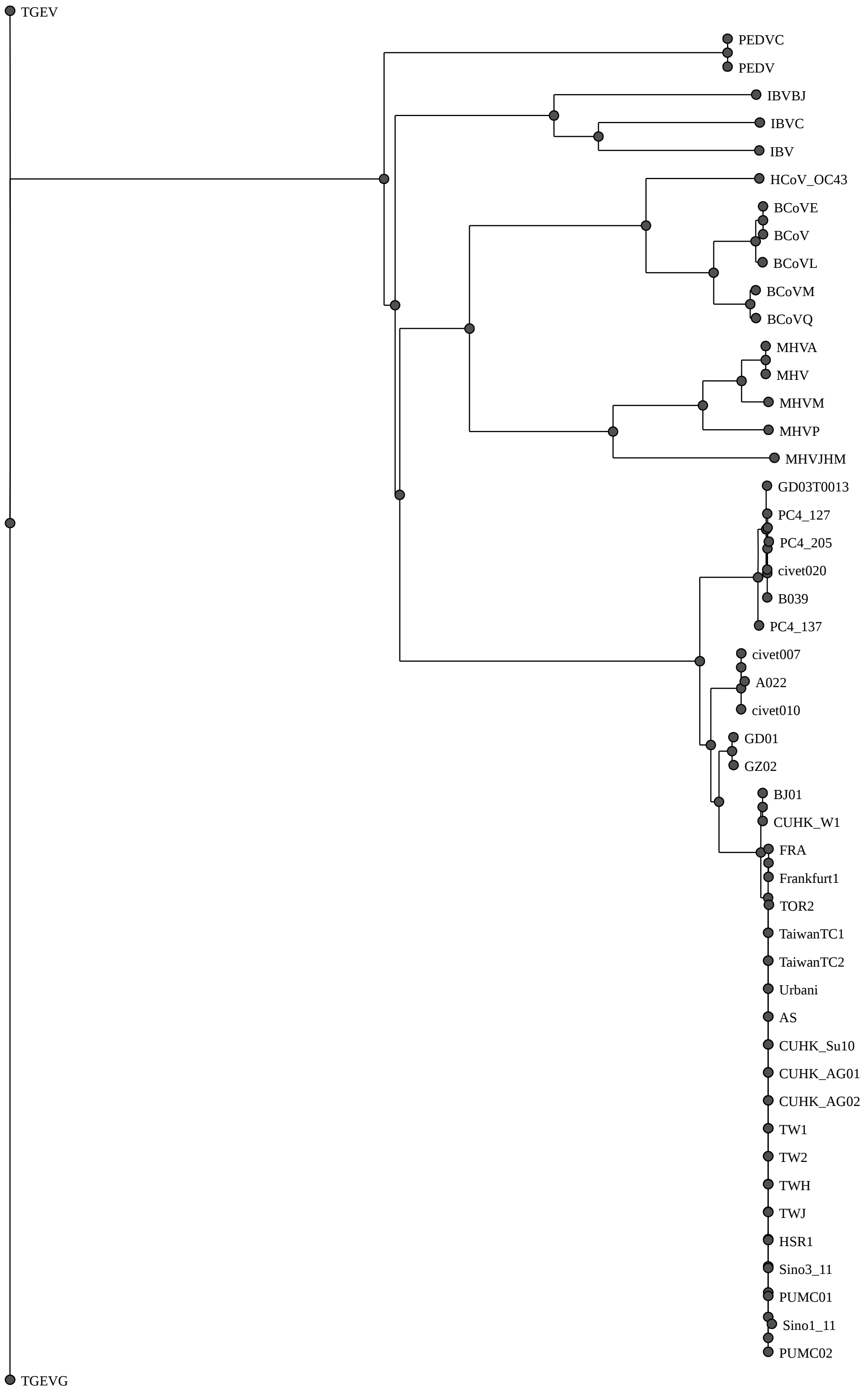

Figure 18: Composition distance (CV) method.

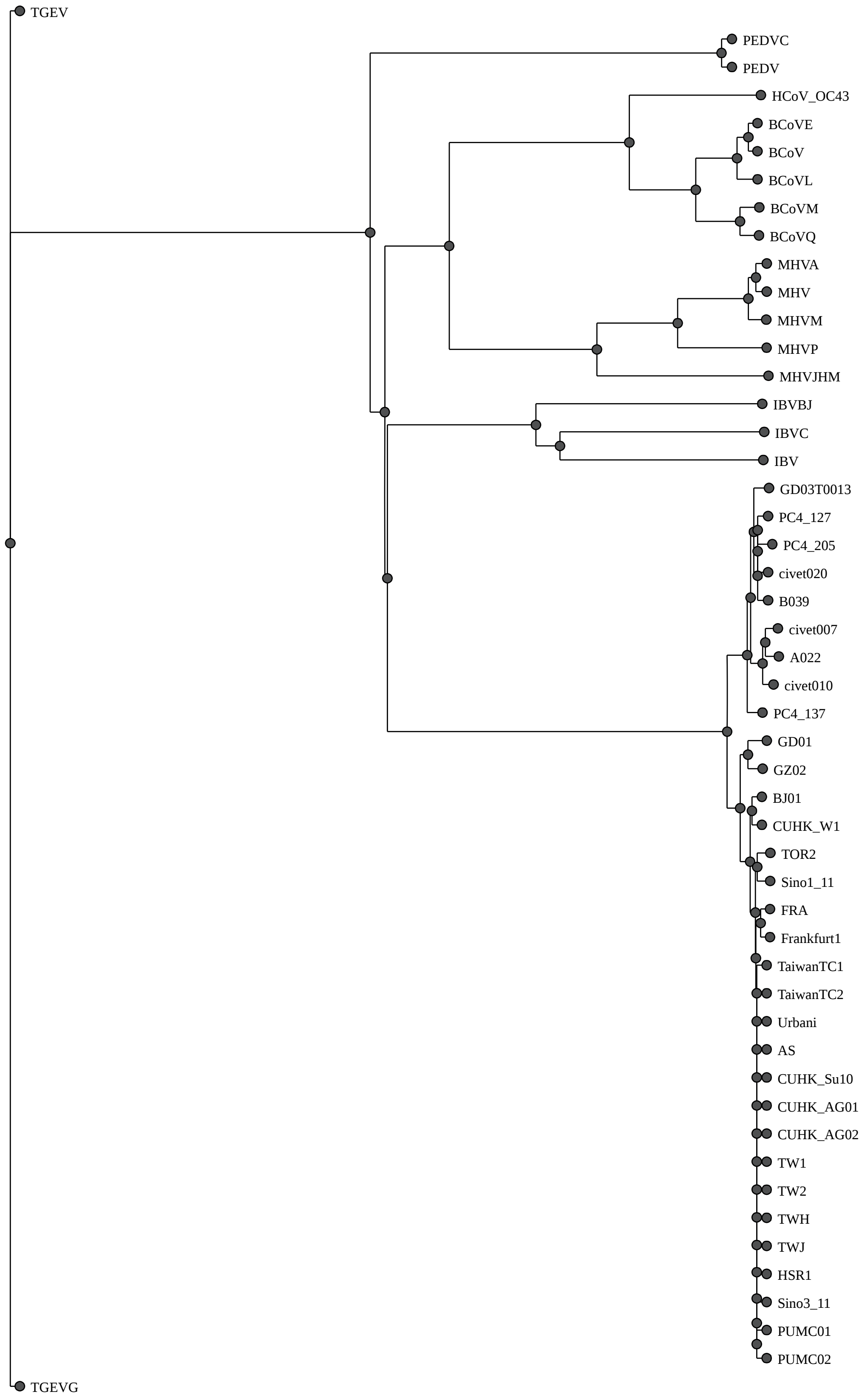

Figure 19: Normalized Compression Distance (NCD) method.

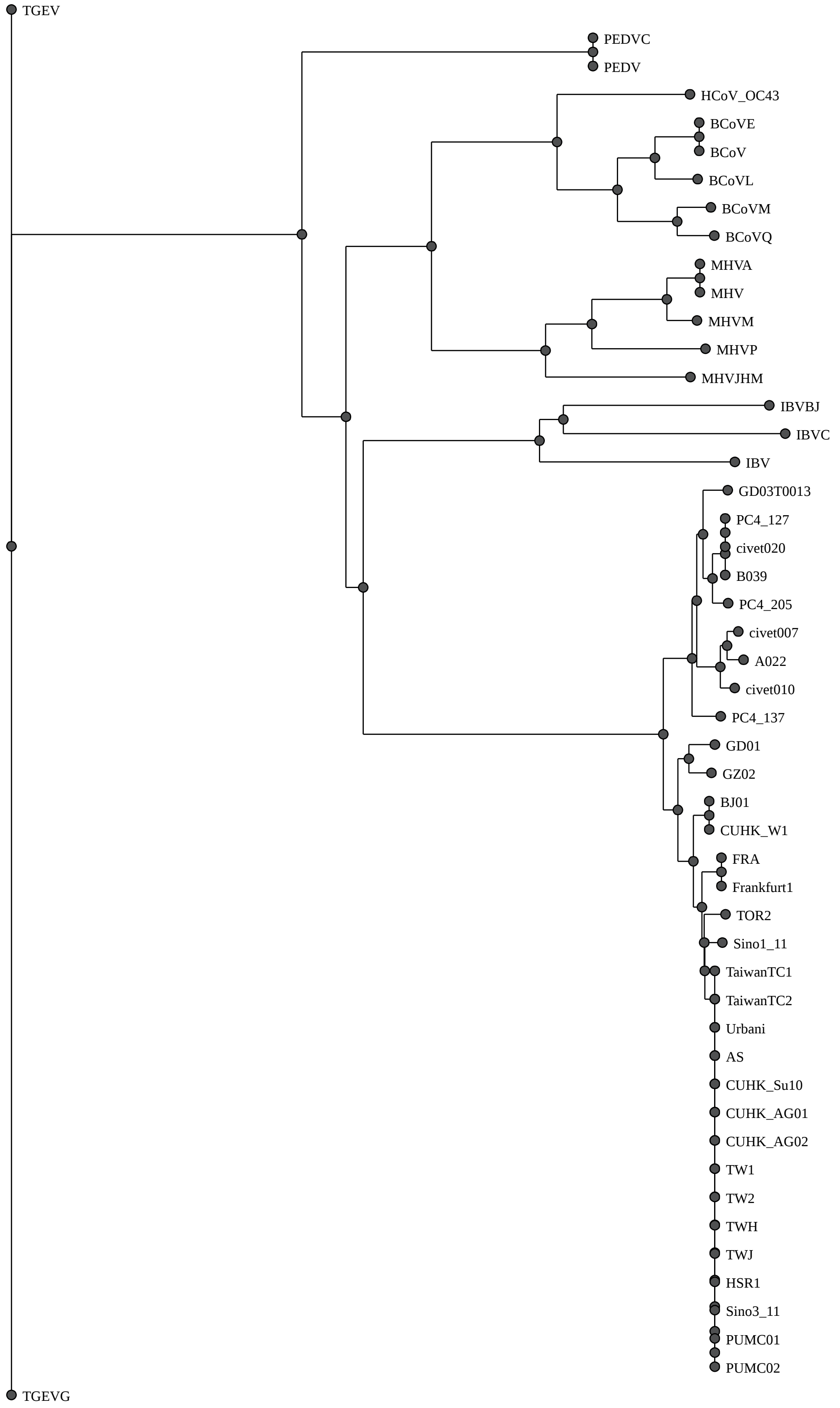

Figure 20: Base-Base Correlation (BBC) method.

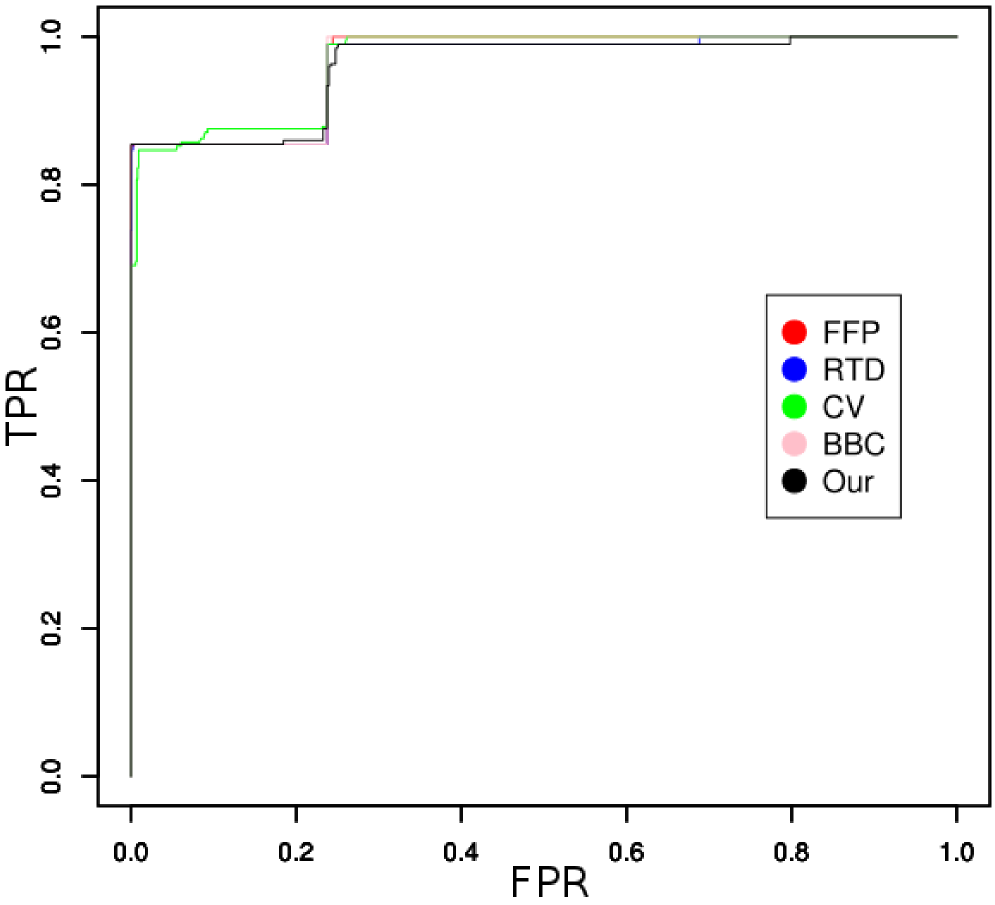

Datasets ➡	Coronavirus
**Methods ⬇**	AUC⬇
FFP	0.97
RTD	0.96
CV	0.97
BBC	0.97
OUR	**0.96**

Figure 21: Receiver operating characteristic curve (ROC) and Area Under the ROC Curve (AUC) of Coronavirus dataset using different method.

50 Coronavirus sequences were divided into four groups (Figure 15), where group I and II contains mammalian coronaviruses, group III contain avian coronaviruses and group IV contain SARS-CoVs. The phylogenetic tree (Figure 15) generated by our method put sequences belong to group I close to each other, which is missing in phylogenetic trees (Figures 16, 17, 18, 19 and 20) generated by other freely available tools[1]. Rest of the groups are properly clustered by all the methods. This shows the advantage of our method over others in terms of clustering. The AUC of our method is 0.96 (Figure 21), which indicates that our method has high accuracy (Table 1) and approximately same with other AUC's.

Phylogenetic tree on 50 Beta-globin protein sequences.

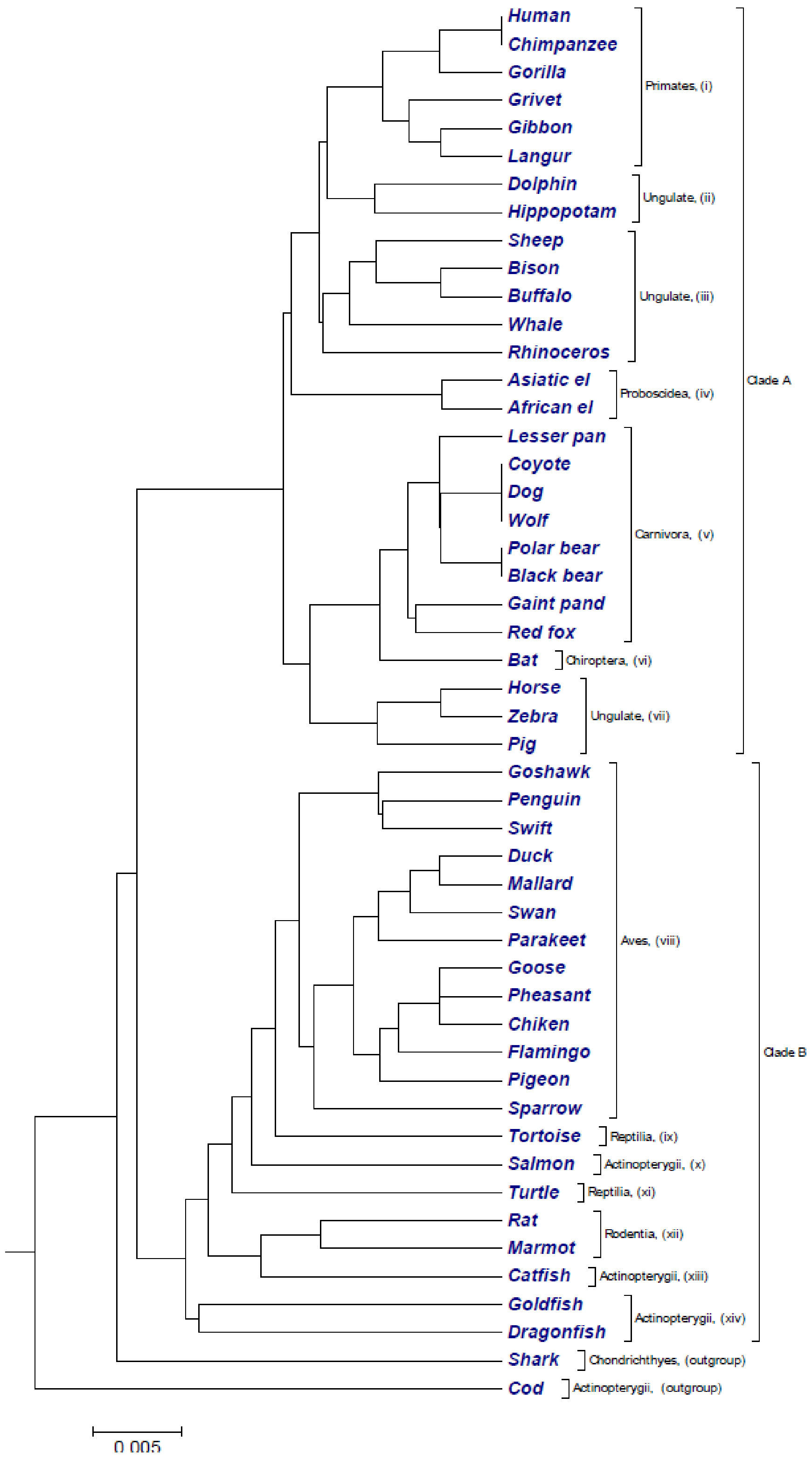

Figure 22: Our method.

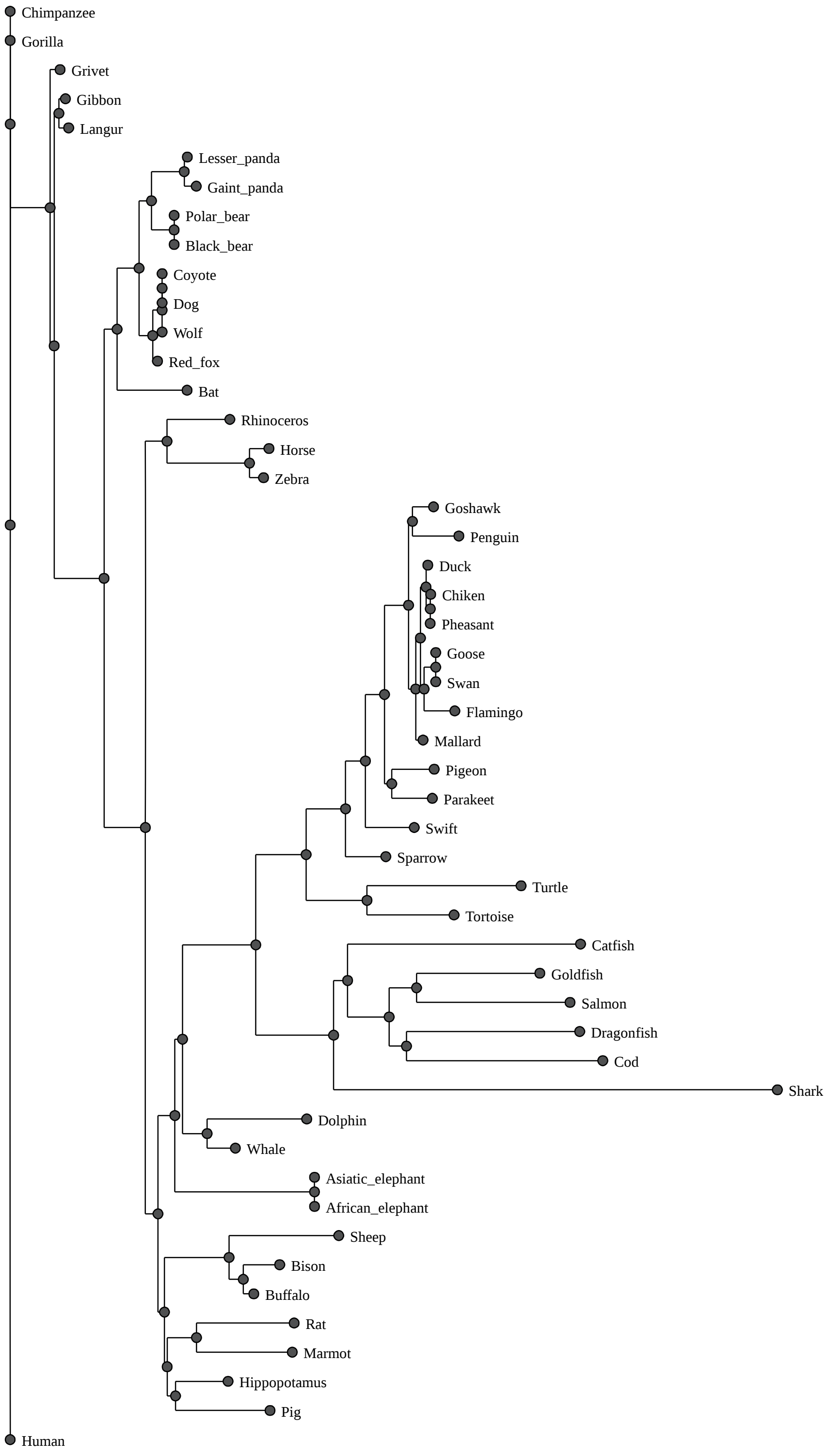

Figure 23: Feature Frequency Profiles (FFP) method.

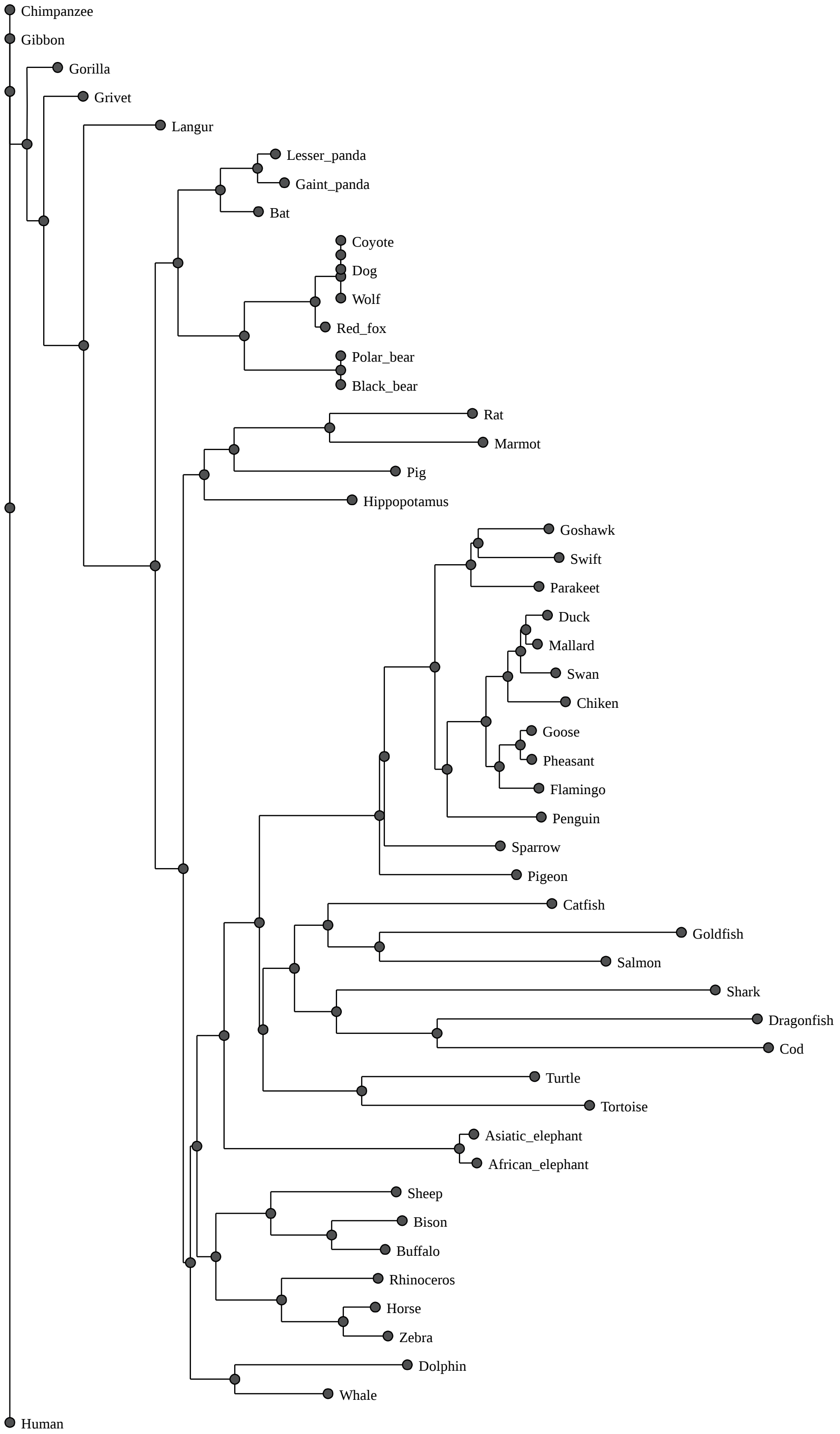

Figure 24: Return Time Distribution (RTD) method.

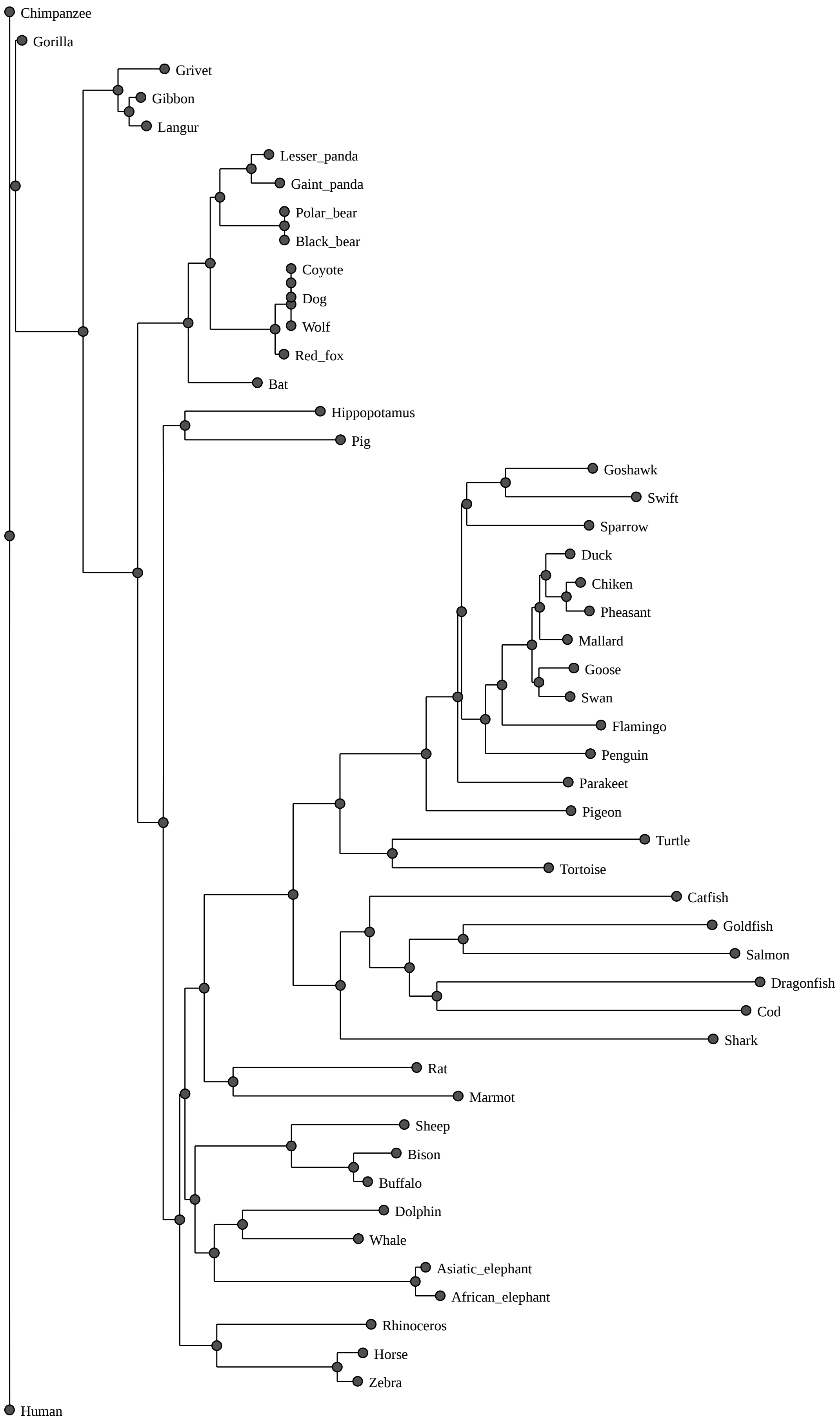

Figure 25: Composition distance (CV) method.

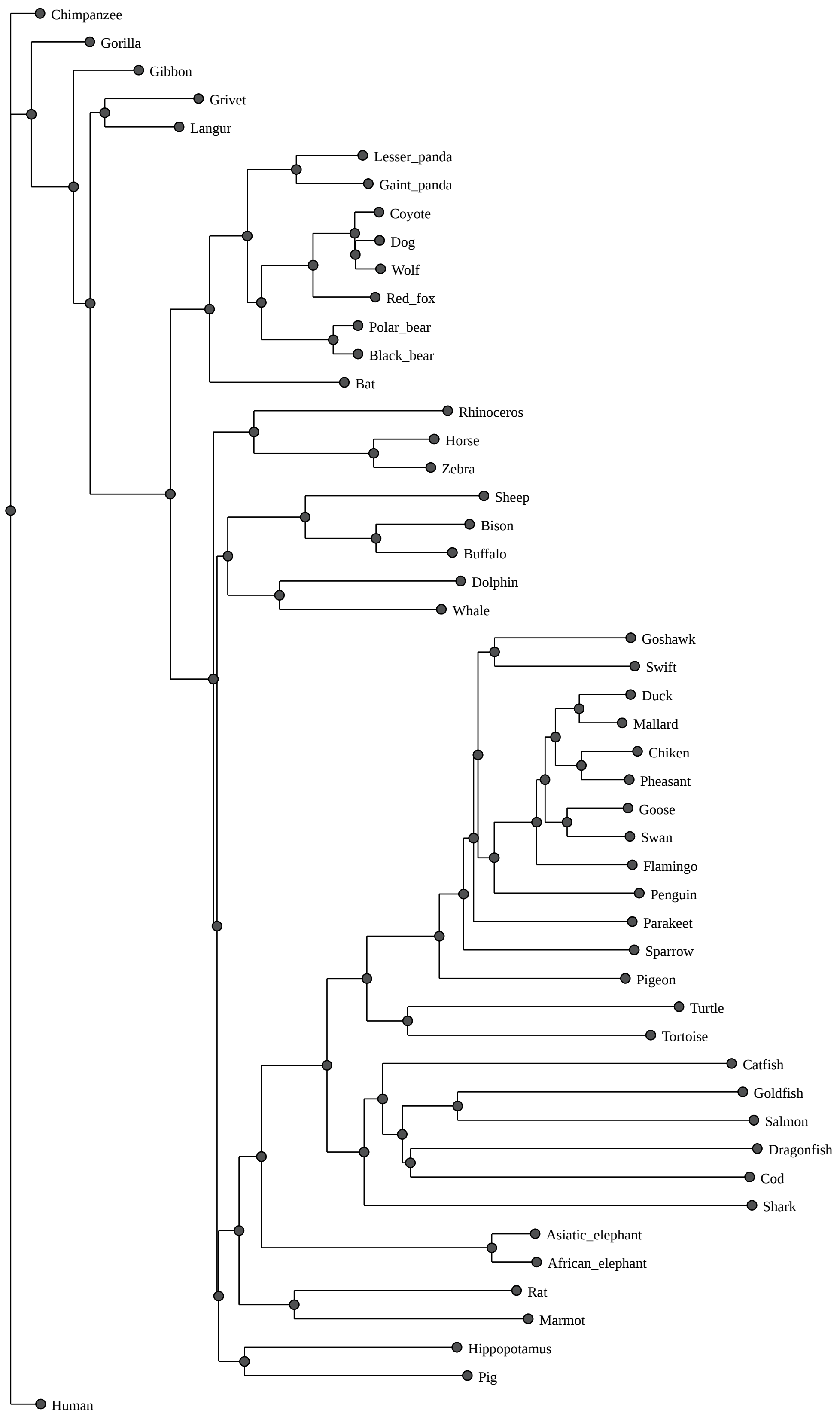

Figure 26: Normalized Compression Distance (NCD) method.

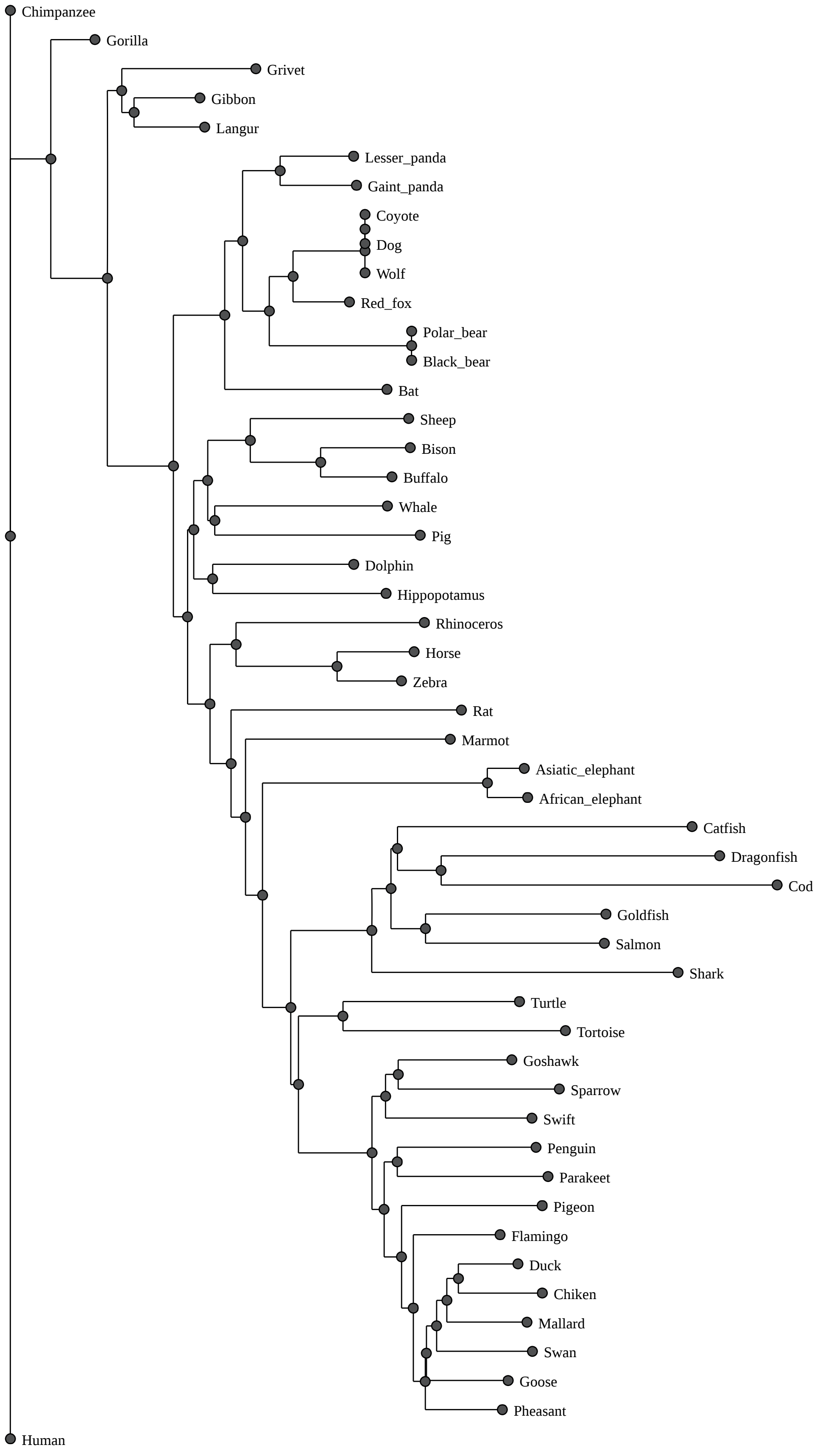

Figure 27: Base-Base Correlation (BBC) method.

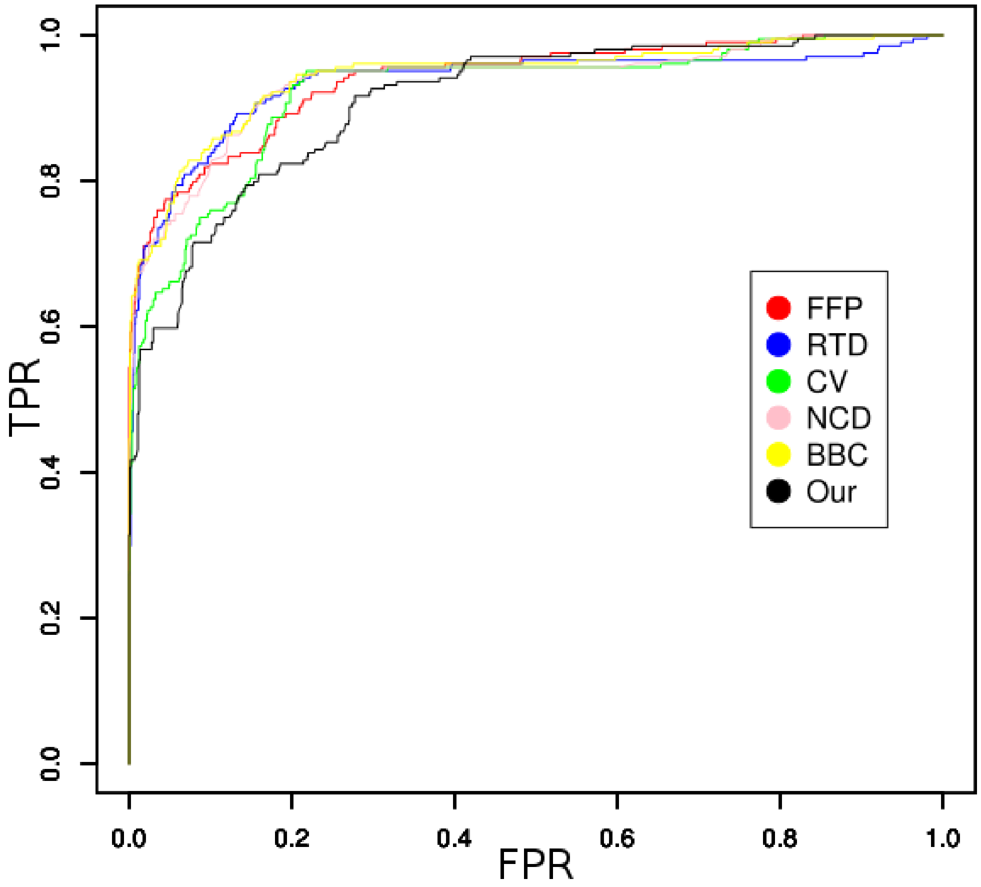

**Datasets ➡**	**Beta-globin**
**Methods ⬇**	AUC⬇
FFP	0.94
RTD	0.93
CV	0.92
NCD	0.94
BBC	0.94
OUR	**0.91**

Figure 28: Receiver operating characteristic curve (ROC) and Area Under the ROC Curve (AUC) of Beta-globin dataset using different method.

The phylogenetic trees constructed by our method (Figure 22) successfully clustered mammalian beta-globin protein sequences and primates in separate clades which are not observed in phylogenetic trees generated by other freely available tools [1] (Figures 23,24,25,26 and 27). This shows the advantage of our method over others based on clustering. The AUC of our method is 0.91 (Figure 28), which indicates that our method has high accuracy (Table 1) and approximately same with other AUC's.

Phylogenetic tree on 24 Transferrin protein sequences.

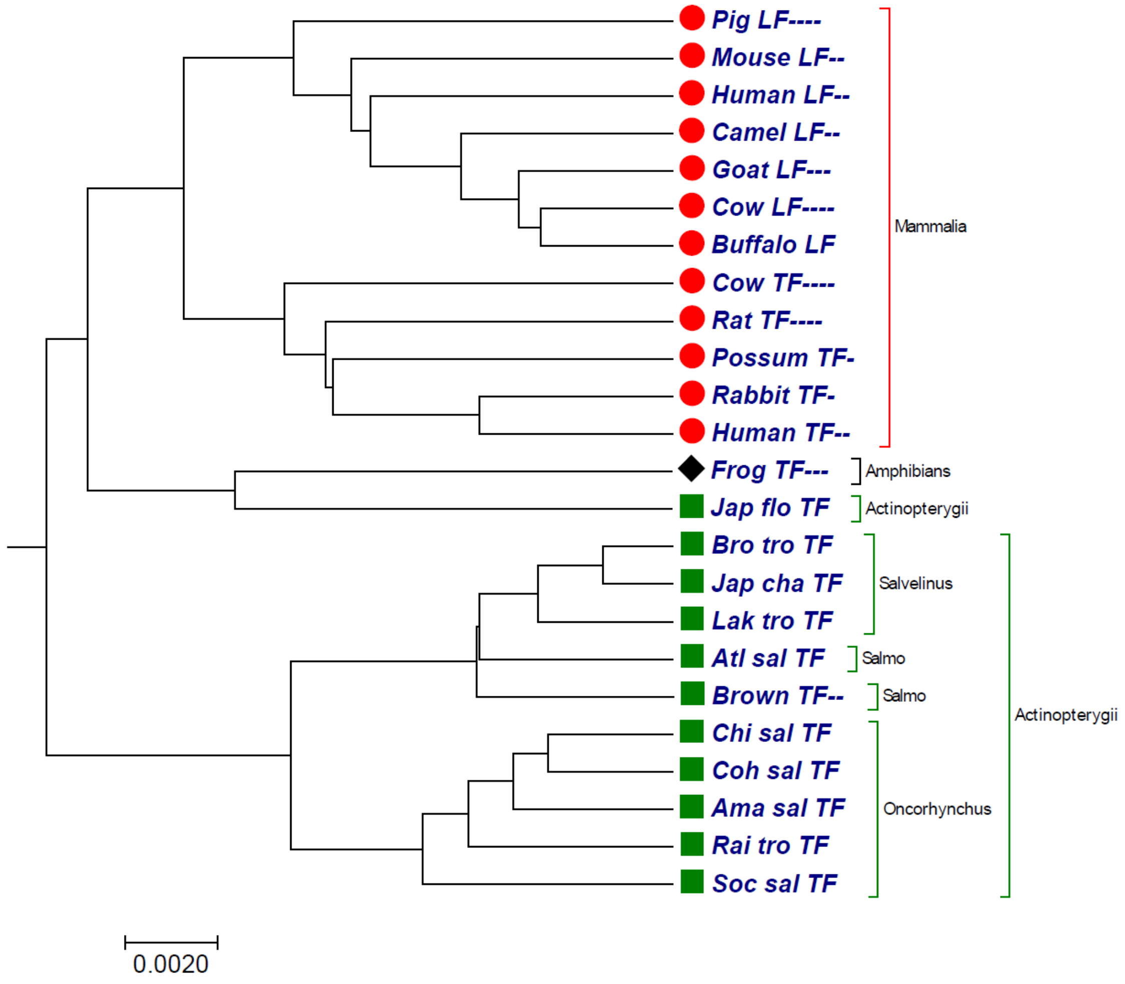

Figure 29: Our method.

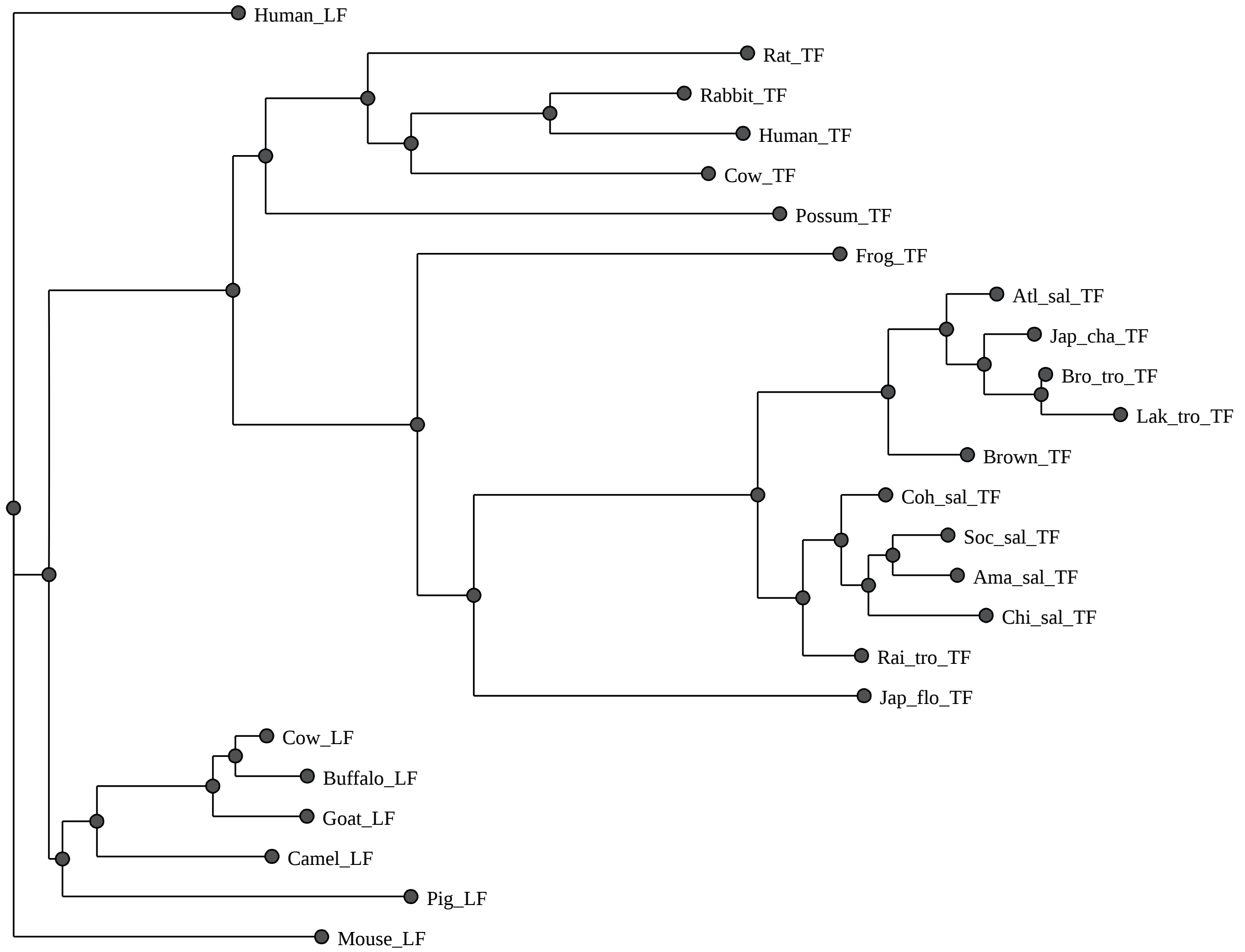

Figure 30: Feature Frequency Profiles (FFP) method.

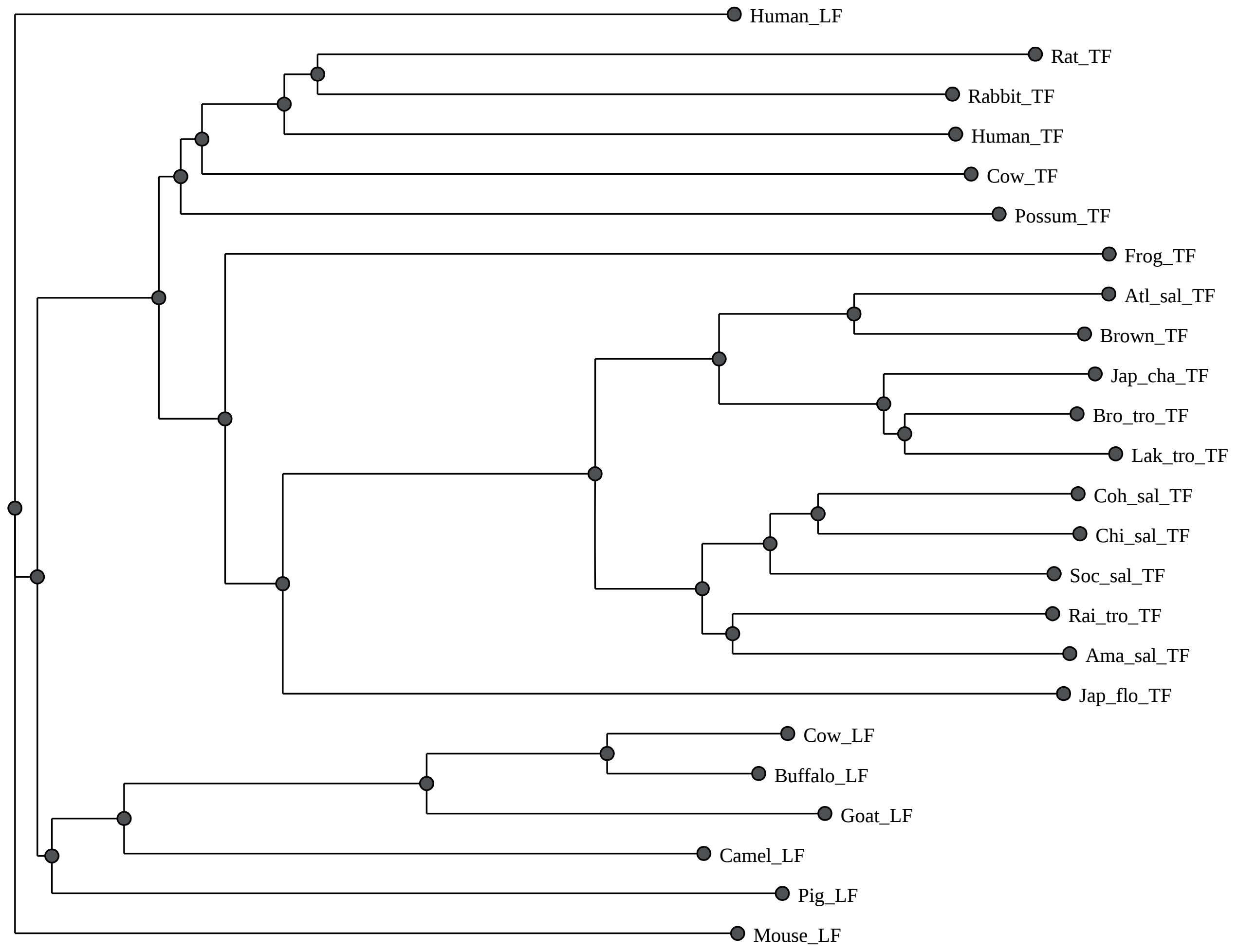

Figure 31: Return Time Distribution (RTD) method.

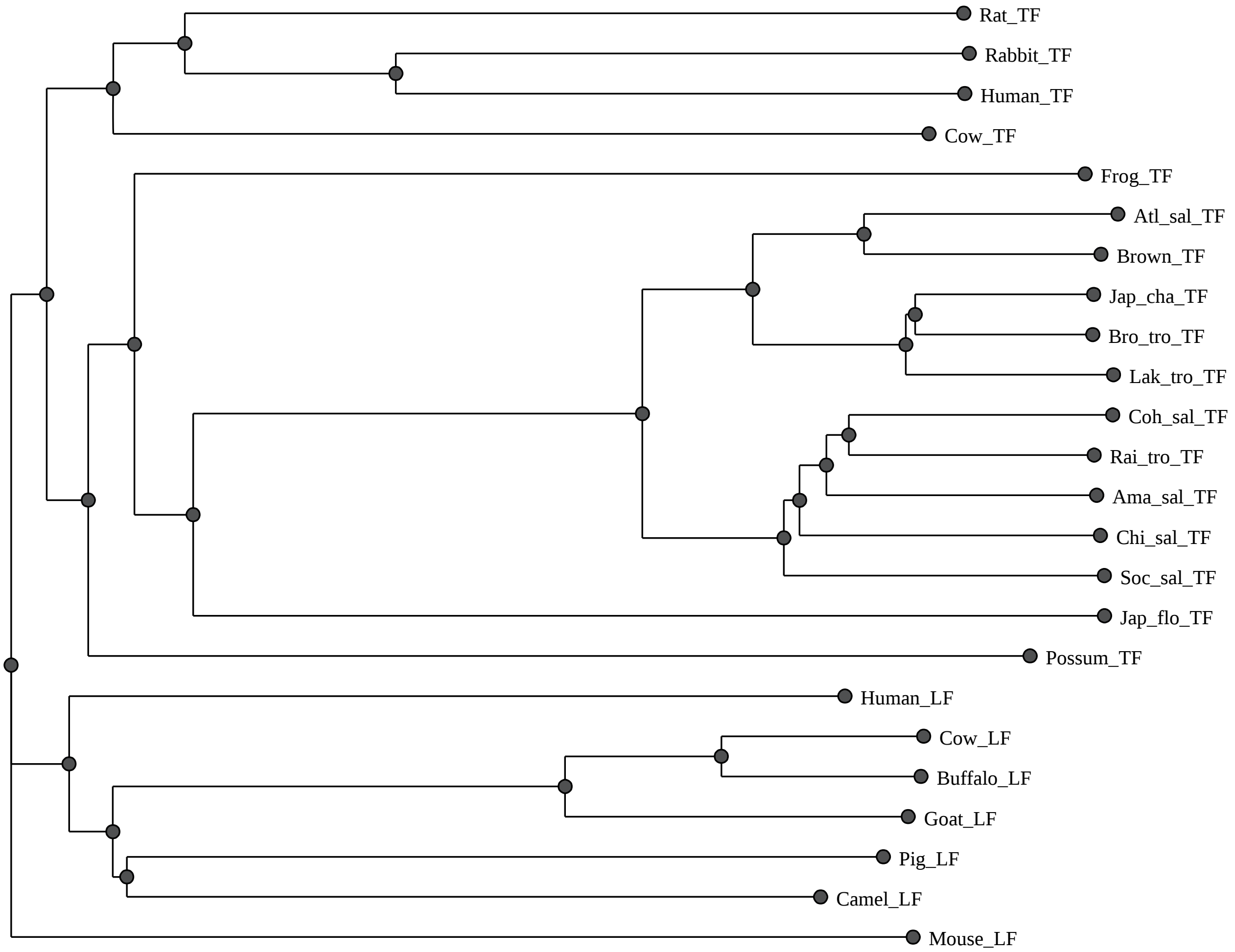

Figure 32: Composition distance (CV) method.

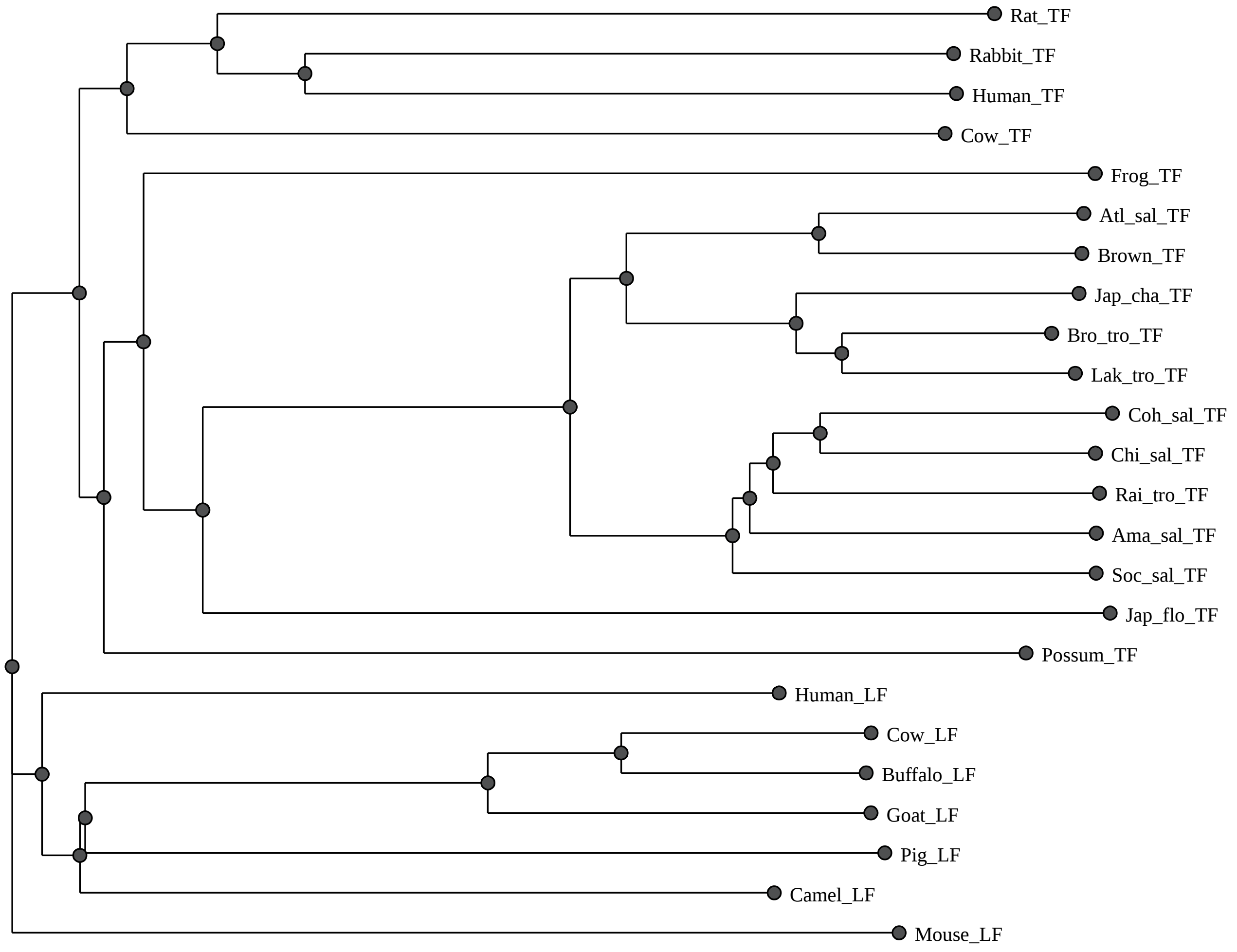

Figure 33: Normalized Compression Distance (NCD) method.

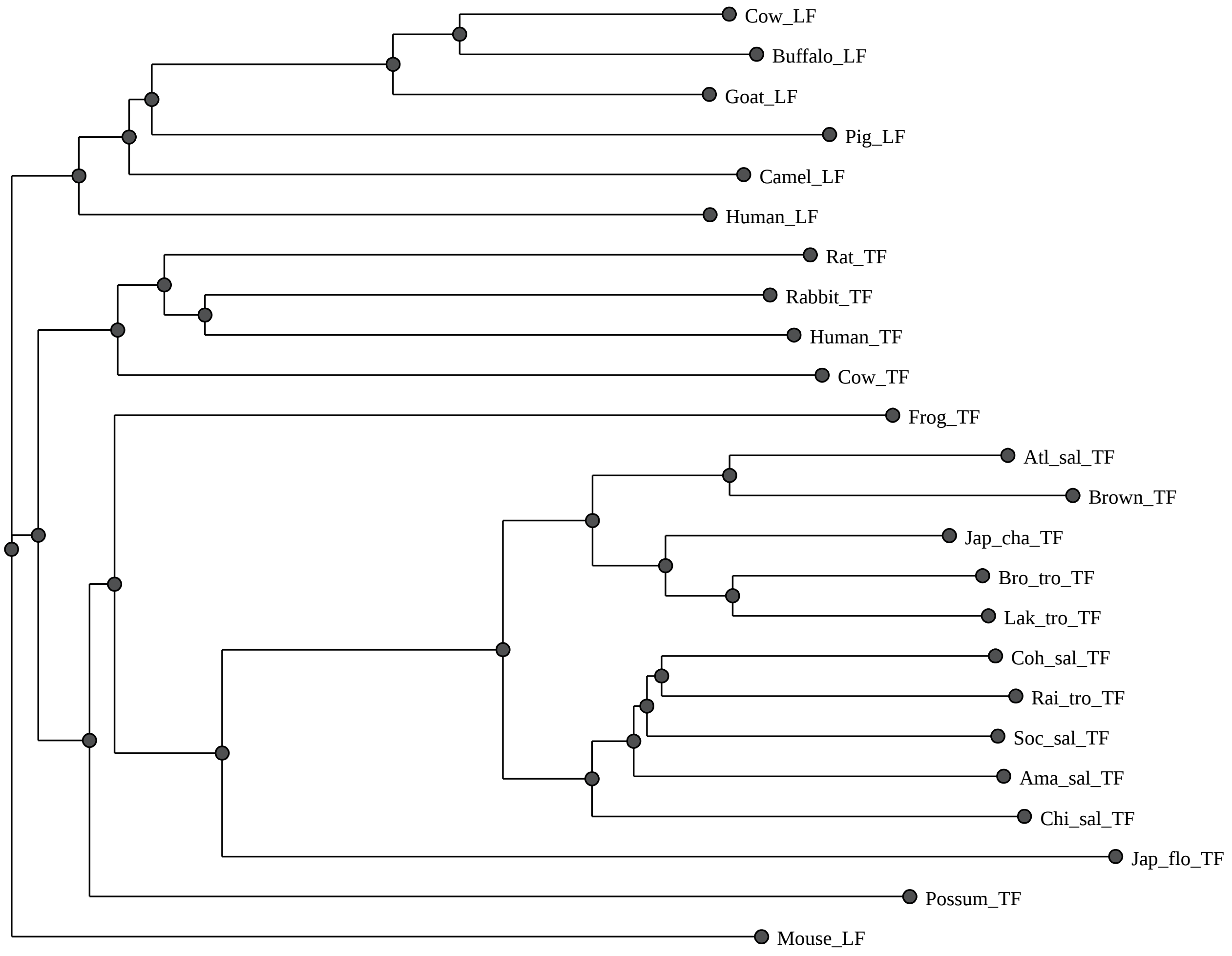

Figure 34: Base-Base Correlation (BBC) method.

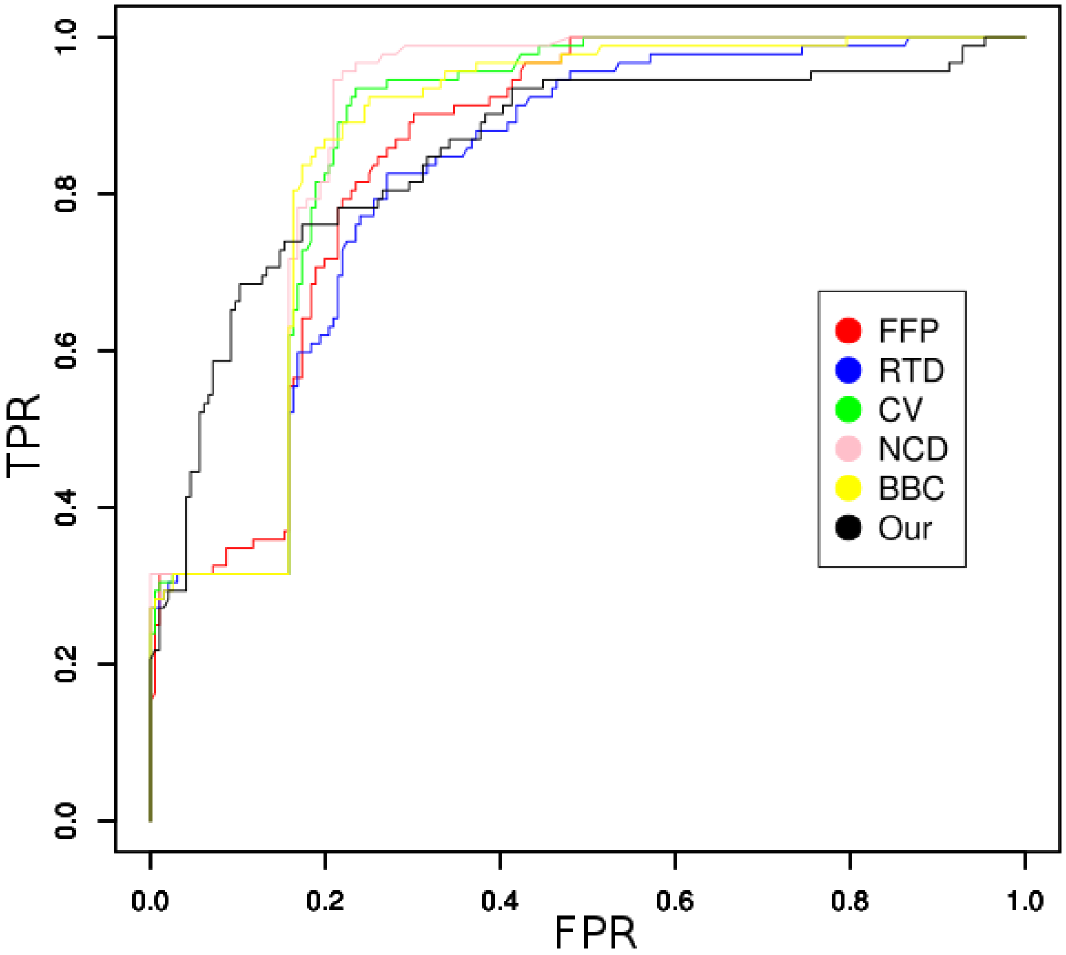

Datasets ➡	TFLF
**Methods ⬇**	AUC⬇
FFP	0.85
RTD	0.82
CV	0.86
NCD	0.88
BBC	0.86
OUR	**0.85**

Figure 35: Receiver operating characteristic curve (ROC) and Area Under the ROC Curve (AUC) of TFLF dataset using different method.

The phylogenetic trees constructed by our method (Figure 29) clustered sequences from mammalia in a separate clade which was not observed in phylogenetic trees generated by other freely available tools [1] (Figures 30, 31, 32, 33 and 34). This shows superiority of our method. Similarly, AUC of our method is 0.85 (Figure 35), which indicates that our method has moderate accuracy (Table 1) and similar with other AUC's.

Phylogenetic tree on Xylanases protein sequences in the F10 and G11 datasets.

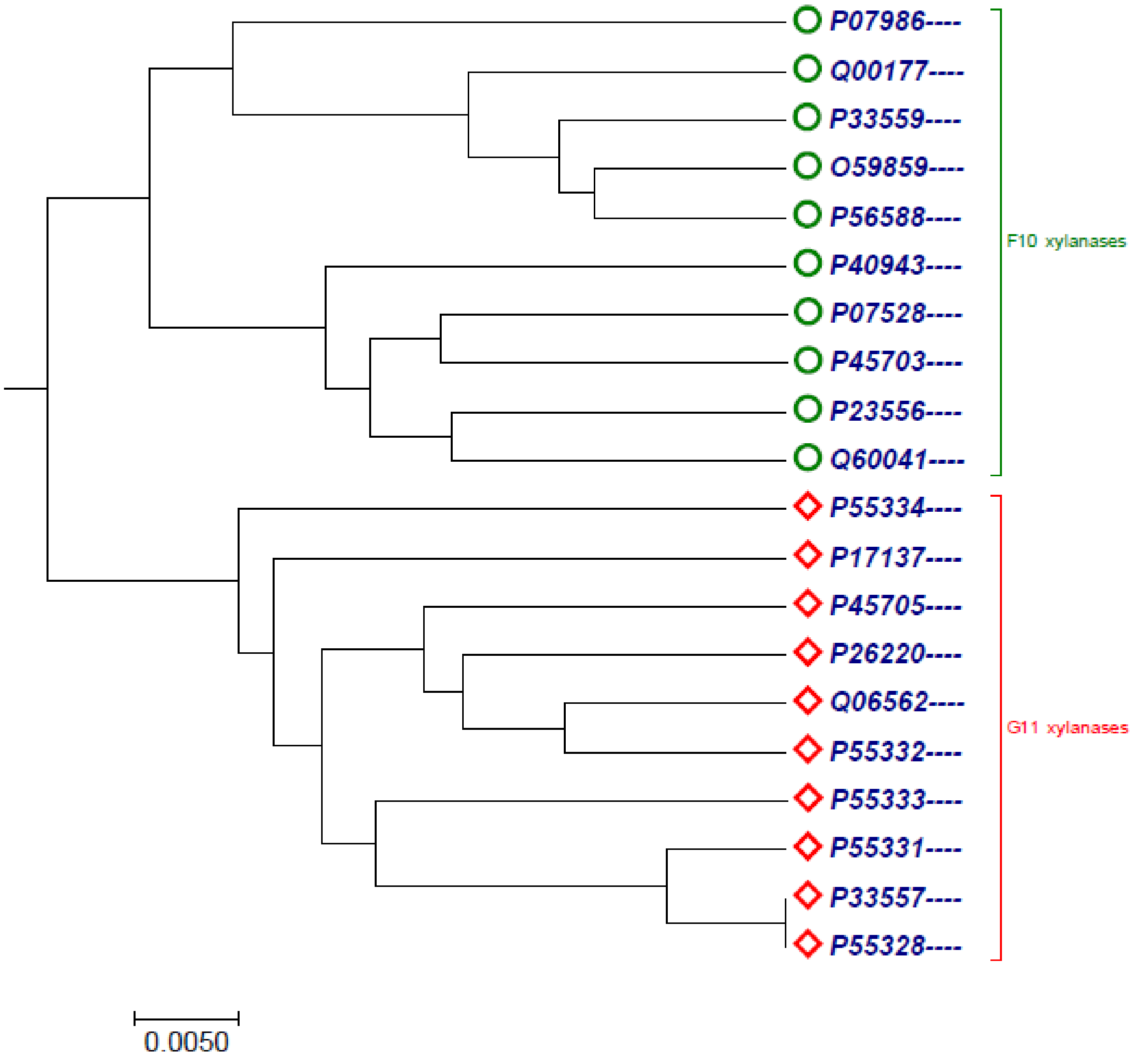

Figure 36: Our method.

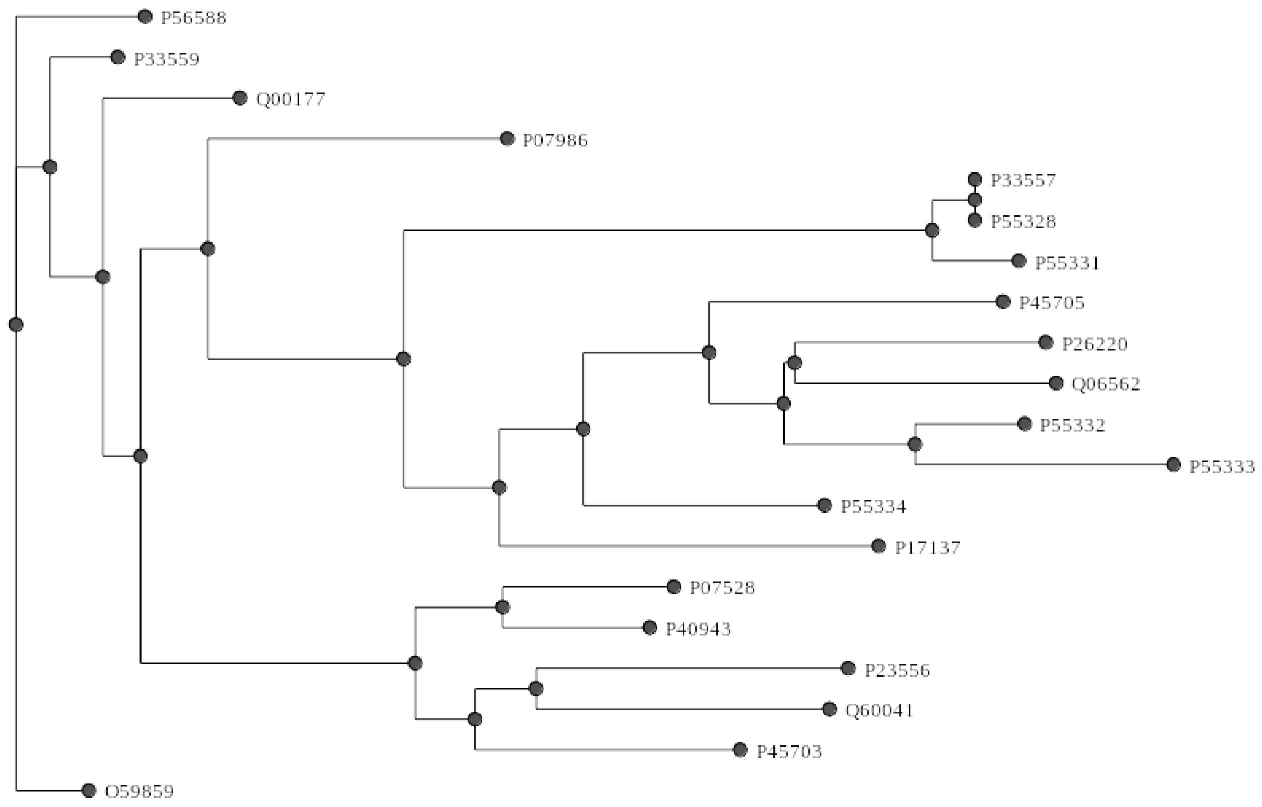

Figire 37: Feature Frequency Profiles (FFP) method.

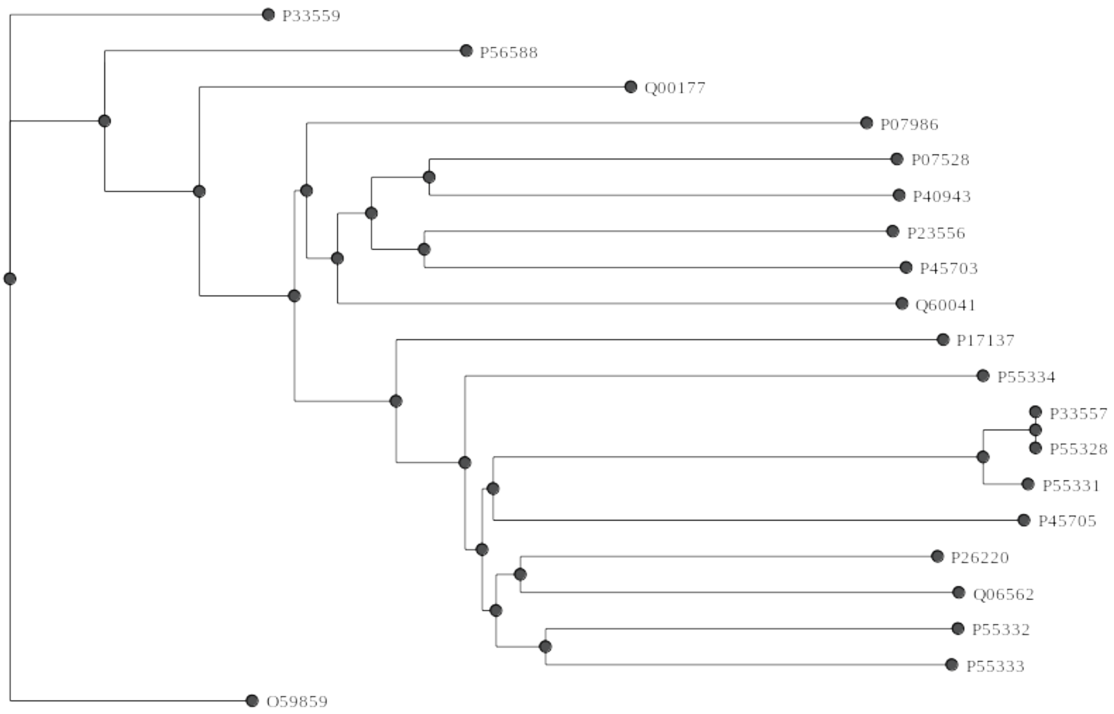

Figure 38: Return Time Distribution (RTD) method.

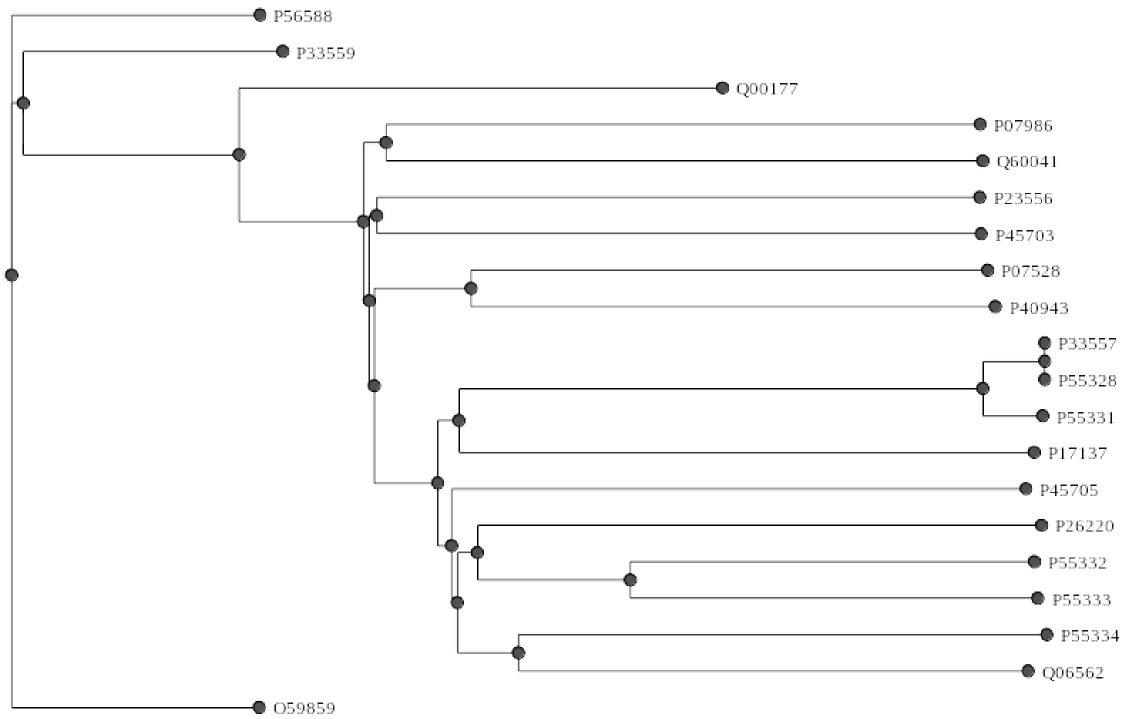

Figure 39: Composition distance (CV) method.

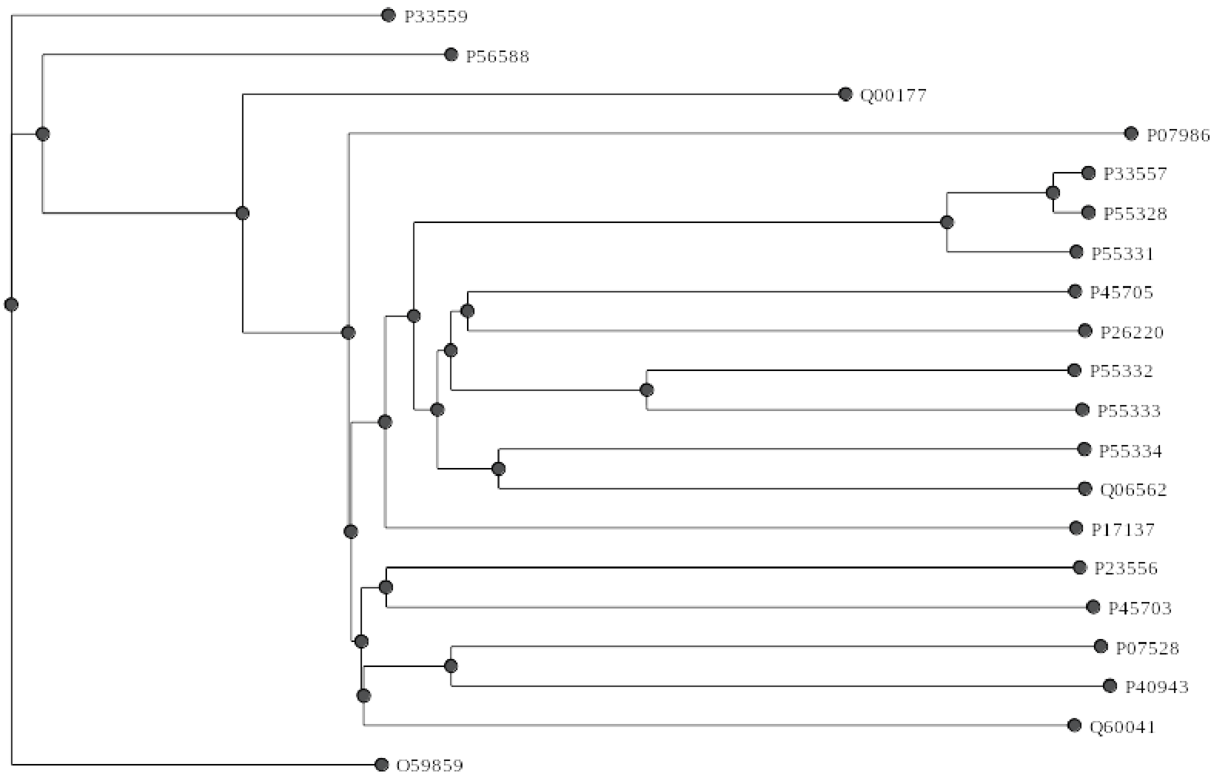

Figure 40: Normalized Compression Distance (NCD) method.

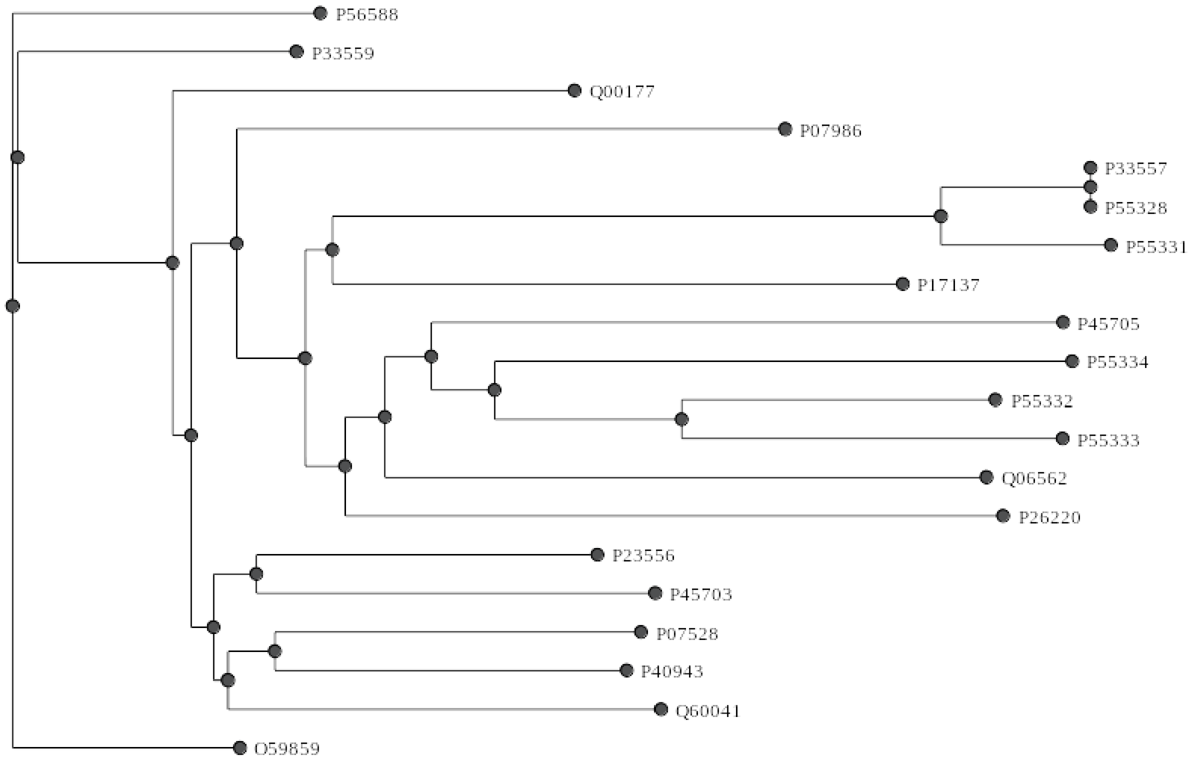

Figure 41: Base-Base Correlation (BBC) method.

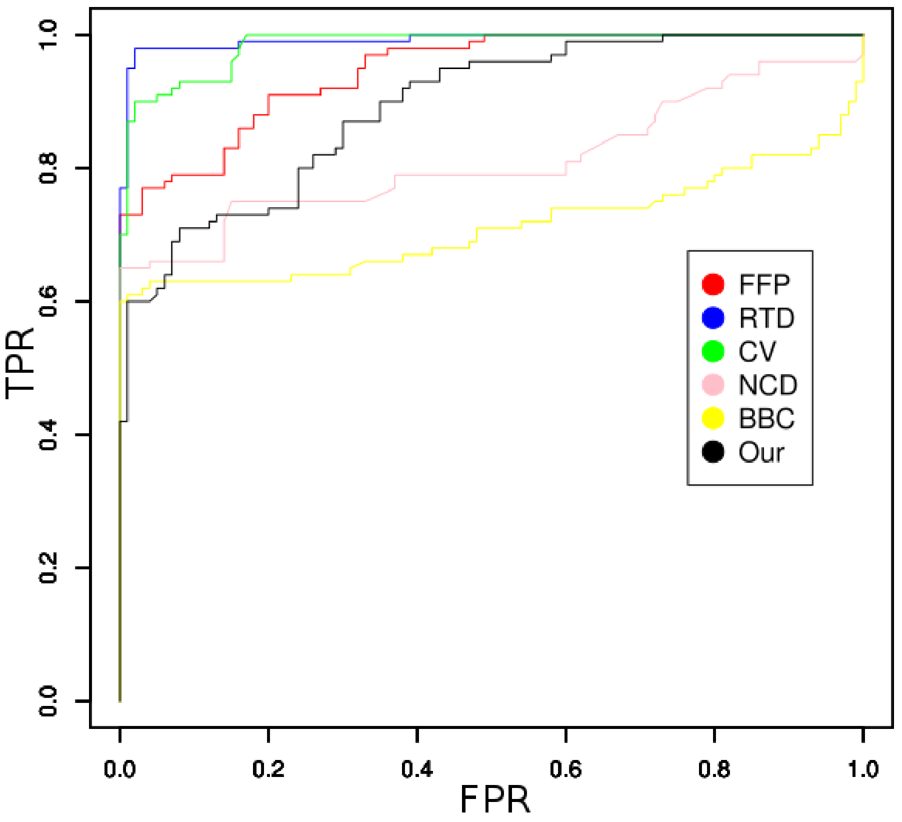

Datasets ➡	Xylanases
**Methods ⬇**	AUC⬇
FFP	0.95
RTD	0.99
CV	0.98
NCD	0.81
BBC	0.71
OUR	**0.89**

Figure 42: Receiver operating characteristic curve (ROC) and Area Under the ROC Curve (AUC) of Xylanases dataset using different method.

The above trees generated by our method and the other freely available recent methods [1] shows superiority of our method in terms of clustering over other methods. The sequences we used were Xylanases proteins from two different families F10 and G11. The tree generated by our method (Figure 36) distinctly clusters two families in two different clades, which is missing in all the other trees (Figures 37, 38, 39, 40 and 41). This shows the advantage of our method over others. Similarly, AUC of our method is 0.89 (Figure 42), which indicates that our method has moderate accuracy (Table 1).

Reference:

1. Zielezinski, A., Vinga, S., Almeida, J. & Karlowski, W. M. Alignment-free sequence comparison: benefits, applications, and tools. Genome Biol. 18, 186 (2017).
2. Swets, J. Measuring the accuracy of diagnostic systems. Sci. 240, 1285–1293 (1988).
3. NEMES, S. & HARTEL, T. Summary measures for binary classification systems in animal ecology. North-Western J. Zool. 6, 323–330 (2010).
4. Siriussawakul, A. et al. Predictive performance of a multivariable difficult intubation model for obese patients. PLOS ONE 1–15 (2018).
5. Antognoli, M. C. et al. Analysis of the diagnostic accuracy of the gamma interferon assay for detection of bovine tuberculosis in u.s. herds. Prev. Vet. Medicine 101, 35 – 41 (2011).
6. Zhu, W., Zeng, N. & Wang, N. Sensitivity, specificity, accuracy, associated confidence interval and roc analysis with practical SAS implementations. in Proceedings of the NESUG Health Care and Life Sciences, Baltimore, Md, USA (2010).
7. Sonego, P., Kocsor, A. & Pongor, S. Roc analysis: applications to the classification of biological sequences and 3d structures. Briefings Bioinforma. 9, 198–209 (2008).
