# Supplementary material for: Alignment-free similarity analysis for protein sequences based on fuzzy integral
Source: Sci Rep. 2019 Feb 26;9:2775. doi: 10.1038/s41598-019-39477-8 (PMC6391537; doi:10.1038/s41598-019-39477-8)
Supplement: Supplementary file 2 — Dataset 2 [file 41598_2019_39477_MOESM2_ESM.zip › Supplimentary_data/Our_method_distance_matrices/DISTANCE_MATRICES.pdf]

Alignment-free similarity analysis for protein sequences based on fuzzy integral.

Ajay Kumar Saw^1, Binod Chandra Tripathy^2, and Soumyadeep Nandi^3*
1. Institute of Advanced Study in Science and Technology, Mathematical Sciences Division, Guwahati-781035, India
2. Tripura University, Department of Mathematics, Agartala - 799022, India
3. Institute of Advanced Study in Science and Technology, Life Science Division, Guwahati-781035, India
*Corresponding author: soumyadeep.nandi@gmail.com

Distance matrices generated by our method.

1. NADH Dehydrogenase 5 (ND 5) protein sequences

9

AP_000649.	0.000000	0.013333	0.008267	0.005014	0.012218	0.013843	0.023917	0.027409	0.045254
NP_008222.	0.013333	0.000000	0.009975	0.013211	0.012106	0.013731	0.023856	0.027226	0.045193
NP_008209.	0.008267	0.009975	0.000000	0.006544	0.011354	0.013003	0.022041	0.027206	0.036987
NP_008196.	0.005014	0.013211	0.006544	0.000000	0.012990	0.014639	0.022194	0.027249	0.043531
NP_006899.	0.012218	0.012106	0.011354	0.012990	0.000000	0.003309	0.024063	0.027795	0.037334
NP_007066.	0.013843	0.013731	0.013003	0.014639	0.003309	0.000000	0.022411	0.026143	0.038982
AP_004902.	0.023917	0.023856	0.022041	0.022194	0.024063	0.022411	0.000000	0.011644	0.021337
NP_904338.	0.027409	0.027226	0.027206	0.027249	0.027795	0.026143	0.011644	0.000000	0.024134
NP_007105.	0.045254	0.045193	0.036987	0.043531	0.037334	0.038982	0.021337	0.024134	0.000000

2. NADH Dehydrogenase 6 (ND 6) protein sequences.

8

YP_0030240	0.000000	0.012979	0.041171	0.008480	0.048030	0.091837	0.062075	0.059637
NP_008197.	0.012979	0.000000	0.040899	0.013664	0.035051	0.078858	0.049096	0.046658
NP_007405.	0.041171	0.040899	0.000000	0.046505	0.026362	0.070169	0.040407	0.037969
NP_008223.	0.008480	0.013664	0.046505	0.000000	0.048715	0.092522	0.062760	0.060322
NP_006939.	0.048030	0.035051	0.026362	0.048715	0.000000	0.043807	0.033724	0.011607
AP_004903.	0.091837	0.078858	0.070169	0.092522	0.043807	0.000000	0.036240	0.032200
NP_904339.	0.062075	0.049096	0.040407	0.062760	0.033724	0.036240	0.000000	0.037684
NP_007080.	0.059637	0.046658	0.037969	0.060322	0.011607	0.032200	0.037684	0.000000

3. xylanases protein sequences in the F10 and G11 datasets.

20

O59859----	0.000000	0.018195	0.018814	0.031247	0.052233	0.062807	0.047736	0.072362	0.061995	0.068076	0.064319	0.064319	0.059257	0.073332	0.093777	0.064520	0.059654	0.074122	0.079911	0.075949
P56588----	0.018195	0.000000	0.027964	0.027076	0.049206	0.063890	0.053989	0.073958	0.049603	0.062854	0.058066	0.058066	0.053004	0.056322	0.080968	0.054728	0.045980	0.061313	0.073658	0.058939
P33559----	0.018814	0.027964	0.000000	0.033971	0.054237	0.047553	0.054289	0.066361	0.049675	0.055756	0.057766	0.057766	0.052914	0.061012	0.093713	0.054091	0.053443	0.074058	0.073358	0.063629
Q00177----	0.031247	0.027076	0.033971	0.000000	0.056168	0.065877	0.078983	0.073849	0.058322	0.073283	0.060196	0.060196	0.060179	0.069659	0.100106	0.060847	0.063589	0.080451	0.070963	0.072276
P07986----	0.052233	0.049206	0.054237	0.056168	0.000000	0.063368	0.062260	0.077773	0.072658	0.078739	0.081728	0.081728	0.076666	0.083995	0.080668	0.075183	0.052897	0.061013	0.097320	0.086612
P07528----	0.062807	0.063890	0.047553	0.065877	0.063368	0.000000	0.050584	0.050784	0.032952	0.035322	0.086256	0.086256	0.083008	0.066116	0.088804	0.059265	0.070118	0.069149	0.098503	0.074291
P40943----	0.047736	0.053989	0.054289	0.078983	0.062260	0.050584	0.000000	0.052556	0.057162	0.033139	0.092662	0.092662	0.093404	0.075477	0.096714	0.070932	0.073347	0.088133	0.092693	0.075551
P23556----	0.072362	0.073958	0.066361	0.073849	0.077773	0.050784	0.052556	0.000000	0.057362	0.031883	0.092862	0.092862	0.093604	0.088502	0.096749	0.082868	0.088800	0.082629	0.091691	0.072815
P45703----	0.061995	0.049603	0.049675	0.058322	0.072658	0.032952	0.057162	0.057362	0.000000	0.033038										

```
 0.077237  0.077237  0.072175  0.075214  0.096822  0.078334  0.073987  0.086651  0.092829  0.072150
Q60041----     0.068076  0.062854  0.055756  0.073283  0.078739  0.035322  0.033139  0.031883  0.033038  0.000000
 0.083188  0.083188  0.080403  0.075004  0.094759  0.059382  0.077524  0.075104  0.092515  0.078259
P33557----     0.064319  0.058066  0.057766  0.060196  0.081728  0.086256  0.092662  0.092862  0.077237  0.083188
 0.000000  0.000000  0.011327  0.043621  0.071734  0.069097  0.031464  0.052079  0.036783  0.048607
P55328----     0.064319  0.058066  0.057766  0.060196  0.081728  0.086256  0.092662  0.092862  0.077237  0.083188
 0.000000  0.000000  0.011327  0.043621  0.071734  0.069097  0.031464  0.052079  0.036783  0.048607
P55331----     0.059257  0.053004  0.052914  0.060179  0.076666  0.083008  0.093404  0.093604  0.072175  0.080403
 0.011327  0.011327  0.000000  0.053448  0.071474  0.064035  0.031204  0.051819  0.047180  0.056065
P45705----     0.073332  0.056322  0.061012  0.069659  0.083995  0.066116  0.075477  0.088502  0.075214  0.075004
 0.043621  0.043621  0.053448  0.000000  0.038140  0.043109  0.034397  0.032057  0.059213  0.036504
P26220----     0.093777  0.080968  0.093713  0.100106  0.080668  0.088804  0.096714  0.096749  0.096822  0.094759
 0.071734  0.071734  0.071474  0.038140  0.000000  0.054937  0.040270  0.026697  0.063454  0.074644
P55334----     0.064520  0.054728  0.054091  0.060847  0.075183  0.059265  0.070932  0.082868  0.078334  0.059382
 0.069097  0.069097  0.064035  0.043109  0.054937  0.000000  0.040266  0.051200  0.084689  0.047188
Q06562----     0.059654  0.045980  0.053443  0.063589  0.052897  0.070118  0.073347  0.088800  0.073987  0.077524
 0.031464  0.031464  0.031204  0.034397  0.040270  0.040266  0.000000  0.021065  0.044423  0.043709
P55332----     0.074122  0.061313  0.074058  0.080451  0.061013  0.069149  0.088133  0.082629  0.086651  0.075104
 0.052079  0.052079  0.051819  0.032057  0.026697  0.051200  0.021065  0.000000  0.052035  0.054989
P55333----     0.079911  0.073658  0.073358  0.070963  0.097320  0.098503  0.092693  0.091691  0.092829  0.092515
 0.036783  0.036783  0.047180  0.059213  0.063454  0.084689  0.044423  0.052035  0.000000  0.060408
P17137----     0.075949  0.058939  0.063629  0.072276  0.086612  0.074291  0.075551  0.072815  0.072150  0.078259
 0.048607  0.048607  0.056065  0.036504  0.074644  0.047188  0.043709  0.054989  0.060408  0.000000
```

4. transferrin protein sequences.

```
    24
Mouse_LF--     0.000000  0.020396  0.011119  0.014575  0.023101  0.019937  0.012442  0.023755  0.033723  0.015202
 0.024186  0.020569  0.032949  0.015374  0.025634  0.015262  0.019926  0.022842  0.023605  0.033754  0.032311  0.
032304  0.029363  0.022750
Rat_TF----     0.020396  0.000000  0.019518  0.027556  0.018484  0.021941  0.021115  0.012814  0.025446  0.016160
 0.022612  0.022898  0.023153  0.019963  0.015638  0.019851  0.022498  0.022503  0.022399  0.022398  0.022422  0.
023856  0.017022  0.019719
Cow_LF----     0.011119  0.019518  0.000000  0.012611  0.016190  0.026612  0.018807  0.015131  0.027647  0.005737
 0.022185  0.028836  0.034726  0.005685  0.025778  0.008648  0.030708  0.029290  0.029301  0.027732  0.029209  0.
026305  0.026337  0.033869
Human_LF--     0.014575  0.027556  0.012611  0.000000  0.023107  0.034473  0.017520  0.023169  0.030086  0.013060
 0.026383  0.021148  0.042764  0.013667  0.033816  0.012791  0.025702  0.020642  0.027200  0.027814  0.028682  0.
030129  0.030130  0.025221
Rabbit_TF-     0.023101  0.018484  0.016190  0.023107  0.000000  0.023933  0.031069  0.008316  0.030082  0.016306
 0.020035  0.026686  0.031033  0.016195  0.015970  0.017974  0.028558  0.027425  0.028904  0.030113  0.028670  0.
028663  0.027599  0.031719
Frog_TF---     0.019937  0.021941  0.026612  0.034473  0.023933  0.000000  0.032340  0.022722  0.035025  0.032041
 0.027876  0.032111  0.018845  0.029503  0.024601  0.027880  0.031059  0.036881  0.025360  0.032008  0.032001  0.
032007  0.030510  0.031175
Pig_LF----     0.012442  0.021115  0.018807  0.017520  0.031069  0.032340  0.000000  0.033929  0.030887  0.018839
 0.034177  0.027817  0.033525  0.017408  0.035808  0.017296  0.020097  0.023013  0.020769  0.030918  0.029475  0.
029468  0.026527  0.023075
Human_TF--     0.023755  0.012814  0.015131  0.023169  0.008316  0.022722  0.033929  0.000000  0.031242  0.017734
 0.013579  0.022787  0.023042  0.017961  0.013669  0.020834  0.022387  0.022392  0.022288  0.031273  0.029830  0.
029823  0.026882  0.025263
Atl_sal_TF     0.033723  0.025446  0.027647  0.030086  0.030082  0.035025  0.030887  0.031242  0.000000  0.027650
 0.043994  0.022850  0.032321  0.031902  0.030252  0.031790  0.021798  0.027620  0.016099  0.010117  0.007107  0.
008831  0.008834  0.021914
Goat_LF---     0.015202  0.016160  0.005737  0.013060  0.016306  0.032041  0.018839  0.017734  0.027650  0.000000
 0.022188  0.028839  0.030277  0.008564  0.025538  0.008306  0.030711  0.029293  0.029304  0.027735  0.029212  0.
026308  0.026340  0.033872
Cow_TF----     0.024186  0.022612  0.022185  0.026383  0.020035  0.027876  0.034177  0.013579  0.043994  0.022188
 0.000000  0.030840  0.016408  0.026440  0.016093  0.026328  0.023602  0.032333  0.033876  0.044025  0.042582  0.
042575  0.039634  0.028750
```

```
Coh_sal_TF     0.020569  0.022898  0.028836  0.021148  0.026686  0.032111  0.027817  0.022787  0.022850  0.028839
 0.030840  0.000000  0.021616  0.033091  0.025808  0.032979  0.011244  0.008473  0.009936  0.016911  0.019855  0.
014019  0.022684  0.005363
Jap_flo_TF     0.032949  0.023153  0.034726  0.042764  0.031033  0.018845  0.033525  0.023042  0.032321  0.030277
 0.016408  0.021616  0.000000  0.029097  0.018426  0.030245  0.017062  0.030089  0.022203  0.032352  0.030909  0.
030902  0.027961  0.019291
Buffalo_LF     0.015374  0.019963  0.005685  0.013667  0.016195  0.029503  0.017408  0.017961  0.031902  0.008564
 0.026440  0.033091  0.029097  0.000000  0.023000  0.011402  0.034963  0.033545  0.033556  0.031987  0.033464  0.
030560  0.030592  0.038124
Possum_TF-     0.025634  0.015638  0.025778  0.033816  0.015970  0.024601  0.035808  0.013669  0.030252  0.025538
 0.016093  0.025808  0.018426  0.023000  0.000000  0.022713  0.024756  0.030578  0.023209  0.030283  0.028840  0.
028833  0.025892  0.027777
Camel_LF--     0.015262  0.019851  0.008648  0.012791  0.017974  0.027880  0.017296  0.020834  0.031790  0.008306
 0.026328  0.032979  0.030245  0.011402  0.022713  0.000000  0.034851  0.033433  0.033444  0.031875  0.033352  0.
030448  0.030480  0.038012
Rai_tro_TF     0.019926  0.022498  0.030708  0.025702  0.028558  0.031059  0.020097  0.022387  0.021798  0.030711
 0.023602  0.011244  0.017062  0.034963  0.024756  0.034851  0.000000  0.013027  0.010274  0.020423  0.018980  0.
018973  0.021632  0.007107
Soc_sal_TF     0.022842  0.022503  0.029290  0.020642  0.027425  0.036881  0.023013  0.022392  0.027620  0.029293
 0.032333  0.008473  0.030089  0.033545  0.030578  0.033433  0.013027  0.000000  0.014525  0.021681  0.024625  0.
018789  0.027454  0.010798
Ama_sal_TF     0.023605  0.022399  0.029301  0.027200  0.028904  0.025360  0.020769  0.022288  0.016099  0.029304
 0.033876  0.009936  0.022203  0.033556  0.023209  0.033444  0.010274  0.014525  0.000000  0.010262  0.013104  0.
010261  0.015933  0.005815
Jap_cha_TF     0.033754  0.022398  0.027732  0.027814  0.030113  0.032008  0.030918  0.031273  0.010117  0.027735
 0.044025  0.016911  0.032352  0.031987  0.030283  0.031875  0.020423  0.021681  0.010262  0.000000  0.003010  0.
005793  0.010202  0.015975
Bro_tro_TF     0.032311  0.022422  0.029209  0.028682  0.028670  0.032001  0.029475  0.029830  0.007107  0.029212
 0.042582  0.019855  0.030909  0.033464  0.028840  0.033352  0.018980  0.024625  0.013104  0.003010  0.000000  0.
005836  0.007192  0.018919
Lak_tro_TF     0.032304  0.023856  0.026305  0.030129  0.028663  0.032007  0.029468  0.029823  0.008831  0.026308
 0.042575  0.014019  0.030902  0.030560  0.028833  0.030448  0.018973  0.018789  0.010261  0.005793  0.005836  0.
000000  0.008665  0.013825
Brown_TF--     0.029363  0.017022  0.026337  0.030130  0.027599  0.030510  0.026527  0.026882  0.008834  0.026340
 0.039634  0.022684  0.027961  0.030592  0.025892  0.030480  0.021632  0.027454  0.015933  0.010202  0.007192  0.
008665  0.000000  0.021748
Chi_sal_TF     0.022750  0.019719  0.033869  0.025221  0.031719  0.031175  0.023075  0.025263  0.021914  0.033872
 0.028750  0.005363  0.019291  0.038124  0.027777  0.038012  0.007107  0.010798  0.005815  0.015975  0.018919  0.
013825  0.021748  0.000000
```

5. coronavirus spike protein sequences.

```
    50
TGEVG-----     0.000000  0.000000  0.018179  0.018179  0.018639  0.012797  0.012799  0.015868  0.016602  0.012797
 0.022065  0.020896  0.021946  0.022815  0.022065  0.012511  0.014805  0.016335  0.016264  0.016264  0.016264  0.
016263  0.017421  0.016623  0.016264  0.017421  0.016264  0.016270  0.017067  0.017066  0.017066  0.017864  0.
017066  0.017066  0.017066  0.017066  0.017066  0.017066  0.017864  0.017066  0.017066  0.017066  0.017066  0.
017066  0.017066  0.017066  0.017066  0.017066  0.017066  0.017066
TGEV------     0.000000  0.000000  0.018179  0.018179  0.018639  0.012797  0.012799  0.015868  0.016602  0.012797
 0.022065  0.020896  0.021946  0.022815  0.022065  0.012511  0.014805  0.016335  0.016264  0.016264  0.016264  0.
016263  0.017421  0.016623  0.016264  0.017421  0.016264  0.016270  0.017067  0.017066  0.017066  0.017864  0.
017066  0.017066  0.017066  0.017066  0.017066  0.017066  0.017864  0.017066  0.017066  0.017066  0.017066  0.
017066  0.017066  0.017066  0.017066  0.017066  0.017066  0.017066
PEDVC-----     0.018179  0.018179  0.000000  0.000000  0.021608  0.018540  0.017805  0.017820  0.017087  0.018540
 0.017864  0.019826  0.017285  0.018626  0.017864  0.017685  0.021033  0.018964  0.021708  0.021708  0.021708  0.
021708  0.021708  0.021708  0.021708  0.021708  0.021708  0.020123  0.020123  0.020921  0.020921  0.021719  0.
020920  0.020920  0.020920  0.020920  0.020920  0.020920  0.021719  0.020920  0.020920  0.020920  0.020920  0.
020920  0.020920  0.020920  0.020919  0.020920  0.020920  0.020920
PEDV------     0.018179  0.018179  0.000000  0.000000  0.021608  0.018540  0.017805  0.017820  0.017087  0.018540
 0.017864  0.019826  0.017285  0.018626  0.017864  0.017685  0.021033  0.018964  0.021708  0.021708  0.021708  0.
```

```
021708  0.021708  0.021708  0.021708  0.021708  0.021708  0.020123  0.020123  0.020921  0.020921  0.021719  0.
020920  0.020920  0.020920  0.020920  0.020920  0.020920  0.021719  0.020920  0.020920  0.020920  0.020920  0.
020920  0.020920  0.020920  0.020919  0.020920  0.020920  0.020920
HCoV-OC43-     0.018639  0.018639  0.021608  0.021608  0.000000  0.006490  0.007223  0.005050  0.005050  0.006490
 0.007465  0.008300  0.009629  0.007460  0.007465  0.012164  0.015286  0.013238  0.019255  0.019255  0.019255  0.
019255  0.020850  0.020052  0.019255  0.020850  0.019255  0.018459  0.018459  0.018459  0.018459  0.018459  0.
018459  0.018459  0.018459  0.018459  0.018459  0.018459  0.018459  0.018459  0.018459  0.018459  0.018459  0.
018459  0.018459  0.018459  0.018459  0.018459  0.018459  0.018459
BCoVE-----     0.012797  0.012797  0.018540  0.018540  0.006490  0.000000  0.001468  0.003719  0.004453  0.000000
 0.009916  0.010683  0.009797  0.010666  0.009916  0.010250  0.012946  0.012765  0.016187  0.016187  0.016187  0.
016187  0.017782  0.016984  0.016187  0.017782  0.016187  0.015391  0.015391  0.015391  0.015391  0.015391  0.
015391  0.015391  0.015391  0.015391  0.015391  0.015391  0.015391  0.015391  0.015391  0.015391  0.015391  0.
015391  0.015391  0.015391  0.015391  0.015391  0.015391  0.015391
BCoVL-----     0.012799  0.012799  0.017805  0.017805  0.007223  0.001468  0.000000  0.004452  0.005186  0.001468
 0.010649  0.009960  0.010530  0.011399  0.010649  0.010250  0.012945  0.013497  0.015452  0.015452  0.015452  0.
015452  0.017047  0.016249  0.015452  0.017047  0.015452  0.014656  0.014656  0.014656  0.014656  0.014656  0.
014656  0.014656  0.014656  0.014656  0.014656  0.014656  0.014656  0.014656  0.014656  0.014656  0.014656  0.
014656  0.014656  0.014656  0.014656  0.014656  0.014656  0.014656
BCoVM-----     0.015868  0.015868  0.017820  0.017820  0.005050  0.003719  0.004452  0.000000  0.001468  0.003719
 0.009307  0.009948  0.010915  0.009310  0.009307  0.011073  0.014421  0.014244  0.015467  0.015467  0.015467  0.
015467  0.017062  0.016264  0.015467  0.017062  0.015467  0.014671  0.014671  0.014671  0.014671  0.015107  0.
014671  0.014671  0.014671  0.014671  0.014671  0.014671  0.015107  0.014671  0.014671  0.014671  0.014671  0.
014671  0.014671  0.014671  0.014671  0.014671  0.014671  0.014671
BCoVQ-----     0.016602  0.016602  0.017087  0.017087  0.005050  0.004453  0.005186  0.001468  0.000000  0.004453
 0.008573  0.009948  0.010181  0.008576  0.008573  0.010340  0.013688  0.014244  0.014734  0.014734  0.014734  0.
014734  0.016329  0.015531  0.014734  0.016329  0.014734  0.013938  0.013938  0.013938  0.013938  0.014374  0.
013938  0.013938  0.013938  0.013938  0.013938  0.013938  0.014374  0.013938  0.013938  0.013938  0.013938  0.
013938  0.013938  0.013938  0.013938  0.013938  0.013938  0.013938
BCoV------     0.012797  0.012797  0.018540  0.018540  0.006490  0.000000  0.001468  0.003719  0.004453  0.000000
 0.009916  0.010683  0.009797  0.010666  0.009916  0.010250  0.012946  0.012765  0.016187  0.016187  0.016187  0.
016187  0.017782  0.016984  0.016187  0.017782  0.016187  0.015391  0.015391  0.015391  0.015391  0.015391  0.
015391  0.015391  0.015391  0.015391  0.015391  0.015391  0.015391  0.015391  0.015391  0.015391  0.015391  0.
015391  0.015391  0.015391  0.015391  0.015391  0.015391  0.015391
MHVA------     0.022065  0.022065  0.017864  0.017864  0.007465  0.009916  0.010649  0.009307  0.008573  0.009916
 0.000000  0.007246  0.004438  0.001504  0.000000  0.009576  0.014721  0.010850  0.015535  0.015535  0.015535  0.
015534  0.017106  0.016308  0.015535  0.017106  0.015535  0.015541  0.016338  0.016337  0.016337  0.017135  0.
016337  0.016337  0.016337  0.016337  0.016337  0.016337  0.017135  0.016337  0.016337  0.016337  0.016337  0.
016337  0.016337  0.016337  0.016337  0.016337  0.016337  0.016337
MHVJHM----     0.020896  0.020896  0.019826  0.019826  0.008300  0.010683  0.009960  0.009948  0.009948  0.010683
 0.007246  0.000000  0.010472  0.007247  0.007246  0.012935  0.013735  0.014524  0.018137  0.018137  0.018137  0.
018136  0.018136  0.018137  0.018137  0.018137  0.018137  0.018143  0.018940  0.018939  0.018939  0.019737  0.
018939  0.018939  0.018939  0.018939  0.018939  0.018939  0.019737  0.018939  0.018939  0.018939  0.018939  0.
018939  0.018939  0.018939  0.018939  0.018939  0.018939  0.018939
MHVP------     0.021946  0.021946  0.017285  0.017285  0.009629  0.009797  0.010530  0.010915  0.010181  0.009797
 0.004438  0.010472  0.000000  0.004440  0.004438  0.010115  0.014602  0.011731  0.014932  0.014932  0.014932  0.
014932  0.016527  0.015729  0.014932  0.016527  0.014932  0.014136  0.014136  0.014136  0.014136  0.014748  0.
014136  0.014136  0.014136  0.014136  0.014136  0.014136  0.014748  0.014136  0.014136  0.014136  0.014136  0.
014136  0.014136  0.014136  0.014136  0.014136  0.014136  0.014136
MHVM------     0.022815  0.022815  0.018626  0.018626  0.007460  0.010666  0.011399  0.009310  0.008576  0.010666
 0.001504  0.007247  0.004440  0.000000  0.001504  0.010326  0.015471  0.010081  0.016273  0.016273  0.016273  0.
016273  0.017868  0.017070  0.016273  0.017868  0.016273  0.015536  0.016333  0.016332  0.016332  0.017130  0.
016332  0.016332  0.016332  0.016332  0.016332  0.016332  0.017130  0.016332  0.016332  0.016332  0.016332  0.
016332  0.016332  0.016332  0.016332  0.016332  0.016332  0.016332
MHV-------     0.022065  0.022065  0.017864  0.017864  0.007465  0.009916  0.010649  0.009307  0.008573  0.009916
 0.000000  0.007246  0.004438  0.001504  0.000000  0.009576  0.014721  0.010850  0.015535  0.015535  0.015535  0.
015534  0.017106  0.016308  0.015535  0.017106  0.015535  0.015541  0.016338  0.016337  0.016337  0.017135  0.
016337  0.016337  0.016337  0.016337  0.016337  0.016337  0.017135  0.016337  0.016337  0.016337  0.016337  0.
016337  0.016337  0.016337  0.016337  0.016337  0.016337  0.016337
IBVBJ-----     0.012511  0.012511  0.017685  0.017685  0.012164  0.010250  0.010250  0.011073  0.010340  0.010250
 0.009576  0.012935  0.010115  0.010326  0.009576  0.000000  0.007699  0.005750  0.015426  0.014628  0.015425  0.
014627  0.013830  0.013830  0.014628  0.013830  0.014628  0.016221  0.016221  0.015424  0.015424  0.014627  0.
015012  0.014627  0.014627  0.014627  0.014627  0.014627  0.014627  0.014627  0.014627  0.014627  0.014627  0.
```

```
014627  0.014627  0.014627  0.014627  0.014627  0.014627  0.014627
IBVC------     0.014805  0.014805  0.021033  0.021033  0.015286  0.012946  0.012945  0.014421  0.013688  0.012946
 0.014721  0.013735  0.014602  0.015471  0.014721  0.007699  0.000000  0.008991  0.017137  0.016339  0.017136  0.
016338  0.015541  0.015541  0.016339  0.015541  0.016339  0.017932  0.017932  0.017135  0.017135  0.016338  0.
016338  0.016338  0.016338  0.016338  0.016338  0.016338  0.016338  0.016338  0.016338  0.016338  0.016338  0.
016338  0.016338  0.016338  0.016338  0.016338  0.016338  0.016338
IBV-------     0.016335  0.016335  0.018964  0.018964  0.013238  0.012765  0.013497  0.014244  0.014244  0.012765
 0.010850  0.014524  0.011731  0.010081  0.010850  0.005750  0.008991  0.000000  0.021176  0.020378  0.021175  0.
020377  0.019580  0.019580  0.020378  0.019580  0.020378  0.021971  0.021971  0.021174  0.021174  0.020377  0.
020377  0.020377  0.020377  0.020377  0.020377  0.020377  0.020377  0.020377  0.020377  0.020377  0.020377  0.
020377  0.020377  0.020377  0.020377  0.020377  0.020377  0.020377
GD03T0013-     0.016264  0.016264  0.021708  0.021708  0.019255  0.016187  0.015452  0.015467  0.014734  0.016187
 0.015535  0.018137  0.014932  0.016273  0.015535  0.015426  0.017137  0.021176  0.000000  0.000798  0.000798  0.
000799  0.001596  0.001596  0.000798  0.001603  0.000798  0.002411  0.003209  0.002411  0.002411  0.002409  0.
003206  0.002409  0.002409  0.002409  0.002409  0.002409  0.002409  0.002409  0.002409  0.002409  0.002409  0.
002409  0.002409  0.002409  0.002402  0.002409  0.002409  0.002409
PC4-127---     0.016264  0.016264  0.021708  0.021708  0.019255  0.016187  0.015452  0.015467  0.014734  0.016187
 0.015535  0.018137  0.014932  0.016273  0.015535  0.014628  0.016339  0.020378  0.000798  0.000000  0.000798  0.
000798  0.001595  0.000806  0.000000  0.001603  0.000000  0.002411  0.003209  0.003199  0.003199  0.003199  0.
003206  0.003199  0.003199  0.003199  0.003199  0.003199  0.003199  0.003199  0.003199  0.003199  0.003199  0.
003199  0.003199  0.003199  0.003199  0.003199  0.003199  0.003199
PC4-137---     0.016264  0.016264  0.021708  0.021708  0.019255  0.016187  0.015452  0.015467  0.014734  0.016187
 0.015535  0.018137  0.014932  0.016273  0.015535  0.015425  0.017136  0.021175  0.000798  0.000798  0.000000  0.
000806  0.001595  0.001595  0.000798  0.001603  0.000798  0.002411  0.003209  0.002411  0.002411  0.002409  0.
003206  0.002409  0.002409  0.002409  0.002409  0.002409  0.002409  0.002409  0.002409  0.002409  0.002409  0.
002409  0.002409  0.002409  0.002401  0.002409  0.002409  0.002409
PC4-205---     0.016263  0.016263  0.021708  0.021708  0.019255  0.016187  0.015452  0.015467  0.014734  0.016187
 0.015534  0.018136  0.014932  0.016273  0.015534  0.014627  0.016338  0.020377  0.000799  0.000798  0.000806  0.
000000  0.002393  0.001596  0.000798  0.002393  0.000798  0.002402  0.002411  0.003199  0.003199  0.003199  0.
003201  0.003199  0.003199  0.003199  0.003199  0.003199  0.003199  0.003199  0.003199  0.003199  0.003199  0.
003199  0.003199  0.003199  0.003199  0.003199  0.003199  0.003199
civet007--     0.017421  0.017421  0.021708  0.021708  0.020850  0.017782  0.017047  0.017062  0.016329  0.017782
 0.017106  0.018136  0.016527  0.017868  0.017106  0.013830  0.015541  0.019580  0.001596  0.001595  0.001595  0.
002393  0.000000  0.000798  0.001595  0.000797  0.001595  0.004006  0.004804  0.004006  0.004006  0.004004  0.
004801  0.004004  0.004004  0.004004  0.004004  0.004004  0.004004  0.004004  0.004004  0.004004  0.004004  0.
004004  0.004004  0.004004  0.003207  0.004004  0.004004  0.004004
civet010--     0.016623  0.016623  0.021708  0.021708  0.020052  0.016984  0.016249  0.016264  0.015531  0.016984
 0.016308  0.018137  0.015729  0.017070  0.016308  0.013830  0.015541  0.019580  0.001596  0.000806  0.001595  0.
001596  0.000798  0.000000  0.000806  0.000798  0.000806  0.003209  0.004007  0.003209  0.003209  0.003207  0.
004004  0.003207  0.003207  0.003207  0.003207  0.003207  0.003207  0.003207  0.003207  0.003207  0.003207  0.
003207  0.003207  0.003207  0.002410  0.003207  0.003207  0.003207
civet020--     0.016264  0.016264  0.021708  0.021708  0.019255  0.016187  0.015452  0.015467  0.014734  0.016187
 0.015535  0.018137  0.014932  0.016273  0.015535  0.014628  0.016339  0.020378  0.000798  0.000000  0.000798  0.
000798  0.001595  0.000806  0.000000  0.001603  0.000000  0.002411  0.003209  0.003199  0.003199  0.003199  0.
003206  0.003199  0.003199  0.003199  0.003199  0.003199  0.003199  0.003199  0.003199  0.003199  0.003199  0.
003199  0.003199  0.003199  0.003199  0.003199  0.003199  0.003199
A022------     0.017421  0.017421  0.021708  0.021708  0.020850  0.017782  0.017047  0.017062  0.016329  0.017782
 0.017106  0.018137  0.016527  0.017868  0.017106  0.013830  0.015541  0.019580  0.001603  0.001603  0.001603  0.
002393  0.000797  0.000798  0.001603  0.000000  0.001603  0.004006  0.004804  0.004006  0.004006  0.004004  0.
004801  0.004004  0.004004  0.004004  0.004004  0.004004  0.004004  0.004004  0.004004  0.004004  0.004004  0.
004004  0.004004  0.004004  0.003207  0.004004  0.004004  0.004004
B039------     0.016264  0.016264  0.021708  0.021708  0.019255  0.016187  0.015452  0.015467  0.014734  0.016187
 0.015535  0.018137  0.014932  0.016273  0.015535  0.014628  0.016339  0.020378  0.000798  0.000000  0.000798  0.
000798  0.001595  0.000806  0.000000  0.001603  0.000000  0.002411  0.003209  0.003199  0.003199  0.003199  0.
003206  0.003199  0.003199  0.003199  0.003199  0.003199  0.003199  0.003199  0.003199  0.003199  0.003199  0.
003199  0.003199  0.003199  0.003199  0.003199  0.003199  0.003199
GD01------     0.016270  0.016270  0.020123  0.020123  0.018459  0.015391  0.014656  0.014671  0.013938  0.015391
 0.015541  0.018143  0.014136  0.015536  0.015541  0.016221  0.017932  0.021971  0.002411  0.002411  0.002411  0.
002402  0.004006  0.003209  0.002411  0.004006  0.002411  0.000000  0.000800  0.001595  0.001595  0.001596  0.
001595  0.001595  0.001595  0.001595  0.001595  0.001595  0.001596  0.001595  0.001595  0.001595  0.001595  0.
001595  0.001595  0.001595  0.001595  0.001595  0.001595  0.001595
GZ02------     0.017067  0.017067  0.020123  0.020123  0.018459  0.015391  0.014656  0.014671  0.013938  0.015391
```

```
 0.016338  0.018940  0.014136  0.016333  0.016338  0.016221  0.017932  0.021971  0.003209  0.003209  0.003209  0.
002411  0.004804  0.004007  0.003209  0.004804  0.003209  0.000800  0.000000  0.001595  0.001595  0.001596  0.
001596  0.001596  0.001596  0.001596  0.001596  0.001596  0.001596  0.001596  0.001596  0.001596  0.001596  0.
001596  0.001596  0.001596  0.001597  0.001596  0.001596  0.001596
BJ01------     0.017066  0.017066  0.020921  0.020921  0.018459  0.015391  0.014656  0.014671  0.013938  0.015391
 0.016337  0.018939  0.014136  0.016332  0.016337  0.015424  0.017135  0.021174  0.002411  0.003199  0.002411  0.
003199  0.004006  0.003209  0.003199  0.004006  0.003199  0.001595  0.001595  0.000000  0.000000  0.000799  0.
000799  0.000799  0.000799  0.000799  0.000799  0.000799  0.000799  0.000799  0.000799  0.000799  0.000799  0.
000799  0.000799  0.000799  0.000799  0.000799  0.000799  0.000799
CUHK-W1---     0.017066  0.017066  0.020921  0.020921  0.018459  0.015391  0.014656  0.014671  0.013938  0.015391
 0.016337  0.018939  0.014136  0.016332  0.016337  0.015424  0.017135  0.021174  0.002411  0.003199  0.002411  0.
003199  0.004006  0.003209  0.003199  0.004006  0.003199  0.001595  0.001595  0.000000  0.000000  0.000799  0.
000799  0.000799  0.000799  0.000799  0.000799  0.000799  0.000799  0.000799  0.000799  0.000799  0.000799  0.
000799  0.000799  0.000799  0.000799  0.000799  0.000799  0.000799
FRA-------     0.017864  0.017864  0.021719  0.021719  0.018459  0.015391  0.014656  0.015107  0.014374  0.015391
 0.017135  0.019737  0.014748  0.017130  0.017135  0.014627  0.016338  0.020377  0.002409  0.003199  0.002409  0.
003199  0.004004  0.003207  0.003199  0.004004  0.003199  0.001596  0.001596  0.000799  0.000799  0.000000  0.
000799  0.000799  0.000799  0.000799  0.000799  0.000799  0.000000  0.000799  0.000799  0.000799  0.000799  0.
000799  0.000799  0.000799  0.000800  0.000799  0.000799  0.000799
TOR2------     0.017066  0.017066  0.020920  0.020920  0.018459  0.015391  0.014656  0.014671  0.013938  0.015391
 0.016337  0.018939  0.014136  0.016332  0.016337  0.015012  0.016338  0.020377  0.003206  0.003206  0.003206  0.
003201  0.004801  0.004004  0.003206  0.004801  0.003206  0.001595  0.001596  0.000799  0.000799  0.000799  0.
000000  0.000798  0.000798  0.000798  0.000798  0.000798  0.000799  0.000798  0.000798  0.000798  0.000798  0.
000798  0.000798  0.000798  0.001594  0.000798  0.000798  0.000798
TaiwanTC1-     0.017066  0.017066  0.020920  0.020920  0.018459  0.015391  0.014656  0.014671  0.013938  0.015391
 0.016337  0.018939  0.014136  0.016332  0.016337  0.014627  0.016338  0.020377  0.002409  0.003199  0.002409  0.
003199  0.004004  0.003207  0.003199  0.004004  0.003199  0.001595  0.001596  0.000799  0.000799  0.000799  0.
000798  0.000000  0.000000  0.000000  0.000000  0.000000  0.000799  0.000000  0.000000  0.000000  0.000000  0.
000000  0.000000  0.000000  0.000797  0.000000  0.000000  0.000000
TaiwanTC2-     0.017066  0.017066  0.020920  0.020920  0.018459  0.015391  0.014656  0.014671  0.013938  0.015391
 0.016337  0.018939  0.014136  0.016332  0.016337  0.014627  0.016338  0.020377  0.002409  0.003199  0.002409  0.
003199  0.004004  0.003207  0.003199  0.004004  0.003199  0.001595  0.001596  0.000799  0.000799  0.000799  0.
000798  0.000000  0.000000  0.000000  0.000000  0.000000  0.000799  0.000000  0.000000  0.000000  0.000000  0.
000000  0.000000  0.000000  0.000797  0.000000  0.000000  0.000000
Urbani----     0.017066  0.017066  0.020920  0.020920  0.018459  0.015391  0.014656  0.014671  0.013938  0.015391
 0.016337  0.018939  0.014136  0.016332  0.016337  0.014627  0.016338  0.020377  0.002409  0.003199  0.002409  0.
003199  0.004004  0.003207  0.003199  0.004004  0.003199  0.001595  0.001596  0.000799  0.000799  0.000799  0.
000798  0.000000  0.000000  0.000000  0.000000  0.000000  0.000799  0.000000  0.000000  0.000000  0.000000  0.
000000  0.000000  0.000000  0.000797  0.000000  0.000000  0.000000
AS--------     0.017066  0.017066  0.020920  0.020920  0.018459  0.015391  0.014656  0.014671  0.013938  0.015391
 0.016337  0.018939  0.014136  0.016332  0.016337  0.014627  0.016338  0.020377  0.002409  0.003199  0.002409  0.
003199  0.004004  0.003207  0.003199  0.004004  0.003199  0.001595  0.001596  0.000799  0.000799  0.000799  0.
000798  0.000000  0.000000  0.000000  0.000000  0.000000  0.000799  0.000000  0.000000  0.000000  0.000000  0.
000000  0.000000  0.000000  0.000797  0.000000  0.000000  0.000000
CUHK-Su10-     0.017066  0.017066  0.020920  0.020920  0.018459  0.015391  0.014656  0.014671  0.013938  0.015391
 0.016337  0.018939  0.014136  0.016332  0.016337  0.014627  0.016338  0.020377  0.002409  0.003199  0.002409  0.
003199  0.004004  0.003207  0.003199  0.004004  0.003199  0.001595  0.001596  0.000799  0.000799  0.000799  0.
000798  0.000000  0.000000  0.000000  0.000000  0.000000  0.000799  0.000000  0.000000  0.000000  0.000000  0.
000000  0.000000  0.000000  0.000797  0.000000  0.000000  0.000000
Frankfurt1     0.017864  0.017864  0.021719  0.021719  0.018459  0.015391  0.014656  0.015107  0.014374  0.015391
 0.017135  0.019737  0.014748  0.017130  0.017135  0.014627  0.016338  0.020377  0.002409  0.003199  0.002409  0.
003199  0.004004  0.003207  0.003199  0.004004  0.003199  0.001596  0.001596  0.000799  0.000799  0.000000  0.
000799  0.000799  0.000799  0.000799  0.000799  0.000799  0.000000  0.000799  0.000799  0.000799  0.000799  0.
000799  0.000799  0.000799  0.000800  0.000799  0.000799  0.000799
CUHK-AG01-     0.017066  0.017066  0.020920  0.020920  0.018459  0.015391  0.014656  0.014671  0.013938  0.015391
 0.016337  0.018939  0.014136  0.016332  0.016337  0.014627  0.016338  0.020377  0.002409  0.003199  0.002409  0.
003199  0.004004  0.003207  0.003199  0.004004  0.003199  0.001595  0.001596  0.000799  0.000799  0.000799  0.
000798  0.000000  0.000000  0.000000  0.000000  0.000000  0.000799  0.000000  0.000000  0.000000  0.000000  0.
000000  0.000000  0.000000  0.000797  0.000000  0.000000  0.000000
CUHK-AG02-     0.017066  0.017066  0.020920  0.020920  0.018459  0.015391  0.014656  0.014671  0.013938  0.015391
 0.016337  0.018939  0.014136  0.016332  0.016337  0.014627  0.016338  0.020377  0.002409  0.003199  0.002409  0.
003199  0.004004  0.003207  0.003199  0.004004  0.003199  0.001595  0.001596  0.000799  0.000799  0.000799  0.
```

```
000798  0.000000  0.000000  0.000000  0.000000  0.000000  0.000799  0.000000  0.000000  0.000000  0.000000  0.
000000  0.000000  0.000000  0.000797  0.000000  0.000000  0.000000
TW1-------     0.017066  0.017066  0.020920  0.020920  0.018459  0.015391  0.014656  0.014671  0.013938  0.015391
 0.016337  0.018939  0.014136  0.016332  0.016337  0.014627  0.016338  0.020377  0.002409  0.003199  0.002409  0.
003199  0.004004  0.003207  0.003199  0.004004  0.003199  0.001595  0.001596  0.000799  0.000799  0.000799  0.
000798  0.000000  0.000000  0.000000  0.000000  0.000000  0.000799  0.000000  0.000000  0.000000  0.000000  0.
000000  0.000000  0.000000  0.000797  0.000000  0.000000  0.000000
TW2-------     0.017066  0.017066  0.020920  0.020920  0.018459  0.015391  0.014656  0.014671  0.013938  0.015391
 0.016337  0.018939  0.014136  0.016332  0.016337  0.014627  0.016338  0.020377  0.002409  0.003199  0.002409  0.
003199  0.004004  0.003207  0.003199  0.004004  0.003199  0.001595  0.001596  0.000799  0.000799  0.000799  0.
000798  0.000000  0.000000  0.000000  0.000000  0.000000  0.000799  0.000000  0.000000  0.000000  0.000000  0.
000000  0.000000  0.000000  0.000797  0.000000  0.000000  0.000000
TWH-------     0.017066  0.017066  0.020920  0.020920  0.018459  0.015391  0.014656  0.014671  0.013938  0.015391
 0.016337  0.018939  0.014136  0.016332  0.016337  0.014627  0.016338  0.020377  0.002409  0.003199  0.002409  0.
003199  0.004004  0.003207  0.003199  0.004004  0.003199  0.001595  0.001596  0.000799  0.000799  0.000799  0.
000798  0.000000  0.000000  0.000000  0.000000  0.000000  0.000799  0.000000  0.000000  0.000000  0.000000  0.
000000  0.000000  0.000000  0.000797  0.000000  0.000000  0.000000
TWJ-------     0.017066  0.017066  0.020920  0.020920  0.018459  0.015391  0.014656  0.014671  0.013938  0.015391
 0.016337  0.018939  0.014136  0.016332  0.016337  0.014627  0.016338  0.020377  0.002409  0.003199  0.002409  0.
003199  0.004004  0.003207  0.003199  0.004004  0.003199  0.001595  0.001596  0.000799  0.000799  0.000799  0.
000798  0.000000  0.000000  0.000000  0.000000  0.000000  0.000799  0.000000  0.000000  0.000000  0.000000  0.
000000  0.000000  0.000000  0.000797  0.000000  0.000000  0.000000
HSR1------     0.017066  0.017066  0.020920  0.020920  0.018459  0.015391  0.014656  0.014671  0.013938  0.015391
 0.016337  0.018939  0.014136  0.016332  0.016337  0.014627  0.016338  0.020377  0.002409  0.003199  0.002409  0.
003199  0.004004  0.003207  0.003199  0.004004  0.003199  0.001595  0.001596  0.000799  0.000799  0.000799  0.
000798  0.000000  0.000000  0.000000  0.000000  0.000000  0.000799  0.000000  0.000000  0.000000  0.000000  0.
000000  0.000000  0.000000  0.000797  0.000000  0.000000  0.000000
Sino1-11--     0.017066  0.017066  0.020919  0.020919  0.018459  0.015391  0.014656  0.014671  0.013938  0.015391
 0.016337  0.018939  0.014136  0.016332  0.016337  0.014627  0.016338  0.020377  0.002402  0.003199  0.002401  0.
003199  0.003207  0.002410  0.003199  0.003207  0.003199  0.001595  0.001597  0.000799  0.000799  0.000800  0.
001594  0.000797  0.000797  0.000797  0.000797  0.000797  0.000800  0.000797  0.000797  0.000797  0.000797  0.
000797  0.000797  0.000797  0.000000  0.000797  0.000797  0.000797
Sino3-11--     0.017066  0.017066  0.020920  0.020920  0.018459  0.015391  0.014656  0.014671  0.013938  0.015391
 0.016337  0.018939  0.014136  0.016332  0.016337  0.014627  0.016338  0.020377  0.002409  0.003199  0.002409  0.
003199  0.004004  0.003207  0.003199  0.004004  0.003199  0.001595  0.001596  0.000799  0.000799  0.000799  0.
000798  0.000000  0.000000  0.000000  0.000000  0.000000  0.000799  0.000000  0.000000  0.000000  0.000000  0.
000000  0.000000  0.000000  0.000797  0.000000  0.000000  0.000000
PUMC01----     0.017066  0.017066  0.020920  0.020920  0.018459  0.015391  0.014656  0.014671  0.013938  0.015391
 0.016337  0.018939  0.014136  0.016332  0.016337  0.014627  0.016338  0.020377  0.002409  0.003199  0.002409  0.
003199  0.004004  0.003207  0.003199  0.004004  0.003199  0.001595  0.001596  0.000799  0.000799  0.000799  0.
000798  0.000000  0.000000  0.000000  0.000000  0.000000  0.000799  0.000000  0.000000  0.000000  0.000000  0.
000000  0.000000  0.000000  0.000797  0.000000  0.000000  0.000000
PUMC02----     0.017066  0.017066  0.020920  0.020920  0.018459  0.015391  0.014656  0.014671  0.013938  0.015391
 0.016337  0.018939  0.014136  0.016332  0.016337  0.014627  0.016338  0.020377  0.002409  0.003199  0.002409  0.
003199  0.004004  0.003207  0.003199  0.004004  0.003199  0.001595  0.001596  0.000799  0.000799  0.000799  0.
000798  0.000000  0.000000  0.000000  0.000000  0.000000  0.000799  0.000000  0.000000  0.000000  0.000000  0.
000000  0.000000  0.000000  0.000797  0.000000  0.000000  0.000000
```

6. beta-globin protein sequences.

```
    50
Human-----     0.000000  0.037694  0.021382  0.021565  0.020272  0.045144  0.045133  0.045163  0.028062  0.038259
 0.044229  0.021450  0.033932  0.026718  0.045162  0.020225  0.021450  0.000000  0.020749  0.051045  0.021401  0.
020226  0.045169  0.021450  0.043908  0.045255  0.021401  0.027322  0.027303  0.044303  0.013471  0.006989  0.
037977  0.020375  0.027115  0.013463  0.020565  0.014961  0.021356  0.038589  0.037726  0.027246  0.045171  0.
045147  0.034388  0.057247  0.044499  0.027111  0.061905  0.013702
Goshawk---     0.037694  0.000000  0.049319  0.049348  0.041737  0.021343  0.020793  0.020833  0.030983  0.013892
 0.013880  0.049381  0.024757  0.042684  0.020795  0.042621  0.049381  0.037694  0.042299  0.033130  0.056260  0.
033149  0.027828  0.049381  0.033317  0.021064  0.056260  0.035851  0.035863  0.028101  0.042353  0.037739  0.
```

```
051163  0.028643  0.042616  0.035417  0.027029  0.049257  0.049412  0.027911  0.026668  0.034582  0.027862  0.
014089  0.043732  0.044039  0.021077  0.042638  0.062700  0.034301
Lesser_pan     0.021382  0.049319  0.000000  0.007367  0.020424  0.041926  0.041915  0.041945  0.024844  0.049054
 0.049256  0.007068  0.032602  0.021217  0.041944  0.021782  0.007068  0.021382  0.020753  0.049420  0.006941  0.
034100  0.041951  0.007068  0.041045  0.042037  0.006941  0.020899  0.020977  0.041454  0.013856  0.014393  0.
043199  0.028500  0.020769  0.020849  0.034501  0.013877  0.007024  0.040616  0.049408  0.021719  0.041953  0.
041929  0.021024  0.056649  0.041281  0.020743  0.058687  0.020836
Gaint_pand     0.021565  0.049348  0.007367  0.000000  0.015190  0.045073  0.045062  0.045092  0.027991  0.049083
 0.049285  0.013596  0.032605  0.021246  0.045091  0.021811  0.013596  0.021565  0.023886  0.050974  0.010003  0.
034129  0.045098  0.013596  0.043837  0.045184  0.010003  0.020878  0.021053  0.044232  0.016989  0.017296  0.
043199  0.028683  0.020952  0.021032  0.034530  0.013786  0.009686  0.040645  0.049591  0.024866  0.045100  0.
045076  0.027598  0.056898  0.044428  0.020926  0.061834  0.021019
Sheep-----     0.020272  0.041737  0.020424  0.015190  0.000000  0.049187  0.049176  0.049206  0.032105  0.042302
 0.048272  0.026653  0.031959  0.014274  0.049205  0.014263  0.026653  0.020272  0.021443  0.055088  0.021754  0.
019287  0.049212  0.026653  0.047951  0.049298  0.021754  0.026847  0.026867  0.048346  0.015592  0.020274  0.
043199  0.022179  0.019154  0.015604  0.019688  0.021887  0.014981  0.042632  0.037721  0.028980  0.049214  0.
049190  0.021357  0.057192  0.048542  0.014595  0.065948  0.015594
Duck------     0.045144  0.021343  0.041926  0.045073  0.049187  0.000000  0.007003  0.014186  0.038119  0.020937
 0.020790  0.035445  0.032207  0.050134  0.007030  0.050071  0.035445  0.045144  0.040431  0.033580  0.035473  0.
040599  0.014071  0.035445  0.028165  0.027974  0.035473  0.036317  0.035863  0.028181  0.041762  0.045189  0.
045032  0.037860  0.040528  0.041744  0.037382  0.041978  0.035843  0.034821  0.027834  0.020595  0.021174  0.
014124  0.051182  0.030223  0.014013  0.040547  0.041913  0.041751
Mallard---     0.045133  0.020793  0.041915  0.045062  0.049176  0.007003  0.000000  0.014192  0.038124  0.020528
 0.020796  0.035434  0.032196  0.050123  0.013721  0.050060  0.035434  0.045133  0.040420  0.033570  0.035467  0.
040588  0.014077  0.035434  0.027019  0.027980  0.035467  0.035886  0.035884  0.027703  0.041751  0.045178  0.
045113  0.037866  0.040517  0.041733  0.037383  0.041967  0.035842  0.034827  0.027840  0.020599  0.021180  0.
014018  0.051171  0.030219  0.013981  0.040536  0.041907  0.041740
Goose-----     0.045163  0.020833  0.041945  0.045092  0.049206  0.014186  0.014192  0.000000  0.038139  0.020568
 0.020770  0.036369  0.032226  0.050153  0.013742  0.050090  0.036369  0.045163  0.040450  0.033543  0.035857  0.
040618  0.007106  0.036369  0.027032  0.013788  0.035857  0.035403  0.035401  0.021302  0.041781  0.045208  0.
045092  0.037877  0.040547  0.041763  0.036924  0.041997  0.036321  0.026660  0.027081  0.020589  0.007029  0.
007061  0.051201  0.030229  0.013882  0.040566  0.041867  0.041770
Rat-------     0.028062  0.030983  0.024844  0.027991  0.032105  0.038119  0.038124  0.038139  0.000000  0.044875
 0.037772  0.021615  0.025278  0.033052  0.038115  0.032989  0.021615  0.028062  0.041263  0.042748  0.028494  0.
027987  0.038122  0.021615  0.045834  0.031064  0.028494  0.035002  0.041814  0.046243  0.027289  0.028107  0.
048120  0.034367  0.034853  0.024662  0.034205  0.024896  0.021646  0.020555  0.035513  0.028797  0.038140  0.
045072  0.034100  0.053763  0.024257  0.034873  0.043665  0.024669
Penguin---     0.038259  0.013892  0.049054  0.049083  0.042302  0.020937  0.020528  0.020568  0.044875  0.000000
 0.013517  0.049116  0.025322  0.043249  0.020530  0.043186  0.049116  0.038259  0.042034  0.026226  0.055995  0.
040384  0.027563  0.049116  0.028324  0.020542  0.055995  0.035586  0.035598  0.028340  0.042088  0.038304  0.
050898  0.033577  0.046999  0.040003  0.031019  0.048992  0.049147  0.033396  0.021093  0.034317  0.027597  0.
013507  0.044297  0.037227  0.020812  0.042373  0.062435  0.040007
Swift-----     0.044229  0.013880  0.049256  0.049285  0.048272  0.020790  0.020796  0.020770  0.037772  0.013517
 0.000000  0.049318  0.031292  0.049219  0.020732  0.049156  0.049318  0.044229  0.042236  0.027867  0.056197  0.
039684  0.027765  0.049318  0.033322  0.020744  0.056197  0.035788  0.035800  0.021537  0.042290  0.044274  0.
051100  0.035698  0.042553  0.040829  0.036540  0.049194  0.049349  0.026621  0.026404  0.034519  0.027799  0.
013709  0.050267  0.030786  0.021014  0.042575  0.062637  0.040836
Coyote----     0.021450  0.049381  0.007068  0.013596  0.026653  0.035445  0.035434  0.036369  0.021615  0.049116
 0.049318  0.000000  0.032847  0.021279  0.035858  0.021844  0.000000  0.021450  0.027494  0.049488  0.006879  0.
034162  0.036360  0.000000  0.034687  0.041157  0.006879  0.027128  0.028045  0.036136  0.019816  0.020421  0.
040293  0.028568  0.021084  0.020917  0.034563  0.013831  0.013057  0.040678  0.049476  0.021429  0.036780  0.
035838  0.021092  0.058785  0.034800  0.021104  0.052206  0.020904
Catfish---     0.033932  0.024757  0.032602  0.032605  0.031959  0.032207  0.032196  0.032226  0.025278  0.025322
 0.031292  0.032847  0.000000  0.032557  0.032225  0.032557  0.032847  0.033932  0.037901  0.038108  0.032334  0.
032664  0.032232  0.032847  0.030971  0.032318  0.032334  0.032370  0.038452  0.031366  0.032766  0.033631  0.
040505  0.031873  0.032016  0.033226  0.033379  0.032385  0.032671  0.029283  0.029735  0.028678  0.032234  0.
032210  0.032180  0.040173  0.031562  0.032024  0.048968  0.033225
Bison-----     0.026718  0.042684  0.021217  0.021246  0.014274  0.050134  0.050123  0.050153  0.033052  0.043249
 0.049219  0.021279  0.032557  0.000000  0.050152  0.006943  0.021279  0.026718  0.021467  0.056035  0.028158  0.
020625  0.050159  0.021279  0.048898  0.050245  0.028158  0.026802  0.026822  0.049293  0.019967  0.026720  0.
032516  0.022134  0.021106  0.019894  0.016047  0.021911  0.021310  0.043579  0.038773  0.029927  0.050161  0.
050137  0.028379  0.058534  0.049489  0.021102  0.066895  0.019940
```

```
Swan------     0.045162  0.020795  0.041944  0.045091  0.049205  0.007030  0.013721  0.013742  0.038115  0.020530
 0.020732  0.035858  0.032225  0.050152  0.000000  0.050089  0.035858  0.045162  0.041209  0.033572  0.035465  0.
040617  0.013751  0.035858  0.035001  0.020944  0.035465  0.035428  0.041760  0.035017  0.041780  0.045207  0.
045085  0.037874  0.040546  0.041762  0.036948  0.041996  0.036294  0.027791  0.027110  0.020582  0.014144  0.
014011  0.051200  0.030236  0.014014  0.040565  0.041905  0.041769
Buffalo---     0.020225  0.042621  0.021782  0.021811  0.014263  0.050071  0.050060  0.050090  0.032989  0.043186
 0.049156  0.021844  0.032557  0.006943  0.050089  0.000000  0.021844  0.020225  0.021559  0.055972  0.028723  0.
020676  0.050096  0.021844  0.048835  0.050182  0.028723  0.026670  0.026690  0.049230  0.015708  0.020227  0.
032516  0.022002  0.021005  0.015720  0.015984  0.022003  0.021875  0.043516  0.038773  0.029864  0.050098  0.
050074  0.028278  0.058037  0.049426  0.021001  0.066832  0.015710
Dog-------     0.021450  0.049381  0.007068  0.013596  0.026653  0.035445  0.035434  0.036369  0.021615  0.049116
 0.049318  0.000000  0.032847  0.021279  0.035858  0.021844  0.000000  0.021450  0.027494  0.049488  0.006879  0.
034162  0.036360  0.000000  0.034687  0.041157  0.006879  0.027128  0.028045  0.036136  0.019816  0.020421  0.
040293  0.028568  0.021084  0.020917  0.034563  0.013831  0.013057  0.040678  0.049476  0.021429  0.036780  0.
035838  0.021092  0.058785  0.034800  0.021104  0.052206  0.020904
Chimpanzee     0.000000  0.037694  0.021382  0.021565  0.020272  0.045144  0.045133  0.045163  0.028062  0.038259
 0.044229  0.021450  0.033932  0.026718  0.045162  0.020225  0.021450  0.000000  0.020749  0.051045  0.021401  0.
020226  0.045169  0.021450  0.043908  0.045255  0.021401  0.027322  0.027303  0.044303  0.013471  0.006989  0.
037977  0.020375  0.027115  0.013463  0.020565  0.014961  0.021356  0.038589  0.037726  0.027246  0.045171  0.
045147  0.034388  0.057247  0.044499  0.027111  0.061905  0.013702
Dolphin---     0.020749  0.042299  0.020753  0.023886  0.021443  0.040431  0.040420  0.040450  0.041263  0.042034
 0.042236  0.027494  0.037901  0.021467  0.041209  0.021559  0.027494  0.020749  0.000000  0.046332  0.027468  0.
027080  0.040456  0.027494  0.041420  0.040542  0.027468  0.021139  0.021146  0.041829  0.020372  0.020752  0.
037614  0.014401  0.027135  0.020398  0.027481  0.020778  0.027450  0.041299  0.048238  0.026911  0.040458  0.
040658  0.034408  0.055470  0.039786  0.027131  0.057192  0.020378
Goldfish--     0.051045  0.033130  0.049420  0.050974  0.055088  0.033580  0.033570  0.033543  0.042748  0.026226
 0.027867  0.049488  0.038108  0.056035  0.033572  0.055972  0.049488  0.051045  0.046332  0.000000  0.049439  0.
046634  0.033550  0.049488  0.047273  0.027948  0.049439  0.054419  0.054439  0.040100  0.047663  0.051090  0.
066015  0.049751  0.046429  0.047645  0.042583  0.047879  0.049394  0.042790  0.031493  0.033185  0.033547  0.
033439  0.057083  0.034244  0.033098  0.046448  0.047173  0.047652
Polar_bear     0.021401  0.056260  0.006941  0.010003  0.021754  0.035473  0.035467  0.035857  0.028494  0.055995
 0.056197  0.006879  0.032334  0.028158  0.035465  0.028723  0.006879  0.021401  0.027468  0.049439  0.000000  0.
041041  0.035857  0.006879  0.041283  0.040119  0.000000  0.021032  0.027844  0.041692  0.020571  0.020882  0.
044471  0.028519  0.020883  0.020868  0.041442  0.013871  0.006912  0.047557  0.049427  0.021678  0.035855  0.
042488  0.021043  0.057595  0.035183  0.020903  0.051831  0.027679
Rhinoceros     0.020226  0.033149  0.034100  0.034129  0.019287  0.040599  0.040588  0.040618  0.027987  0.040384
 0.039684  0.034162  0.032664  0.020625  0.040617  0.020676  0.034162  0.020226  0.027080  0.046634  0.041041  0.
000000  0.040624  0.034162  0.041343  0.040710  0.041041  0.020999  0.020998  0.041752  0.027134  0.020228  0.
044361  0.014564  0.027397  0.020198  0.020548  0.034038  0.034193  0.040940  0.040172  0.020865  0.040626  0.
040602  0.026737  0.057292  0.039954  0.027419  0.057360  0.013448
Chiken----     0.045169  0.027828  0.041951  0.045098  0.049212  0.014071  0.014077  0.007106  0.038122  0.027563
 0.027765  0.036360  0.032232  0.050159  0.013751  0.050096  0.036360  0.045169  0.040456  0.033550  0.035857  0.
040624  0.000000  0.036360  0.027048  0.013903  0.035857  0.035881  0.035879  0.021266  0.041787  0.045214  0.
045066  0.037873  0.040553  0.041769  0.036883  0.042003  0.036321  0.026643  0.027088  0.020577  0.007106  0.
014056  0.051207  0.030241  0.013865  0.040572  0.034872  0.041776
Wolf------     0.021450  0.049381  0.007068  0.013596  0.026653  0.035445  0.035434  0.036369  0.021615  0.049116
 0.049318  0.000000  0.032847  0.021279  0.035858  0.021844  0.000000  0.021450  0.027494  0.049488  0.006879  0.
034162  0.036360  0.000000  0.034687  0.041157  0.006879  0.027128  0.028045  0.036136  0.019816  0.020421  0.
040293  0.028568  0.021084  0.020917  0.034563  0.013831  0.013057  0.040678  0.049476  0.021429  0.036780  0.
035838  0.021092  0.058785  0.034800  0.021104  0.052206  0.020904
Turtle----     0.043908  0.033317  0.041045  0.043837  0.047951  0.028165  0.027019  0.027032  0.045834  0.028324
 0.033322  0.034687  0.030971  0.048898  0.035001  0.048835  0.034687  0.043908  0.041420  0.047273  0.041283  0.
041343  0.027048  0.034687  0.000000  0.028105  0.041283  0.032769  0.032786  0.026903  0.040953  0.043953  0.
038059  0.035617  0.047958  0.040962  0.032851  0.048018  0.041005  0.042195  0.047865  0.033322  0.028144  0.
027010  0.049946  0.046482  0.026851  0.041605  0.045274  0.040966
Pigeon----     0.045255  0.021064  0.042037  0.045184  0.049298  0.027974  0.027980  0.013788  0.031064  0.020542
 0.020744  0.041157  0.032318  0.050245  0.020944  0.050182  0.041157  0.045255  0.040542  0.027948  0.040119  0.
040710  0.013903  0.041157  0.028105  0.000000  0.040119  0.040114  0.039622  0.028121  0.041873  0.045300  0.
038638  0.041757  0.040639  0.041855  0.041350  0.042089  0.040652  0.021399  0.027365  0.026934  0.013482  0.
014008  0.051293  0.023884  0.013999  0.040658  0.041893  0.041862
Black_bear     0.021401  0.056260  0.006941  0.010003  0.021754  0.035473  0.035467  0.035857  0.028494  0.055995
 0.056197  0.006879  0.032334  0.028158  0.035465  0.028723  0.006879  0.021401  0.027468  0.049439  0.000000  0.
```

```
041041  0.035857  0.006879  0.041283  0.040119  0.000000  0.021032  0.027844  0.041692  0.020571  0.020882  0.
044471  0.028519  0.020883  0.020868  0.041442  0.013871  0.006912  0.047557  0.049427  0.021678  0.035855  0.
042488  0.021043  0.057595  0.035183  0.020903  0.051831  0.027679
Asiatic_el     0.027322  0.035851  0.020899  0.020878  0.026847  0.036317  0.035886  0.035403  0.035002  0.035586
 0.035788  0.027128  0.032370  0.026802  0.035428  0.026670  0.027128  0.027322  0.021139  0.054419  0.021032  0.
020999  0.035881  0.027128  0.032769  0.040114  0.021032  0.000000  0.006812  0.036224  0.020865  0.027324  0.
049463  0.020782  0.034066  0.020529  0.021136  0.021013  0.021189  0.041806  0.047957  0.034312  0.035401  0.
034915  0.041339  0.056874  0.033360  0.034062  0.050766  0.020653
African_el     0.027303  0.035863  0.020977  0.021053  0.026867  0.035863  0.035884  0.035401  0.041814  0.035598
 0.035800  0.028045  0.038452  0.026822  0.041760  0.026690  0.028045  0.027303  0.021146  0.054439  0.027844  0.
020998  0.035879  0.028045  0.032786  0.039622  0.027844  0.006812  0.000000  0.036221  0.020923  0.027305  0.
049483  0.020781  0.034068  0.020949  0.021143  0.021329  0.028001  0.041850  0.048789  0.034311  0.034963  0.
034912  0.041341  0.056167  0.033377  0.034064  0.050783  0.020929
Tortoise--     0.044303  0.028101  0.041454  0.044232  0.048346  0.028181  0.027703  0.021302  0.046243  0.028340
 0.021537  0.036136  0.031366  0.049293  0.035017  0.049230  0.036136  0.044303  0.041829  0.040100  0.041692  0.
041752  0.021266  0.036136  0.026903  0.028121  0.041692  0.036224  0.036221  0.000000  0.041362  0.044348  0.
040740  0.035822  0.048367  0.041371  0.037274  0.048427  0.041414  0.042211  0.047941  0.022458  0.028160  0.
021108  0.050341  0.046498  0.021986  0.041482  0.045290  0.041375
Grivet----     0.013471  0.042353  0.013856  0.016989  0.015592  0.041762  0.041751  0.041781  0.027289  0.042088
 0.042290  0.019816  0.032766  0.019967  0.041780  0.015708  0.019816  0.013471  0.020372  0.047663  0.020571  0.
027134  0.041787  0.019816  0.040953  0.041873  0.020571  0.020865  0.020923  0.041362  0.000000  0.013466  0.
033803  0.021809  0.027220  0.007024  0.027535  0.014623  0.013825  0.035207  0.037689  0.022127  0.041789  0.
041765  0.034493  0.055976  0.041117  0.027216  0.058523  0.013807
Gorilla---     0.006989  0.037739  0.014393  0.017296  0.020274  0.045189  0.045178  0.045208  0.028107  0.038304
 0.044274  0.020421  0.033631  0.026720  0.045207  0.020227  0.020421  0.006989  0.020752  0.051090  0.020882  0.
020228  0.045214  0.020421  0.043953  0.045300  0.020882  0.027324  0.027305  0.044348  0.013466  0.000000  0.
033566  0.022029  0.027106  0.013458  0.020560  0.014971  0.020517  0.038634  0.037241  0.027241  0.045216  0.
045192  0.034379  0.056516  0.044544  0.027102  0.061950  0.013693
Shark-----     0.037977  0.051163  0.043199  0.043199  0.043199  0.045032  0.045113  0.045092  0.048120  0.050898
 0.051100  0.040293  0.040505  0.032516  0.045085  0.032516  0.040293  0.037977  0.037614  0.066015  0.044471  0.
044361  0.045066  0.040293  0.038059  0.038638  0.044471  0.049463  0.049483  0.040740  0.033803  0.033566  0.
000000  0.045095  0.048543  0.037444  0.037627  0.040873  0.039870  0.043903  0.066003  0.037956  0.045045  0.
045020  0.054342  0.041332  0.044961  0.048560  0.065933  0.037431
Hippopotam     0.020375  0.028643  0.028500  0.028683  0.022179  0.037860  0.037866  0.037877  0.034367  0.033577
 0.035698  0.028568  0.031873  0.022134  0.037874  0.022002  0.028568  0.020375  0.014401  0.049751  0.028519  0.
014564  0.037873  0.028568  0.035617  0.041757  0.028519  0.020782  0.020781  0.035822  0.021809  0.022029  0.
045095  0.000000  0.014385  0.014799  0.021231  0.021613  0.028474  0.034512  0.043289  0.021898  0.037868  0.
037342  0.021272  0.057276  0.032838  0.014384  0.050244  0.014802
Horse-----     0.027115  0.042616  0.020769  0.020952  0.019154  0.040528  0.040517  0.040547  0.034853  0.046999
 0.042553  0.021084  0.032016  0.021106  0.040546  0.021005  0.021084  0.027115  0.027135  0.046429  0.020883  0.
027397  0.040553  0.021084  0.047958  0.040639  0.020883  0.034066  0.034068  0.048367  0.027220  0.027106  0.
048543  0.014385  0.000000  0.020347  0.027798  0.020262  0.021040  0.034889  0.041828  0.027252  0.040555  0.
047196  0.014157  0.054887  0.039883  0.006885  0.057289  0.014035
Gibbon----     0.013463  0.035417  0.020849  0.021032  0.015604  0.041744  0.041733  0.041763  0.024662  0.040003
 0.040829  0.020917  0.033226  0.019894  0.041762  0.015720  0.020917  0.013463  0.020398  0.047645  0.020868  0.
020198  0.041769  0.020917  0.040962  0.041855  0.020868  0.020529  0.020949  0.041371  0.007024  0.013458  0.
037444  0.014799  0.020347  0.000000  0.020599  0.014650  0.020823  0.035189  0.038186  0.022441  0.041771  0.
041747  0.027620  0.056708  0.041099  0.020343  0.058505  0.006934
Whale-----     0.020565  0.027029  0.034501  0.034530  0.019688  0.037382  0.037383  0.036924  0.034205  0.031019
 0.036540  0.034563  0.033379  0.016047  0.036948  0.015984  0.034563  0.020565  0.027481  0.042583  0.041442  0.
020548  0.036883  0.034563  0.032851  0.041350  0.041442  0.021136  0.021143  0.037274  0.027535  0.020560  0.
037627  0.021231  0.027798  0.020599  0.000000  0.034439  0.034594  0.034546  0.042139  0.027096  0.036421  0.
036835  0.034475  0.058170  0.033442  0.027820  0.050848  0.020171
Bat-------     0.014961  0.049257  0.013877  0.013786  0.021887  0.041978  0.041967  0.041997  0.024896  0.048992
 0.049194  0.013831  0.032385  0.021911  0.041996  0.022003  0.013831  0.014961  0.020778  0.047879  0.013871  0.
034038  0.042003  0.013831  0.048018  0.042089  0.013871  0.021013  0.021329  0.048427  0.014623  0.014971  0.
040873  0.021613  0.020262  0.014650  0.034439  0.000000  0.013881  0.040554  0.042521  0.025969  0.042005  0.
047256  0.027535  0.056790  0.041333  0.020258  0.058739  0.020676
Red_fox---     0.021356  0.049412  0.007024  0.009686  0.014981  0.035843  0.035842  0.036321  0.021646  0.049147
 0.049349  0.013057  0.032671  0.021310  0.036294  0.021875  0.013057  0.021356  0.027450  0.049394  0.006912  0.
034193  0.036321  0.013057  0.041005  0.040652  0.006912  0.021189  0.028001  0.041414  0.013825  0.020517  0.
039870  0.028474  0.021040  0.020823  0.034594  0.013881  0.000000  0.040709  0.049382  0.021335  0.036319  0.
```

```
040243  0.027564  0.058085  0.034742  0.021060  0.052148  0.020831
Marmot----     0.038589  0.027911  0.040616  0.040645  0.042632  0.034821  0.034827  0.026660  0.020555  0.033396
 0.026621  0.040678  0.029283  0.043579  0.027791  0.043516  0.040678  0.038589  0.041299  0.042790  0.047557  0.
040940  0.026643  0.040678  0.042195  0.021399  0.047557  0.041806  0.041850  0.042211  0.035207  0.038634  0.
043903  0.034512  0.034889  0.035189  0.034546  0.040554  0.040709  0.000000  0.042778  0.027044  0.026661  0.
033593  0.044627  0.045283  0.026710  0.034909  0.053997  0.035196
Salmon----     0.037726  0.026668  0.049408  0.049591  0.037721  0.027834  0.027840  0.027081  0.035513  0.021093
 0.026404  0.049476  0.029735  0.038773  0.027110  0.038773  0.049476  0.037726  0.048238  0.031493  0.049427  0.
040172  0.027088  0.049476  0.047865  0.027365  0.049427  0.047957  0.048789  0.047941  0.037689  0.037241  0.
066003  0.043289  0.041828  0.038186  0.042139  0.042521  0.049382  0.042778  0.000000  0.028047  0.027085  0.
026977  0.041178  0.033558  0.026938  0.041848  0.047641  0.038180
Sparrow---     0.027246  0.034582  0.021719  0.024866  0.028980  0.020595  0.020599  0.020589  0.028797  0.034317
 0.034519  0.021429  0.028678  0.029927  0.020582  0.029864  0.021429  0.027246  0.026911  0.033185  0.021678  0.
020865  0.020577  0.021429  0.033322  0.026934  0.021678  0.034312  0.034311  0.022458  0.022127  0.027241  0.
037956  0.021898  0.027252  0.022441  0.027096  0.025969  0.021335  0.027044  0.028047  0.000000  0.020572  0.
021287  0.034525  0.050818  0.019562  0.027248  0.040720  0.022171
Pheasant--     0.045171  0.027862  0.041953  0.045100  0.049214  0.021174  0.021180  0.007029  0.038140  0.027597
 0.027799  0.036780  0.032234  0.050161  0.014144  0.050098  0.036780  0.045171  0.040458  0.033547  0.035855  0.
040626  0.007106  0.036780  0.028144  0.013482  0.035855  0.035401  0.034963  0.028160  0.041789  0.045216  0.
045045  0.037868  0.040555  0.041771  0.036421  0.042005  0.036319  0.026661  0.027085  0.020572  0.000000  0.
014090  0.051209  0.030246  0.013883  0.040574  0.034838  0.041778
Flamingo--     0.045147  0.014089  0.041929  0.045076  0.049190  0.014124  0.014018  0.007061  0.045072  0.013507
 0.013709  0.035838  0.032210  0.050137  0.014011  0.050074  0.035838  0.045147  0.040658  0.033439  0.042488  0.
040602  0.014056  0.035838  0.027010  0.014008  0.042488  0.034915  0.034912  0.021108  0.041765  0.045192  0.
045020  0.037342  0.047196  0.041747  0.036835  0.047256  0.040243  0.033593  0.026977  0.021287  0.014090  0.
000000  0.051185  0.030265  0.020815  0.040550  0.048928  0.041754
Pig-------     0.034388  0.043732  0.021024  0.027598  0.021357  0.051182  0.051171  0.051201  0.034100  0.044297
 0.050267  0.021092  0.032180  0.028379  0.051200  0.028278  0.021092  0.034388  0.034408  0.057083  0.021043  0.
026737  0.051207  0.021092  0.049946  0.051293  0.021043  0.041339  0.041341  0.050341  0.034493  0.034379  0.
054342  0.021272  0.014157  0.027620  0.034475  0.027535  0.027564  0.044627  0.041178  0.034525  0.051209  0.
051185  0.000000  0.059148  0.050537  0.014177  0.067943  0.020686
Dragonfish     0.057247  0.044039  0.056649  0.056898  0.057192  0.030223  0.030219  0.030229  0.053763  0.037227
 0.030786  0.058785  0.040173  0.058534  0.030236  0.058037  0.058785  0.057247  0.055470  0.034244  0.057595  0.
057292  0.030241  0.058785  0.046482  0.023884  0.057595  0.056874  0.056167  0.046498  0.055976  0.056516  0.
041332  0.057276  0.054887  0.056708  0.058170  0.056790  0.058085  0.045283  0.033558  0.050818  0.030246  0.
030265  0.059148  0.000000  0.037292  0.054901  0.040836  0.056700
Parakeet--     0.044499  0.021077  0.041281  0.044428  0.048542  0.014013  0.013981  0.013882  0.024257  0.020812
 0.021014  0.034800  0.031562  0.049489  0.014014  0.049426  0.034800  0.044499  0.039786  0.033098  0.035183  0.
039954  0.013865  0.034800  0.026851  0.013999  0.035183  0.033360  0.033377  0.021986  0.041117  0.044544  0.
044961  0.032838  0.039883  0.041099  0.033442  0.041333  0.034742  0.026710  0.026938  0.019562  0.013883  0.
020815  0.050537  0.037292  0.000000  0.039902  0.041623  0.041106
Zebra-----     0.027111  0.042638  0.020743  0.020926  0.014595  0.040547  0.040536  0.040566  0.034873  0.042373
 0.042575  0.021104  0.032024  0.021102  0.040565  0.021001  0.021104  0.027111  0.027131  0.046448  0.020903  0.
027419  0.040572  0.021104  0.041605  0.040658  0.020903  0.034062  0.034064  0.041482  0.027216  0.027102  0.
048560  0.014384  0.006885  0.020343  0.027820  0.020258  0.021060  0.034909  0.041848  0.027248  0.040574  0.
040550  0.014177  0.054901  0.039902  0.000000  0.057308  0.014057
Cod-------     0.061905  0.062700  0.058687  0.061834  0.065948  0.041913  0.041907  0.041867  0.043665  0.062435
 0.062637  0.052206  0.048968  0.066895  0.041905  0.066832  0.052206  0.061905  0.057192  0.047173  0.051831  0.
057360  0.034872  0.052206  0.045274  0.041893  0.051831  0.050766  0.050783  0.045290  0.058523  0.061950  0.
065933  0.050244  0.057289  0.058505  0.050848  0.058739  0.052148  0.053997  0.047641  0.040720  0.034838  0.
048928  0.067943  0.040836  0.041623  0.057308  0.000000  0.058512
Langur----     0.013702  0.034301  0.020836  0.021019  0.015594  0.041751  0.041740  0.041770  0.024669  0.040007
 0.040836  0.020904  0.033225  0.019940  0.041769  0.015710  0.020904  0.013702  0.020378  0.047652  0.027679  0.
013448  0.041776  0.020904  0.040966  0.041862  0.027679  0.020653  0.020929  0.041375  0.013807  0.013693  0.
037431  0.014802  0.014035  0.006934  0.020171  0.020676  0.020831  0.035196  0.038180  0.022171  0.041778  0.
041754  0.020686  0.056700  0.041106  0.014057  0.058512  0.000000
```
