# Supplementary material for: Alignment-free similarity analysis for protein sequences based on fuzzy integral
Source: Sci Rep. 2019 Feb 26;9:2775. doi: 10.1038/s41598-019-39477-8 (PMC6391537; doi:10.1038/s41598-019-39477-8)
Supplement: Supplementary file 3 — ROC_supplementary material [file 41598_2019_39477_MOESM3_ESM.pdf]

Alignment-free similarity analysis for protein sequences based on fuzzy integral.

Ajay Kumar Saw[1], Binod Chandra Tripathy[2], and Soumyadeep Nandi[3*]

[1]Institute of Advanced Study in Science and Technology, Mathematical Sciences Division, Guwahati-781035, India
[2]Tripura University, Department of Mathematics, Agartala - 799022, India
[3]Institute of Advanced Study in Science and Technology, Life Science Division, Guwahati-781035, India

[*]Corresponding author: soumyadeep.nandi@gmail.com

SUPPLEMENTARY MATERIALS

TABLES

Number	Species	ID(NCBI)	Length
1	Human(*Homo sapiens*)	AP 000649	603
2	Gorilla(*Gorilla gorilla*)	NP 008222	603
3	Common chimpanzee(*Pan troglodytes*)	NP 008196	603
4	Pigmy chimpanzee(*Pan paniscus*)	NP 008209	603
5	Fin whale(*Balenoptera physalus*)	NP 006899	606
6	Blue whale(*Balenoptera musculus*)	NP 007066	606
7	Rat(*Rattus norvegicus*)	AP-004902	610
8	Mouse(*Mus musculus*)	NP 904338	607
9	Opossum (*Didelphis virginiana*)	NP 007105	602

Table S1: The concise information for 9 ND5 protein sequences.

No.	Species	Accession no.	Length
1	Human (*Homo sapiens*)	YP_0030240	174
2	Gorilla (*Gorilla gorilla*)	NP_008223	174
3	Common chimpanzee (*Pan troglodytes*)	NP_008197	174
4	Harbor seal (*Phoca vitulina*)	NP_006939	175
5	Gray seal (*Halichoerus grypus*)	NP_007080	175
6	Rat (*Rattus norvegicus*)	AP_004903	172
7	Mouse (*Mus musculus*)	NP_904339	172
8	Wallaroo (*Macropus robustus*)	NP_007405	167

Table S2: The concise information for 8 ND6 protein sequences.

Sequence name	Species	Accession no.	Length
Human TF	*Homo sapiens*	S95936	698
Rabbit TF	*Oryctolagus coniculus*	X58533	695
Rat TF	*Rattus norvegicus*	D38380	698
Cow TF	*Bos Taurus*	U02564	704
Buffalo LF	*Bubahts arnee*	AJ005203	708
Cow LF	*Bos Taurus*	X57084	708
Goat LF	*Copra hircus*	X78902	708
Camel LF	*Camehts dromedaries*	AJ131674	708
Pig LF	*Sus scrofa*	M92089	704
Human LF	*H. sapiens*	NM 002343	710
Mouse LF	*Mus musculus*	NM 008522	707
Possum TF	*Trichosurus vulpecula*	AF092510	711
Frog TF	*Xenopus laevis*	X54530	702
Japanese flounder TF	*Pctralichthys olivaceiis*	D88801	685
Atlantic salmon TF	*Salmo salar*	L20313	690
Brown trout TF	*Salmo trutta*	D89091	691
Lake trout TF	*Salvelimts namaycush*	D89090	691
Brook trout TF	*Sahelinus fontinalis*	D89089	691
Japanese char TF	*Sahelinus phius*	D89088	691
Chinook salmon TF	*Oncorhynchus tshawytscha*	AH008271	677
Coho salmon TF	*Oncorhynchus kisuich*	D89084	691
Sockeye salmon TF	*Oncorhynchus nerka*	D89085	691
Rainbow trout TF	*Oncorhynchus mykiss*	D89083	691
Amago salmon TF	*Oncorhynchus masou*	D89086	691

Note: TF:- transferrin; LF:- lactoferrin

Table S3: The concise information for 24 TF protein sequences.

No.	Accession number	Name	Abbreviation	Group
1	CAB91145	Transmissible gastroenteritis virus, genomic RNA	TGEVG	I
2	NP_058424	Transmissible gastroenteritis virus	TGEV	I
3	AAK38656	Porcine epidemic diarrhoea virus strain CV777	PEDVC	I
4	NP_598310	Porcine epidemic diarrhoea virus	PEDV	I
5	NP_937950	Human coronavirus OC43	HCoV-OC43	II
6	AAK83356	Bovine coronavirus isolate BCoV-ENT	BCoVE	II
7	AAL57308	Bovine coronavirus isolate BCoV-LUN	BCoVL	II
8	AAA66399	Bovine coronavirus strain Mebus	BCoVM	II
9	AAL40400	Bovine coronavirus strain Quebec	BCoVQ	II
10	NP_150077	Bovine coronavirus	BCoV	II
11	AAB86819	Mouse hepatitis virus strain MHV-A59C12 mutant	MHVA	II
12	YP_209233	Murine hepatitis virus strain JHM	MHVJHM	II
13	AAF69334	Mouse hepatitis virus strain Penn 97-1	MHVP	II
14	AAF69344	Mouse hepatitis virus strain ML-10	MHVM	II
15	NP_045300	Mouse hepatitis virus	MHV	II
16	AAP92675	Avian infectious bronchitis virus isolate BJ	IBVBJ	III
17	AAS00080	Avian infectious bronchitis virus strain Ca199	IBVC	III
18	NP_040831	Avian infectious bronchitis virus	IBV	III
19	AAS10463	SARS coronavirus GD03T0013	GD03T0013	IV, IVa
20	AAU93318	SARS coronavirus PC4-127	PC4-127	IV, IVa
21	AAV49720	SARS coronavirus PC4-137	PC4-137	IV, IVa
22	AAU93319	SARS coronavirus PC4-205	PC4-205	IV, IVa
23	AAU04646	SARS coronavirus civet007	civet007	IV, IVa
24	AAU04649	SARS coronavirus civet010	civet010	IV, IVa
25	AAU04664	SARS coronavirus civet020	civet020	IV, IVa
26	AAV91631	SARS coronavirus A022	A022	IV, IVa
27	AAV49730	SARS coronavirus B039	B039	IV, IVa
28	AAP51227	SARS coronavirus GD01	GD01	IV
29	AAS00003	SARS coronavirus GZ02	GZ02	IV
30	AAP30030	SARS coronavirus BJ01	BJ01	IV
31	AAP13567	SARS coronavirus CUHK-W1	CUHK-W1	IV
32	AAP50485	SARS coronavirus FRA	FRA	IV
33	AAP41037	SARS coronavirus TOR2	TOR2	IV
34	AAQ01597	SARS coronavirus Taiwan TC1	TaiwanTC1	IV
35	AAQ01609	SARS coronavirus Taiwan TC2	TaiwanTC2	IV
36	AAP13441	SARS coronavirus Urbani	Urbani	IV
37	AAQ94060	SARS coronavirus AS	AS	IV
38	AAP30713	SARS coronavirus CUHK-Su10	CUHK-Su10	IV
39	AAP33697	SARS coronavirus Frankfurt 1	Frankfurt1	IV
40	AAP94737	SARS coronavirus CUHK-AG01	CUHK-AG01	IV
41	AAP94748	SARS coronavirus CUHK-AG02	CUHK-AG02	IV
42	AAP37017	SARS coronavirus TW1	TW1	IV
43	AAR87523	SARS coronavirus TW2	TW2	IV
44	BAC81348	SARS coronavirus TWH genomic RNA	TWH	IV
45	BAC81362	SARS coronavirus TWJ genomic RNA	TWJ	IV
46	AAP72986	SARS coronavirus HSR 1	HSR1	IV
47	AAR23250	SARS coronavirus Sin01-11	Sino1-11	IV
48	AAR23258	SARS coronavirus Sin03-11	Sino3-11	IV

49	AAR14803	SARS coronavirus PUMC01	PUMC01	IV
50	AAR14807	SARS coronavirus PUMC02	PUMC02	IV

Table S4: The concise information of 50 coronavirus spike protein sequences of animal species.

Animal names	Accession number	Animal names	Accession number
Human	AAA16334.1	Pigeon	P11342.1
Goshawk	P08851.1	Black bear	P68012.1
Lesser panda	P18982.1	Asiatic elephant	P02084.1
Giant panda	P18983.2	African elephant	P02085.1
Sheep	P02075.2	Tortoise	P83123.3
Duck	P02114.2	Grivet	P02028.1
Mallard	P02115.1	Gorilla	P02024.2
Goose	P02117.1	Shark	P02143.1
Rat	CAA33114.1	Hippopotamus	P19016.1
Penguin	P80216.1	Horse	P02062.1
Swift	P15165.1	Gibbon	P02025.1
Coyote	P60525.1	Whale	P18984.1
Catfish	O13163.2	Bat	P24660.1
Bison	P09422.1	Red fox	P21201.1
Swan	P68945.1	Marmot	P08853.1
Buffalo	P67820.1	Salmon	Q91473.3
Dog	P60524.1	Sparrow	P07406.1
Chimpanzee	P68873.2	Pheasant	P02113.1
Dolphin	P18990.1	Flamingo	P02121.1
Goldfish	P02140.1	Pig	P02067.3
Polar bear	P68011.1	Dragonfish	ADD73488.1
Rhinoceros	P09907.1	Parakeet	P21668.1
Chicken	P02112.2	Zebra	P67824.1
Wolf	P60526.1	Cod	O13077.2
Turtle	P13274.1	Langur	P02032.1

Table S5: The concise information of 50 beta-globin protein sequences of animal species.

FIGURES

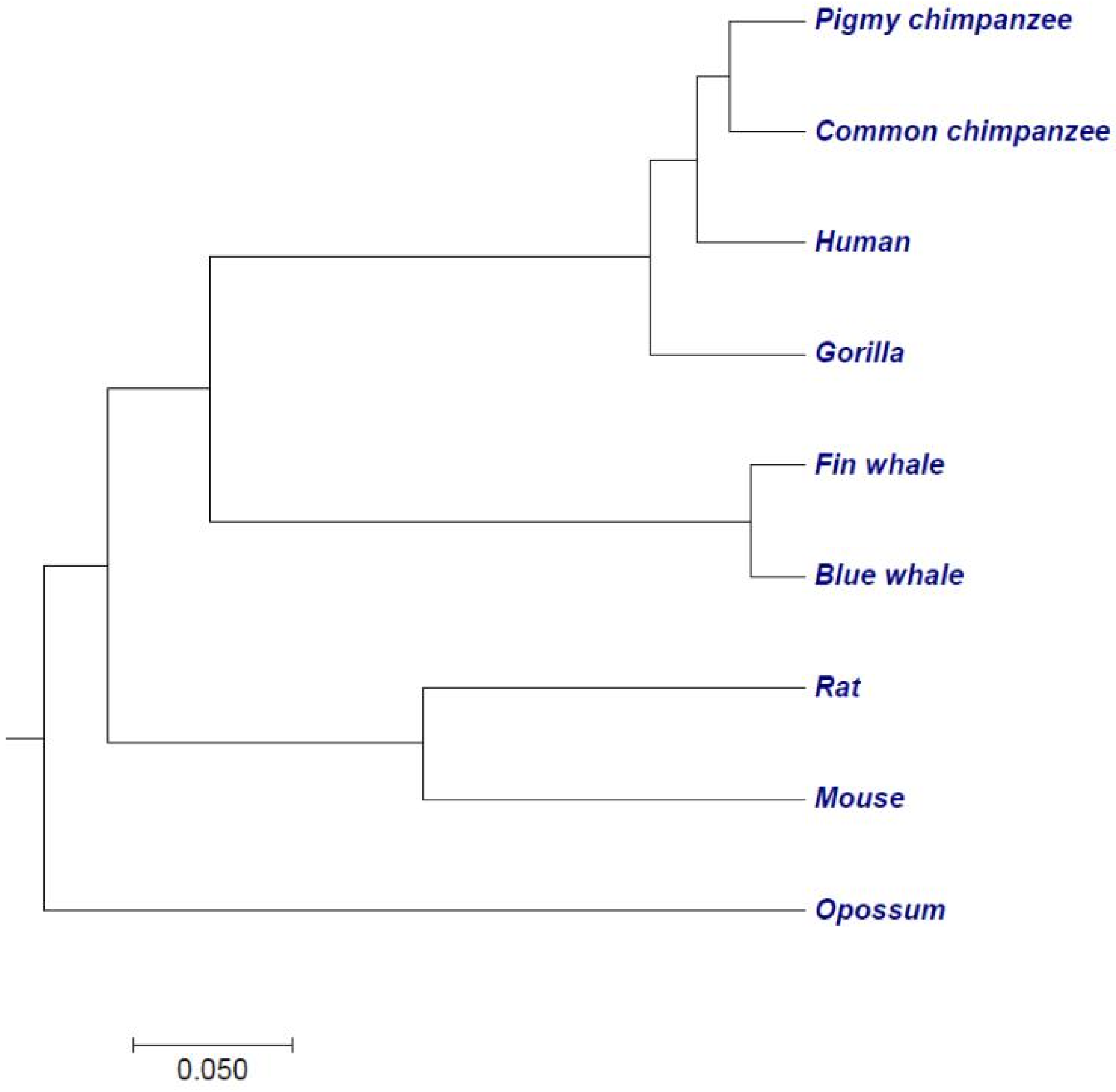

Figure S1: The phylogenetic tree for 9 sequences of ND5 protein constructed by ClustalW method using MEGA package[a].

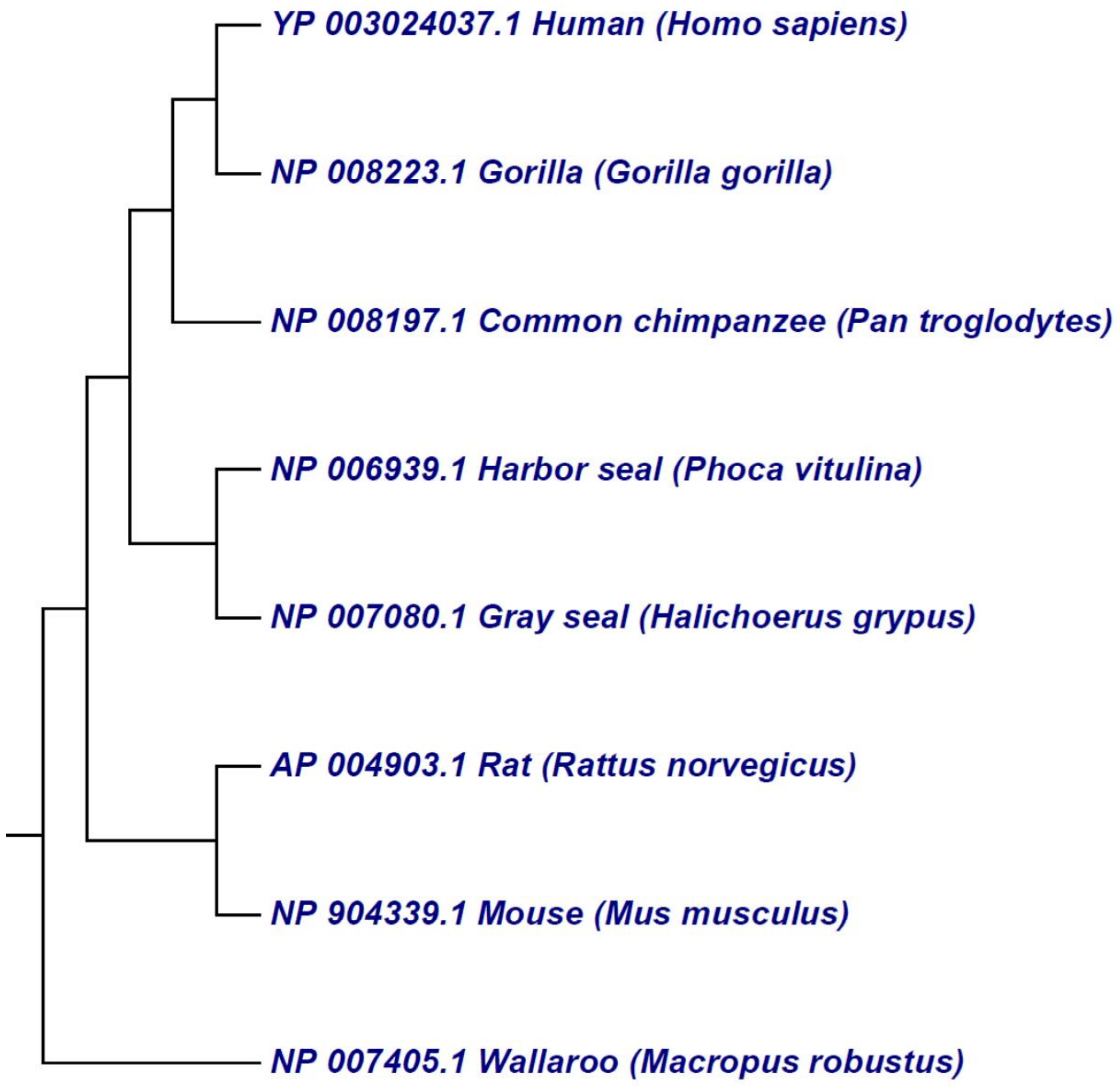

Figure S2: Phylogenetic tree of the 8 ND6 proteins constructed by ClustalW method using MEGA package[a].

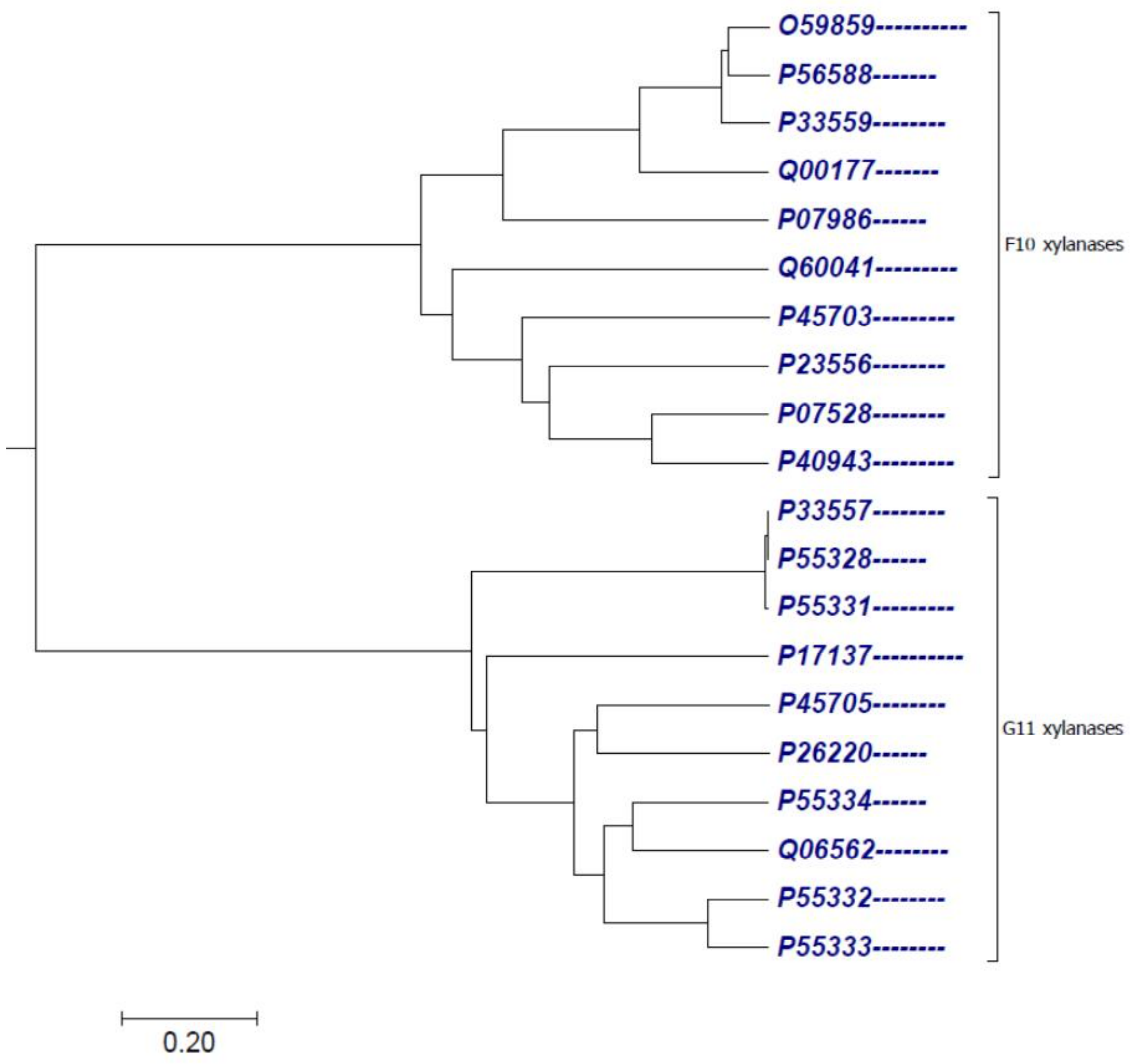

Figure S3: Phylogenetic tree using 20 xylanases in the F10 and G11 datasets generated by ClustalW method using MEGA package[a].

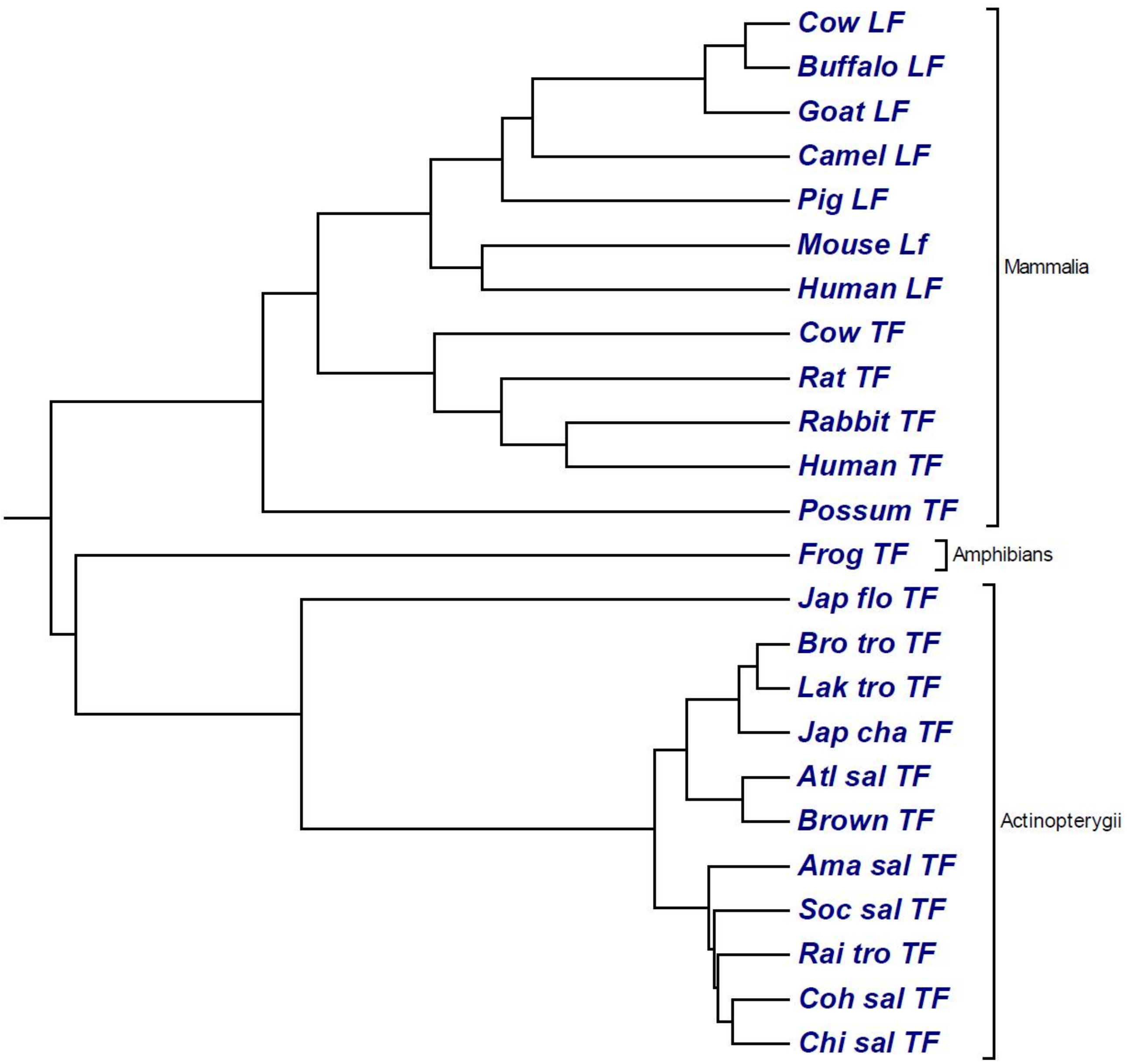

Figure S4: Phylogenetic tree of 24 TFs constructed by ClustalW method using MEGA package[a].

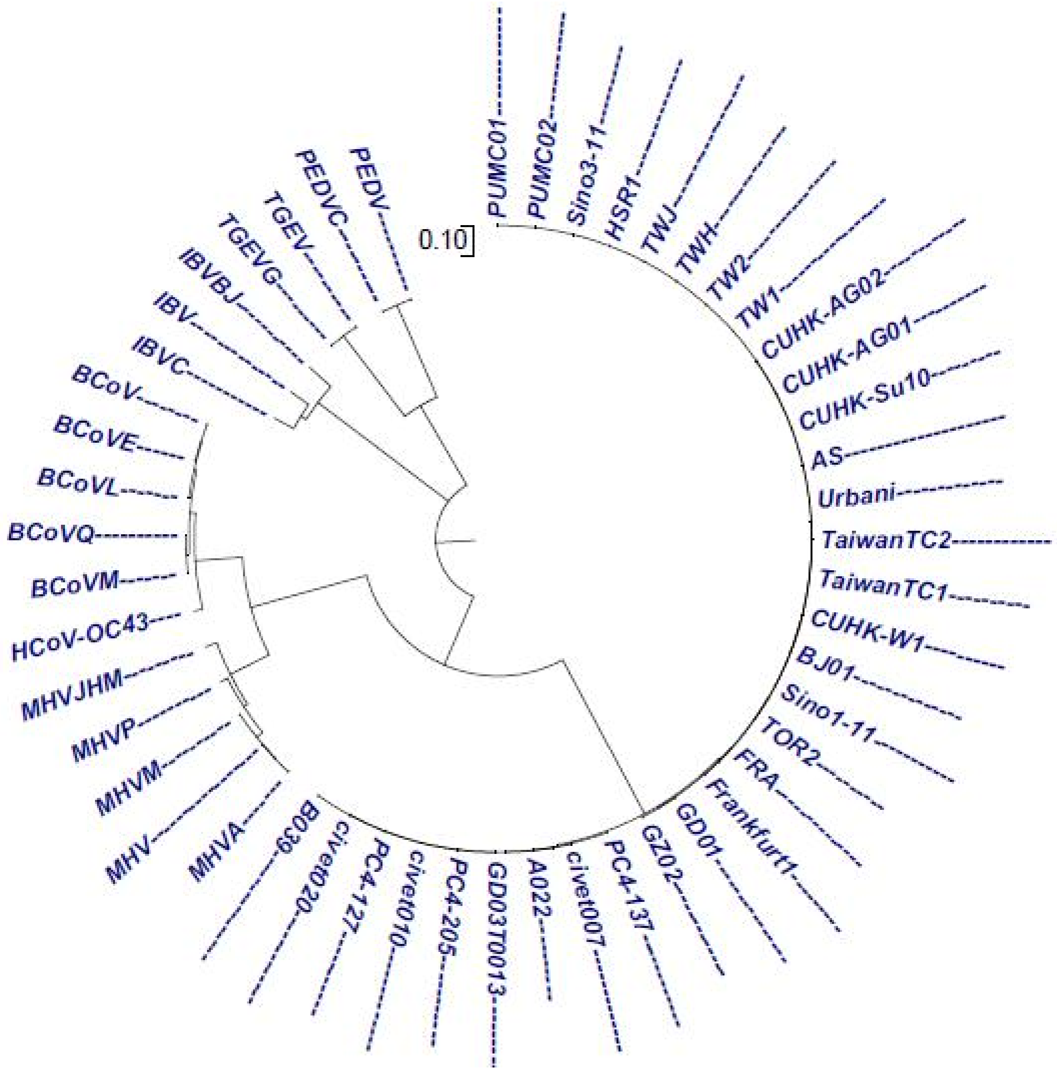

Figure S5: The phylogenetic tree of 50 coronavirus spike proteins based on ClustalW method using MEGA package[a].

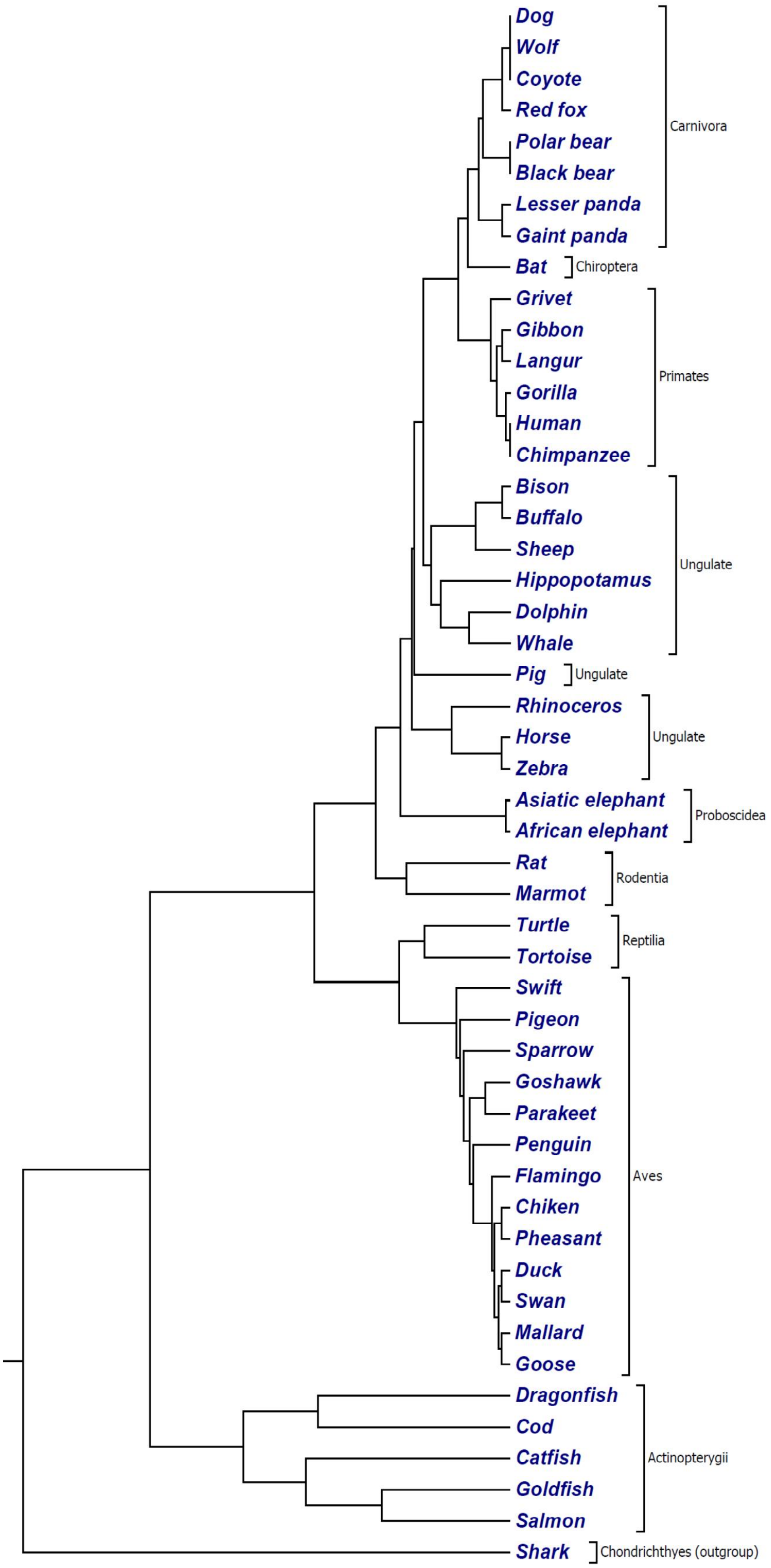

Figure S6: The phylogenetic tree for 50 sequences of beta-globin protein constructed by ClustalW method using MEGA package[a].

REFERENCE:-

[a]:- Kumar, S., Stecher, G. & Tamura, K. Mega7: Molecular evolutionary genetics analysis version 7.0 for bigger datasets. Mol.Biol. Evol. 33, 1870–1874 (2016).
